# Variation in recombination frequency and distribution across eukaryotes: patterns and processes

**DOI:** 10.1098/rstb.2016.0455

**Published:** 2017-11-06

**Authors:** Jessica Stapley, Philine G. D. Feulner, Susan E. Johnston, Anna W. Santure, Carole M. Smadja

**Affiliations:** 1 Centre for Adaptation to a Changing Environment, IBZ, ETH Zürich, 8092 Zürich, Switzerland; 2 Department of Fish Ecology and Evolution, Centre of Ecology, Evolution and Biogeochemistry, EAWAG Swiss Federal Institute of Aquatic Science and Technology, 6047 Kastanienbaum, Switzerland; 3 Division of Aquatic Ecology and Evolution, Institute of Ecology and Evolution, University of Bern, 3012 Bern, Switzerland; 4 Institute of Evolutionary Biology, University of Edinburgh, Edinburgh EH9 3JY, UK; 5 School of Biological Sciences, University of Auckland, Auckland 1142, New Zealand; 6 Institut des Sciences de l'Evolution UMR 5554, CNRS, IRD, EPHE, Université de Montpellier, 3095 Montpellier cedex 05, France

**Keywords:** crossing over, meiosis, genetic linkage, evolution, adaptation, genomic architecture

## Abstract

Recombination, the exchange of DNA between maternal and paternal chromosomes during meiosis, is an essential feature of sexual reproduction in nearly all multicellular organisms. While the role of recombination in the evolution of sex has received theoretical and empirical attention, less is known about how recombination rate *itself* evolves and what influence this has on evolutionary processes within sexually reproducing organisms. Here, we explore the patterns of, and processes governing recombination in eukaryotes. We summarize patterns of variation, integrating current knowledge with an analysis of linkage map data in 353 organisms. We then discuss proximate and ultimate processes governing recombination rate variation and consider how these influence evolutionary processes. Genome-wide recombination rates (cM/Mb) can vary more than tenfold across eukaryotes, and there is large variation in the distribution of recombination events across closely related taxa, populations and individuals. We discuss how variation in rate and distribution relates to genome architecture, genetic and epigenetic mechanisms, sex, environmental perturbations and variable selective pressures. There has been great progress in determining the molecular mechanisms governing recombination, and with the continued development of new modelling and empirical approaches, there is now also great opportunity to further our understanding of how and why recombination rate varies.

This article is part of the themed issue ‘Evolutionary causes and consequences of recombination rate variation in sexual organisms’.

## 1. Introduction

Recombination is the exchange of DNA between maternal and paternal chromosomes during meiosis, and is a fundamental feature of sexual reproduction in nearly all multicellular organisms, producing new combinations of genetic variants or alleles that are passed on to offspring. It is also a fundamental, yet paradoxical evolutionary process: it can facilitate adaptation through the creation of novel genetic combinations, but it can also break apart favourable combinations of alleles, potentially reducing fitness [1–3]. This antagonism is central to the adaptive responses of organisms to their environment [4,5], but also to the evolution of sex [3,6] and to the formation of new species when there is gene flow [7,8]. Recombination also performs an essential role during meiosis to ensure accurate segregation of chromosomes [9,10]. As a consequence, tight regulation of the rate of recombination is expected, but studies have revealed that recombination can vary within and between chromosomes, individuals, sexes, populations and species [11–15]. Recombination rates can be influenced by environmental and demographic factors, but are also heritable and underpinned by specific genetic loci [16–20] and can respond to selection [21,22]. Therefore, they have the potential to vary in a manner dependent on the evolutionary or selective contexts [6]. While the role of recombination in the evolution of sex and in facilitating responses to selection has been the focus of much empirical and theoretical work, investigation on how recombination rate *itself* evolves and how this impacts evolutionary processes within sexually reproducing organisms has received less attention. Until recently, empirical studies were restricted to cytogenetic studies of chiasma counts, or to low-density linkage map data in a handful of model organisms; however, in recent years, advances in genomic technologies have allowed more detailed characterization of recombination rates at a finer genomic scale and in a greater number of species.

In this review, we aim to explore the patterns of, and processes governing recombination in predominantly sexually reproducing eukaryotes from an evolutionary perspective, in a manner that is accessible to a general audience. We begin by summarizing the patterns of variation in the number of recombination events in the genome per megabase per generation (herein referred to as recombination rate) at different taxonomic and genomic scales across eukaryotes—updating and integrating current knowledge with an analysis of linkage map data in 353 organisms. Then, we discuss processes governing recombination rate variation, beginning with what is known of the proximate causes and genetic correlates of recombination rate variation, before summarizing the key evolutionary (ultimate) causes and consequences of this variation. We do not attempt to systematically review the enormous body of literature, but want to provide the reader with an introduction to the topic that is taxonomically broad, reflecting the development of the field, and provide directions for future research. Throughout, we use the term recombination to refer to the meiotic process whereby a double-strand DNA break (DSB) is repaired via reciprocal exchange of genetic material between homologous chromosomes, resulting in a crossover (CO).

## 2. Patterns of variation in recombination

**Figure 1.**
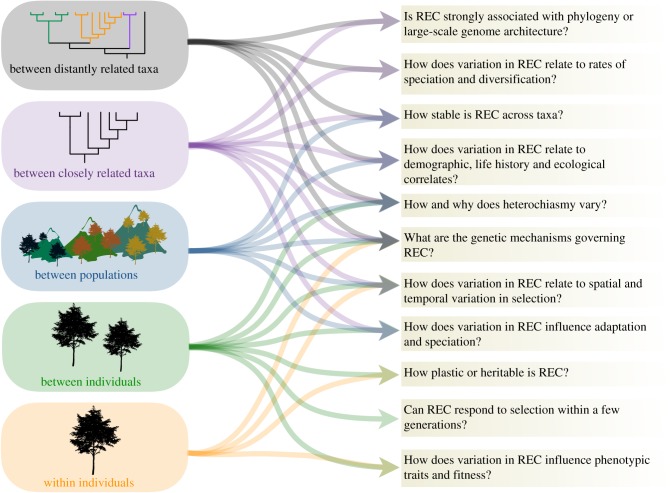
Comparing recombination landscape and frequency (REC) across different taxonomic and spatial scales (boxes on the left) provides complementary data to address outstanding questions about how and why recombination varies (boxes on right).

Recombination can be compared at different taxonomic scales and at different genomic resolutions, and information at these different scales provides opportunities to address different questions about how and why recombination rate varies (figure 1). Recent advances in DNA sequencing technologies and in methods to estimate recombination rate from genetic variation data (polymorphisms) sampled from a population have facilitated estimates of the genome-wide recombination rate (GwRR) across species and provided new opportunities to determine the distribution of recombination at a finer genomic scale (see box 1). A pervasive pattern to emerge from these studies is that recombination events are distinctly non-random, and two important patterns are recognized. First, the exchange of DNA during a CO event at a location on the chromosome (known as a chiasma) tends to suppress the creation of nearby chiasma, in a process known as CO interference [40], and, second, recombination events are often localized into narrow regions, termed hotspots, where recombination is an order of magnitude (2–10×) higher than the average. Hotspots have been observed in a range of organisms, e.g. *Saccharomyces* yeast [41], fungal pathogens [42], plants (see [43]), mammals [44] and birds [45], but are absent from others, e.g. *Caenorhabditis elegans* [46], honeybees [47] and *Drosophila* (see [48,49]). Studies across different taxonomic scales have shown that recombination frequency and landscape may be controlled by different mechanisms in different taxa. Consequently, describing how recombination frequency and landscape vary at different taxonomic scales, from distantly related taxa to individuals, is a key step towards understanding their rate of evolution as well as their proximal and ultimate correlates.

Box 1. **Estimating recombination rate.**

Two parameters can describe how patterns of recombination vary between any two individuals or groups of individuals: the GwRR (how often COs occur e.g. in a given meiosis) and the recombination landscape (where COs occur in the genome). These estimates of recombination rate are commonly expressed as the recombination frequency per mega- or kilobase per generation [11,23–27] and can be estimated at different genomic resolutions. Historically, recombination rates were estimated by directly counting the number of chiasmata during meiosis using cytogenetic methods, and from early linkage maps, where phenotypes and/or genetic markers were ordered along chromosomes based on the frequency at which they were co-inherited (i.e. not separated by a CO). A spacing of one centimorgan (cM) indicates a one per cent chance that two genes will be separated by crossing over. Both approaches provided coarse-scale estimates of the recombination frequency, but lacked accuracy. In particular, linkage map estimates of recombination require pedigree information and are limited by the number of independent meioses characterized (i.e as a function of sample size, pedigree size and depth), and if marker densities are low, they fail to capture all COs and underestimate map length [28,29]. Low-resolution estimates of recombination provide limited information about the recombination landscape, but can provide useful data for looking at large-scale differences between chromosomes, chromosome arms or chromosome segments. These estimates also provide common measures that are comparable across larger taxonomic scales.

Today, the resolution to determine recombination rates and landscapes has dramatically improved with developments in high-throughput sequencing and genotyping technologies. It is now feasible to obtain estimates of recombination rate on a finer genomic scale, with dense linkage maps and population-scaled estimates of recombination rate. While linkage maps provide an estimate of COs observed over a few generations, population-scaled approaches provide estimates of historical recombination [30]. This approach uses high-density marker and/or genome sequence data to estimate population-scaled recombination rates (*ρ*) using coalescent methods that model patterns of linkage disequilibrium (LD), the non-random association of alleles across loci, within narrow genomic regions. These approaches have been used to identify recombination ‘hotspots’. A limitation of coalescent estimates is that LD is also affected by the effective population size of a population, which is influenced by the population's demographic history (e.g. bottlenecks, gene flow, selection (e.g. [31])). However, new developments in population-based approaches are implementing ways to account for demographic history during recombination rate inference (e.g. [32,33]).

Despite their differences, results from linkage map and population-based estimates are highly correlated [31,34–37]. It is also important to note that all marker-based estimates (linkage map and population-based estimates) can only detect a recombination event that results in a change in the allelic combination in the next generation (effective recombination)—for example, if parents are homozygote across many markers, the action of recombination is not detectable and recombination is typically only measured from gametes that successfully produced offspring (realized recombination). One method to quantify recombination events in all gametes, not just those that produce offspring, is to genotype or whole-genome sequence single sperm. For example, in humans this approach has been used to fine-map the recombination landscape and investigate transmission distortion and allelic drive [38], and in *Daphnia* it was used to build a genetic linkage that helped to improve the genome assembly [39].

### (a) Variation across distantly related eukaryote taxa

There have been several comparisons of the GwRR per base, kilobase (Kb) or megabase (Mb) across distantly related taxa [23,24,50,51]. The most striking pattern to emerge was that microorganisms and fungi have much higher recombination rates compared to animals and plants [23,24]. However, these studies were carried out in a relatively small number of species, often relying on chiasma count data in a single sex. Therefore, we compiled data on linkage map length, haploid chromosome number (HCN) and genome size from all the major groups of eukaryotes, to provide a more comprehensive and up-to-date picture of recombination rate variation. Details of the methods and data are provided in the electronic supplementary material, and a summary of the species included in our dataset is in table 1 (see the electronic supplementary material for full list). Briefly, we obtained sex-averaged linkage map lengths, genome size and HCN from the published literature and public databases. In cases where a species had multiple maps, we chose the map with the most markers or the most individuals in cases where two maps had a similar number of markers. We omitted linkage maps with fewer than 50 markers and where the number of linkage groups (LG) and the HCN differed markedly (absolute(LG–HCN)/HCN > 0.7). In our analyses, we controlled for phylogeny by fitting a Phylogenetic Generalized Linear Model with the R Package ‘Caper’ [52]. The phylogeny was obtained using the Phylotastic Web Service (https://github.com/phylotastic/phylo_services_docs/blob/master/ServiceDescription/PhyloServicesDescription.md), which extracts a Supertree from openTree [53]. All branch lengths were set to 1 in the Supertree. In total, we obtained data for 353 species, across animals, plants, fungi and the SAR (Stramenopiles-Alveolates-Rhizaria Eukaryote) supergroup. Not surprisingly, there is a bias towards model species, domestic and crop species, and parasitic or disease-causing species, for which quantitative trait locus (QTL) mapping studies have been the focus of much research.

**Table 1.** Summary of the linkage map data compiled from the literature; linkage map length (centimorgans, cM), genome size (megabases, Mb), haploid chromosome number and recombination rate (cM/Mb). SAR, Stramenopiles-Alveolates-Rhizaria Eukaryote.

group	*n*	linkage map length (cM)			genome size (Mb)			haploid chromosome number			recombination rate (cM/Mb)		
		mean	min	max	mean	min	max	mean	min	max	mean	min	max
SAR	9	1782	653	2884	189	18.87	560	18.78	9	34	38.67	3.24	108.00
fungi	15	2068	86	5860	49.26	19.05	170.2	13.27	4	21	48.68	1.40	119.90
animals	140	1813	90	5961	1538	43.15	30 880	22.27	3	73	2.52	0.12	28.10
plants	189	1567	309	8184	2956	120.40	29 280	13.91	5	90	1.85	0.03	9.22
total or mean	353	1807.5			1183.0			17.05			22.93		

**Figure 2.**
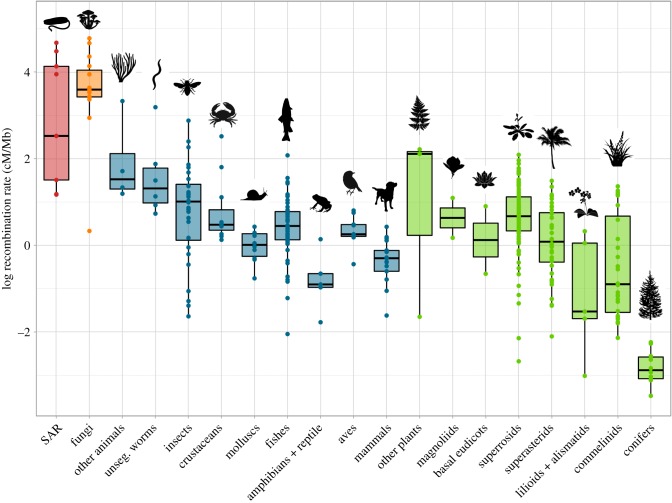
Variation in the log of recombination rate, estimated by dividing linkage map length in centimorgans (cM) by genome size (Mb) across eukaryotic taxa. Other plants: Pteridophyta, Chlorophyta, Bryophyta. Other animals: Anthzoa, Holothuriodea, Ascidacae. unseg, unsegmented.

To estimate the GwRR from linkage map data, we divided the linkage map length (the sum of the length of all sex-averaged LGs) by the haploid genome size (in Mb) (box 2 and figure 2). This is a commonly reported measure of recombination rate [11,23–27] and provides a useful metric to compare across taxa with vastly different genome sizes. This measure averages recombination across both the open and transcriptionally active euchromatic region and the closed and inactive heterochromatic regions of the genome. Recombination is often suppressed in heterochromatic regions, and the strength of suppression and the proportion of the genome that is heterochromatic vary greatly between organisms (see [69]). Thus, the GwRR represents a genome average that reveals differences in recombination rate, but will be related to differences in the amount of heterochromatin in the genome and how strongly recombination is suppressed in these regions. Taking account of the proportion of the genome that is heterochromatic may provide more informative estimates of recombination with respect to evolutionary processes [27,69]; however, these data are only available for relatively few organisms, so we have not included them in this analysis. Overall, our analysis confirms the previously reported pattern of a higher GwRR in fungi and SAR compared to plants and animals, but also provides estimates for new taxonomic groups (electronic supplementary material, figures S1–S3) and opportunities to begin to address enduring questions about the evolution of recombination rate (figure 1).

Box 2. **How does recombination rate vary with genome architecture?**

**Genome size**

**Figure 3.**
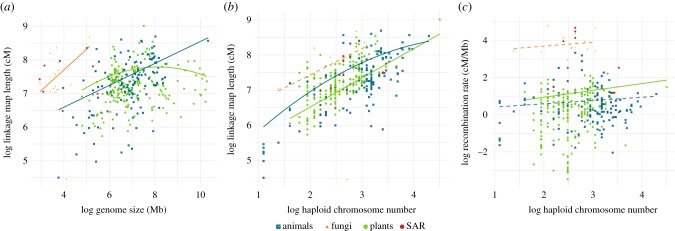
Observed (points) and fitted (lines) relationships between: (*a*) log genome size (megabases, Mb) and log linkage map length (centimorgans, cM), (*b*) log haploid chromosome number (HCN) and log linkage map length (cM) and (*c*) log HCN and log recombination rate measured as linkage map (cM) divided by genome size (Mb). Fitted linear and quadratic relationships were obtained by fitting a phylogenetic generalized linear model separately for plants, animals and fungi.

Following the observation that linkage map length was similar across eukaryotes despite large variation in genome size, it was proposed that larger genomes have several orders of magnitude lower recombination rates [51]. This is consistent with the observed relationships between recombination rate and sequence features; recombination rate is positively correlated with gene density and negatively with the density of repetitive elements, which could drive lower recombination rates in large, repeat-rich genomes [27,54]. Higher recombination rates can also lead to reductions in genome size—if recombination rate increases the mutation rate and small deletions are more common than small insertions (mutational bias), purifying selection on these mutations can drive genome contraction [55,56]. Both positive and negative relationships between genome size and recombination rate have been found (positive [57], negative [24,27,50]). The disparity in results may be attributed to differences in the methods used and taxonomic breadths considered, but may also be due to statistical problems. When the recombination rate is calculated as the linkage map length (cM) divided by genome size (Kb or Mb), then genome size and recombination are mathematically coupled; it is not appropriate to test for relationship between mathematically coupled variables [58]. To investigate the relationship between genome size and recombination rate, we examined the fit of linear and quadratic relationships between linkage map length and genome size, while controlling for phylogeny. In animals and fungi, a linear model best fit the data, but in plants, a quadratic model was a better fit (figure 3*a*; electronic supplementary material). This suggests that recombination rate is lower in larger plant genomes, but in animals and fungi there is no evidence to suggest recombination rate declines with genome size.

**Haploid chromosome number**

The number and size of chromosomes can explain variation in the GwRR because a minimum of one CO per chromosome (or chromosome arm) is often required to ensure proper segregation of chromosomes during meiosis [13,59–62]. There are several exceptions: some chromosomes do not recombine in one sex (e.g. achiasmate species; see §2c) and often more than one CO per chromosome is observed on larger chromosomes (see [62]). Under the obligate CO requirement, a higher recombination rate could be achieved by increasing the number of chromosomes or by having smaller chromosomes; bird genomes, containing many microchromosomes, provide support for this hypothesis [37,63]. Whether karyotypic variation is driven by selection on recombination rate is unclear (e.g. [64,65]), but Burt [66] demonstrated that an increase in the efficacy of selection was better achieved by increasing the number of COs per chromosome rather than increasing the number of chromosomes. Whole-genome duplication and polyploidy are dramatic ways to increase chromosome number, and under an obligate CO requirement, this should result in at least a doubling of chiasma frequency. Polyploids' ability to achieve stable meiosis may be partly due to a reduction in the GwRR (and increase in interference distance) to ensure only one CO per pair of homologous chromosomes, as a mechanism to avoid the pairing of three or more homologous chromosomes [67,68]. The data we compiled provide an opportunity to test whether HCN explains variation in linkage map length and the GwRR (cM/Mb) across eukaryotes. A positive linear relationship between linkage map length and HCN was found for plants and fungi, while in animals a quadratic relationship was slightly better at explaining this relationship (figure 3*b*; electronic supplementary material). We found that the HCN was not related to GwRR (per Mb) in fungi and animals, and although a relationship was found in plants, the amount of variation explained was low (*r*^2^ = 0.02) (figure 3*c*; electronic supplementary material). Despite explaining little variation, we do suggest that scaling the GwRR by HCN provides a useful comparative measure of recombination rate and removes variation attributable to the obligate CO requirement.

In contrast with comparing recombination rate across distantly related taxa, comparisons within specific taxonomic groups are more common (i.e. mammals [59], plants [25,27,57,70], homopterous insects [71] and hymenoptera [64]), and several notable patterns have been identified. For example, among insects, social hymenoptera have much higher recombination rates [23]; among mammals, marsupials have lower recombination rates [72]; and among plants, conifers have very low recombination rates [25]. Comparing within taxonomic groups in our data, we also observed these patterns and make several new observations: among Crustaceans, the Cladocerans (represented by two species of Daphnia) have much higher recombination rates (electronic supplementary material, figure S1), Dipterans have the lowest rates of recombination rate across insects (electronic supplementary material, figure S1) and fishes have the highest recombination rate among vertebrates (figure 2).

### (b) Variation among closely related taxa and between populations within species

Linking variation in recombination rates between closely related species and between populations with variation in selection and demography may elucidate long-term mechanisms driving recombination rate evolution. Differences in chiasma count between sister species, populations, accessions and inbred lines of cultivated and model species have been studied since the 1930s (e.g. [73–76]). Within a more ecological context (i.e. natural populations, non-model species), early empirical work identified relationships between chiasma frequency and ecological and environmental variables. For example, chiasma frequency per bivalent (Cf/B) in orthopterans is associated with latitude (see [15]) and was higher in low-density populations of grasshoppers [77] and snails [78], and in plants Cf/B was higher in selfers compared to outcrosses [79,80]. In many cases where clinal variation in recombination has been detected, karyotypic differences, which are known to modify recombination, are also present (e.g. accessory or B chromosomes (see [15,78]), chromosomal inversions [78]). These karyotypic differences can suppress the GwRR and may explain the variation observed. At a finer genomic scale, comparisons between closely related taxa find, in general, greater variation in the recombination landscape compared to the GwRR. For example, similar linkage map lengths are evident across species (e.g. *Eucalyptus* [81], flycatchers (*Ficedula)* [34]), strains (e.g. *C. briggsae* [82]), cultivars (e.g. maize (*Zea mays*) [83]) and populations (e.g. great tit (*Parus major*) [84], honeybee (*Apis mellifera*) [85]). In most mammals, the position of hotspots appears to be dynamic, differing between subspecies of mice [86] and between humans and chimps [32], while hotspot location is more conserved in other groups, for example birds [34,45], dogs [35] and in *Saccharomyces* yeast [87]. Recent work in determining the molecular mechanisms governing hotspot activity has shed light on this pattern: most notably, in species with rapidly evolving hotspots, hotspot position is determined by a common gene (*PRDM9*), whereas this gene is missing or non-functional in species with more conserved hotspots ([88] and discussed in §3b).

### (c) Variation in recombination between the sexes

The most widely reported within-species variation in recombination rate is that seen between the sexes. Differences between sexes can be as extreme as one sex lacking recombination completely (achiasmy), or where recombination is present but different in both sexes, in terms of the rate and landscape (heterochiasmy), [89]. Achiasmy has evolved independently at least 26 times [15,90,91] and nearly always occurs in the heterogametic sex (e.g. in XY *Drosophila* males and ZW *Bombyx* females) [92–94]. By contrast, heterochiasmy is phylogenetically dispersed across plants and animals, and reduced recombination is not always observed in the heterogametic sex [89,90]. In animals and plants, females tend to have higher overall rates of recombination, although exceptions exist, such as in corals, marsupials, macaques and sheep [89,95,96]. There appears to be no link between sex chromosomes or sex-determining mechanism (genetic, environmental) and the direction of heterochiasmy. However, only one species that has environmental sex determination (ESD) has been studied to date, and more studies are needed in clades that have evolved ESD multiple times (e.g. lizards and turtles) to test this more explicitly.

### (d) Variation in recombination between individuals

Examination of recombination at the individual level, using cytogenetic and pedigree-based approaches, has shown that GwRRs can vary substantially between individuals within a population. Studies in humans, cattle, sheep, mice and *Drosophila* have shown that variation in regional or GwRRs (cM/Mb) often have an underlying heritable component, explaining 8–40% of the phenotypic variance in rate [16–18,97,98]. Mammalian studies have identified meiotic genes that consistently underlie rate variation, notably ring finger protein 212 (*RNF212*); studies at finer genomic scale, e.g. in humans and cattle, have also exposed heritable differences in recombination landscape and hotspot usage mediated by variation in *PRDM9* [18,36]. We explain these genetic mechanisms driving heritable variation in more detail in §3b.

### (e) Variation within individuals

Variation in recombination rate has been observed within individuals, i.e. between subsequent measurements or between clones experiencing different environments, demonstrating plasticity in recombination rate. Intrinsic factors, such as age and stress, as well as a diverse range of extrinsic factors, such as parasites, have been found to influence CO frequency [99–101]. Of all studies to date, there are three commonly reported factors affecting recombination rate within individuals.

The first, age, has been considered in several model species, but there is little consensus in broad trends. In humans, the recombination rate (cM/Mb) tends to increase with maternal age, while there appears to be little effect of paternal age (see [102] and references therein; for an exception see [103]); in mice, patterns in females and males are varied [104–107]. In *Arabadopsis thaliana*, paternal recombination rate (cM/Mb) measured at nine genomic intervals was stable in five of these regions, but increased with age in the other four [99]. In cattle and humans, CO interference, which can set a minimum distance between neighbouring COs, decreases with maternal age, which may explain observed increases in recombination frequency [106,108].

Second, temperature is one of the most commonly reported extrinsic correlates of recombination rate variation. In exothermic organisms, successful completion of meiosis is sensitive to changes in temperature, which are frequently associated with failures in synapsis and subsequent declines in fertility (see [109]). The relationship between increasing temperature and CO number and positioning varies across species; for example, in plants it is associated with increased paternal recombination in *Arabidopsis* and barley (*Hordeum vulgare* L.), but decreases in other species (e.g. *Allium ursinum*, *Locusta migratoria*) (see [99]). Relationships can also vary nonlinearly with temperature, such as in *Drosophila*, where the highest recombination rates occur at both high and low temperature extremes (see [109]). Interestingly, temperature can also influence the degree of heterochiasmy; in barley, at 10°C, sex-specific rates of recombination (cM/Mb) estimated from linkage maps were similar with a male/female map length ratio of 1.02, but at 30°C this ratio increased to 1.58 [110].

The third factor frequently associated with variation in recombination rate is pathogen infection. In line with predictions of the Red Queen hypothesis—enhanced recombination rates will increase the genetic diversity of offspring, so that more rapidly evolving parasites cannot exploit a static host genotype [111]—studies have observed longer linkage maps, and increased recombination frequency and rate (cM/Mb) with parasite infection; e.g. *Tribolium castenatum* [112,113], *Arabidopsis* [114] and tomato and barley [115], but see other studies in e.g. mice [116] and *T. castenatum* [117]). A study in *D. melanogaster* showed increased production of recombinant offspring in response to two bacteria and to a parasitic wasp, and this increase was driven by transmission distortion of recombinant chromatids—either during meiosis or due to asymmetric viability of gametes [97].

## 3. Molecular mechanisms governing variation in recombination rate

Meiosis evolved in the early history of eukaryotes, and many of the core mechanisms governing meiosis are highly conserved across the group [70,118,119]. Recombination is initiated by a DSB generated by SPO11 endonuclease, which is a DNA-binding domain (see [120]). Most DSBs are repaired via a non-crossover (NCO) pathway, which results in gene conversion rather than the exchange of DNA between chromosomes (e.g. only 5% of DSBs are repaired by CO in *Arabidopsis* [43], *ca* 10% in mice [121] and *ca* 60% in yeast [122]). Recombination is, therefore, a function of DSB formation and also a function of processes that govern CO versus NCO. Multiple factors govern the position of the DSB at multiple genomic scales; from the chromosome/sub-chromosomal regions to variation in the DNA sequence. DSBs occur predominantly within the euchromatic regions of the chromosome, preferentially in the chromatin loops, and are associated with several sequence features, with these mechanisms working hierarchically (see [118,119]). For example, two identical DNA sequences can experience markedly different recombination frequencies if they occur within different chromatic regions [119]; likewise, an active initiation site can lose its activity if it is inserted into a region with low DSB activity [123]. In this section, we review the genetic and epigenetic factors that are associated with variation in recombination, reflecting this hierarchy; we start at the broad genomic scale, and move to DNA sequence and epigenetic levels.

### (a) How does genomic architecture relate to recombination?

The GwRR has often been attributed to variation in the underlying genomic architecture, namely genome size, HCN, changes in ploidy, chromosome size and chromosomal rearrangements. Although a negative relationship between genome size and recombination rate is often assumed, there are limited robust data in support of this (see box 2). Our analysis of linkage map data across eukaryotes suggests little evidence that recombination rate decreases with genome size in fungi and animals, but that larger plant genomes have reduced recombination rates (figure 3*a* and box 2). It should be noted that our data average across genomes with different chromosome numbers and across hetero- and euchromatic regions. In addition, we did not include data on the proportion of the genome that is heterochromatic; however, we did explore the relationship between HCN and recombination rate. Although the HCN explains variation in the total number of recombination events across a genome, i.e. the linkage map length (figure 3*b*), it explains little variation in recombination rate per megabase (cM/Mb) (figure 3*c*). Our analysis suggests that genome architecture may play a limited role in driving variation in recombination rate at a broad scale (after controlling for phylogeny), which is consistent with the prediction that changing the number of COs per chromosomes is more effective at changing the efficacy of selection compared to changing the number of chromosomes [66].

Considering variation between chromosomes, recombination can be absent or greatly reduced on entire chromosomes (i.e. absent in one sex (achiasmate)) or on certain autosomes (e.g. *D. melanogaster* Chr 4 and *Toxoplasmodia gondii* Ch1a [124]), but also influenced by the presence of chromosomal rearrangements, such as inversions, fissions, fusions and translocations. Inversions represent a well-known case of rearrangement that can modify recombination: recombination is suppressed in individuals that are heterozygous for the inversion (heterokaryotype), because the inversion causes problems with pairing and segregation during meiosis [125]. This local suppression of recombination can also modify the recombination landscape in the longer term, so that suppression can extend to individuals homozygous for the inversion and to other, non-rearranged chromosomes (e.g. [20,126–129]). Such a long-term suppression of recombination due to strong selection may be achieved through a reduction in hotspot loci in the inverted and rearranged regions, which persists beyond the heterozygous state of such rearrangements [126].

One broad-scale and general pattern observed within chromosomes is a lower recombination rate around centromeres. While this could be attributed to selection against recombination in highly repetitive regions, repetitive sequence is not necessary for suppression; organisms that have no or few centromeric repeats also show suppressed recombination at the centromere [130]. Suppression is probably driven by chromatin structure; DSBs are less common in condensed heterochromatin, and the chromatin environment can influence the probability that a DSB is repaired with an NCO rather than a CO [118]. Recently, Talbert & Henikoff [130] argued that a DSB and repair via an NCO may be common in centromeres, and this could explain the accumulation of repetitive elements and diversification of centromeres, despite apparently few COs. Differences in the chromatin structure between males and females may also explain sex differences in the GwRR in mammals; for example in mice, females have longer bivalents (less compact chromatin) and have greater CO number [131]. Although heterochromatic regions are often difficult to sequence and study, it is likely they can provide important insights into factors influencing CO and NCO repair mechanisms and recombination.

### (b) Fine-scale molecular genetic mechanisms related to determining recombination

The genome architecture and chromatin structure clearly influence large-scale patterns in recombination, but what do we know about the patterns at smaller genomic scales? Recombination frequency and position covary consistently with several DNA sequence features; it is positively correlated with GC content and gene density and negatively correlated with transposable element (TE) density, and it is also consistently related to a number of gene regulatory elements and to histone modification (i.e. methylation) (for review, see [27,54,118,132]). Determining cause and effect from these correlations is problematic (see [54] for discussion about TEs). For example, recombination may drive increases in GC content via biased gene conversion in DSB repair in, for example, mammals [133], insects [134], birds [135] and rice [136]. However, in yeast, AT to GC substitutions are not directly correlated with recombination [137] and GC content may be a modifier of recombination [138]. Within genic regions, DSBs and subsequent recombination are more common in gene promoters or in regions with promoter-like features (see [43,70,118,120]).

In mammals and plants, several specific genetic mechanisms underlying variation in recombination rate have been identified. For example, *RNF212* (and its paralogue *RNF212B*), meiotic recombination protein REC8, and E3 ubiquitin–protein ligase *CCNB1IP1* homologue *HEI10*, have been consistently associated with rate in maize, yeast, *Arabidopsis*, cattle, humans, mice and sheep [16,18,83,98,139–141]. Research in mice has shown that *RNF212* is essential for crossing-over, with a key role in synapsis and the formation of recombination complexes specific to COs [142], whereas *HEI10* plays an antagonistic role that is essential for regulating NCO/CO processes [139]; studies suggest that these proteins have a dosage-dependent effect on CO rates.

As most recombination occurs in hotspots, understanding what governs hotspot position is highly relevant to revealing the genetic mechanisms governing recombination. The post-translational modification of histones, in particular trimethylation of lysine 4 on histone 3 (H3K4me3), is associated with DSB in many species [43,88,118,120,143]. The regulatory element positive-regulatory (PR) domain zinc finger protein 9 (*PRDM9*), which can modify H3K4, has been shown to drive DSB formation in mice and humans [17]. Not all H3K4me3 sites are recombination hotspots and many species lack functional copies or orthologues of *PRDM9* (e.g. *Drosophila*, yeast, dogs, birds and most plants), demonstrating that other mechanisms most certainly exist. In *Arabidopsis,* DNA methylation of H3K9me2 can suppress euchromatic CO hotspots [144]. There are likely to be at least two classes of hotspots: ancestral—occur in a wide range of organisms, are temporally stable and associated with gene promoter regions, and derived—location determined by e.g. the *PRDM9* DNA-binding motif and rapidly evolving [145]. Not all species studied have obvious recombination hotspots and considerable progress has also been made in determining the mechanisms governing recombination in these cases and outside hotspots. In *C. elegans,* histone modifications do not strongly associate with recombination [146]; however, other post-translational modifications have been identified; phosphorylation of *REC-1* has been shown to govern CO distribution in *C. elegans* [147].

## 4. Evolutionary processes governing variation in recombination rate

Recombination frequency is a heritable trait, which can be controlled by a few genes (oligogenic) (e.g. [16,18,59,148]) and/or by many genes (polygenic) [20,117], and it can respond to selection [21,22,149]. Selection on recombination can be direct and indirect: it can act directly on variation in recombination when recombination influences gamete viability or fitness (direct consequence in offspring), and indirectly when recombination alters haplotype frequencies and increases selection efficacy (variation-and-selection models) [6,60,150]. With a growing understanding of the genes and molecular mechanisms determining variation in recombination frequency and landscape, and data accumulating in a greater range of organisms, we are in a good position to begin to address long-standing questions about how recombination evolves and how variation in recombination frequency or landscape influences evolutionary processes such as adaptation and speciation. In this section, we begin by exploring the evidence for indirect and direct selection on genome-wide recombination; we then discuss how selection acts to modify recombination in specific regions of the genome and how this influences local adaptation and speciation, and finish with discussion of the evolutionary explanations of the evolution of sex differences in recombination rate.

### (a) Indirect selection on variation in genome-wide recombination rate

Indirect selection on recombination rate has received much empirical and theoretical consideration in order to understand the evolution of sex, but there has been less focus on understanding the processes that govern recombination variation in obligate sexuals (see [60]). Models of the evolution of sex suggest that one of the main advantages of recombination is that it can increase the efficacy of selection and facilitate adaptation (see [3,66,151,152]). It does this by reducing the amount that genetic variants or alleles interfere with each other's response to selection. Alleles can interfere in at least two ways: first, when the presence of one allele alters the fitness effects of another allele (epistasis); and second, when the probability of two alleles at two different loci occurring together in a population is non-random (referred to as linkage disequilibrium (LD)), which can be due to their physical proximity on a chromosome (genetically linked) or because of selection, migration or drift (see [153]). For simplicity, we will use the more general term *allelic non-independence* to refer to LD, epistasis and other processes that make alleles behave non-independently. Allelic *non-independence* can interfere with how an allele responds to selection. For example, selection at one locus interferes with selection at other selected loci, reducing its probability of fixation (termed Hill–Robertson interference (HRI) [152], and the degree of interference increases with genetic linkage between the loci under selection. Another example is when alleles in LD experience conflicting selection pressures—if a beneficial allele is associated with a strongly deleterious allele, it can be lost from the population, whereas a deleterious allele can rise to high frequency if it is associated with a strongly beneficial allele. Finally, selection at one locus can reduce the level of polymorphism at linked loci (an effect called background selection when purifying selection acts on a deleterious allele and selective sweep when positive selection acts on a beneficial allele), and this selection at linked sites was found to be a key factor determining genetic diversity within a species and diversity within the genome across animals and plants [154]. The most recognized benefits of recombination in sexual species is that (i) it can increase the efficacy of selection by modifying the degree of independence among alleles: it can break down negative LD generated by selection and drift, thus reducing HRI; and (ii) it can create beneficial combinations of alleles and create greater genetic variation that selection can act on. What makes recombination paradoxical is that it can break apart combinations of beneficial alleles that selection has brought together, resulting in negative fitness effects, both direct [2] and indirect [4,26]. Therefore, the benefits of recombination are dependent on how alleles are associated and how breaking up these associations influences fitness.

Several demographic and ecological factors can increase the number and strength of allelic non-independence within a population. For example, small effective population size (*N*_e_) and high rates of inbreeding or selfing will increase associations between alleles and thus HRI; in these cases, indirect selection should favour an increase in the rate of recombination [66,155]. In line with this expectation, studies have found a negative association between recombination rate and indirect measures of *N*_e_ across species of animals and plants. In mammals, Cf/B was positively correlated with age at maturity, with greater age a proxy for smaller *N*_e_ [156], and in snails, it was negatively correlated with population density [78]. In plants, recombination (cM/Mb) was higher in large, long-lived tree species compared to shrubs and herbs [25]; Cf/B was higher in selfing plants [157] and higher in annual plants that are probably experiencing higher rates of inbreeding and drift [74]. Higher rates of asexual reproduction, for example in parthenogenetic animals or fungi, would also increase HRI and should also select for higher rates of recombination. In line with this prediction, we observed elevated recombination in parthenogenic animals compared to animals with gonochorus sexual systems—where all individuals are either male or female and reproduce sexually every generation (electronic supplementary material, figure S4). Taken together, these data suggest that optimal rates of recombination between species have evolved to reduce HRI and increase genetic variation and the efficacy of selection; however, these relationships do not provide definitive proof of causality. For example, in mammals longer-lived species have a longer meiotic arrest in females, which may favour higher recombination to prevent aneuploidy [158].

Increased recombination can also evolve in populations experiencing strong directional selection and drift [152], even when traits unrelated to meiosis or recombination are being selected for (e.g. [159,160]). This may explain observations of increased recombination in some domesticated species [156,161]. However, there is mixed evidence for changes in overall recombination rates between artificially selected populations and their wild progenitors [152]: a study comparing chiasma counts in wild and domesticated mammal species pairs saw no differences [162], suggesting that an increase in recombination is not a universal feature of domestication.

Populations experiencing heterogeneity in selection are also expected to benefit from higher rates of recombination. In particular, higher rates are predicted when organisms experience rapid oscillations in the fitness of certain allelic combinations, for example in organisms involved in a coevolutionary arms race [1], or that experience fluctuating environments [5,163] or inter-locus sexual conflict [164]. In an arms race scenario, parasite-induced selection on the host can drive an increase in recombination rate. This has been confirmed in several experimental evolution studies (see §2e) and supported by indirect evidence: high recombination in genomic regions harbouring genes related to immunity (e.g. major histocompatibility complex (MHC) [165], *Arabidopsis* [166]) and high somatic recombination observed in developing lymphocytes in jawed vertebrates [167]. Studies testing this model normally consider parasite-induced changes in the host; however, it is possible that host-induced selection on the parasite can also drive a high recombination rate in parasites [168]. We tested this hypothesis with our data by comparing the GwRR of parasitic or pathogenic species with free-living species. Using phylogenetic generalized linear models, we found that parasitic or pathogenic species had a higher recombination rate compared to their free-living counterparts in SAR and in animals, but there was no difference between parasitic or pathogenic and free-living species of fungi (electronic supplementary material, figure S5; plants were excluded as data were not available for any parasitic or pathogenic plant species). Interestingly, parasites often have smaller genomes compared to their free-living counterparts, which is consistent with high recombination driving genome contraction (discussed earlier in box 2), although genome contraction may also be due to selection on small cell size and fast replication rates [55,169].

Spatial and temporal variation in the abiotic environment can also favour higher recombination [5,153,163], although there is little evidence testing this hypothesis in sexual species (studies more often compare between sexual and asexual populations). Temporal variation is often considered less likely to drive increases in recombination because the fluctuations in the abiotic environment are not fast or predictable enough (see [164]). Data collected in the field investigating the effects of spatial variation in an abiotic environment on recombination often cannot rule out other confounding effects such as demography or biotic factors. For example, marginal populations of *Drosophila robusta*, which can experience greater environmental fluctuations, have fewer inversion heterozygotes and thus higher recombination rates (see [5]). In plants, higher Cf/B was found in annuals that are well suited to colonizing new variable habitats [74]. However, in both cases higher recombination rate may be favoured because of the small *N*_e_ of marginal or colonizing populations. More empirical work is needed to test this hypothesis, ideally comparing across natural populations while controlling for potential confounding effects.

Theoretically, differential selection pressure on males and females can induce fluctuating selection on an allele as it cycles through the male and female genomes [164]. Differential selection on male and female traits, such as mating rate or parental investment, creates intra-locus sexual conflict that could favour increased recombination [164]. One prediction that can be drawn from this model is that hermaphrodites, which do not have separate sexes and thus have low levels of intra-locus sexual conflict, should have lower recombination rates compared to species with separate sexes. We tested this in our data looking at how sexual system (gonochorous, hermaphrodite, male-haploid and parthenogenic) was related to recombination rate (GwRR/HCN) across animals. We found that parthenogenic and male-haploid species had a higher recombination rate compared to species with separate sexes, but found no difference between separate sexes and hermaphrodites (see the electronic supplementary material). The dataset used here has a limited number of hermaphrodites (*n* = 7) and it will be interesting to explore this question and other questions relating to the strength of sexual selection with more data.

### (b) Direct selection on variation in genome-wide recombination rate

Considering direct selection on recombination, ensuring proper chromosome segregation and efficient DNA repair imposes stabilizing selection on recombination, thus creating an ‘optimal range’ for a given organism. Extremely high or low rates of recombination outside this optimal range can have negative effects on fitness; for example, in humans and mice very low recombination rates can cause chromosomal abnormalities in gametes and reduce fertility, and very high rates can cause genomic instability and disease [170]. As discussed, obligate CO requirements and genomic architecture can explain some, but not all, of the variation observed between species in the optimal range of the GwRR (box 2). Changes in the environment can push recombination beyond the optimal range with negative fitness consequences, and tolerance to these perturbations may explain some of the variation between species [109].

Considering less extreme modifications of recombination (within the optimal range), there are a few studies linking GwRR to fitness, but there is no clear directional pattern. In populations at equilibrium, recombination is expected to reduce fitness because it breaks apart allelic combinations that selection has favoured (termed recombination load) [2], and several studies in *Drosophila* support this prediction (e.g. [2,171,172]). In humans, a positive relationship between GwRR (cM/Mb) and female fecundity was found, which was argued to be due to a higher number of COs reducing the frequency of age-related non-disjunction, and increasing the likelihood that the gamete became a live birth (realized recombination) [148]. In flour beetles (*Tribolium castaneum*), lines that evolved longer linkage map lengths (i.e. higher GwRR) during coevolution with their parasite were found to have higher fitness in the absence of the parasite compared to lines with shorter linkage maps [117]. The authors did not identify any possible explanations, but posited that it may be due to coevolution with the parasite selecting for fitter beetles. Although studies at the genome-wide level provide evidence of correlations, they may not be very informative with respect to the mechanisms underlying any fitness–recombination relationships. Studies that can quantify where in the genome recombination is modified, not just the change in the overall rate, are likely to provide more insight into the traits that are involved and how changes in recombination influence these.

### (c) Selection on recombination rate modification in regions of the genome

In comparison to the genome-wide scale, there is good evidence that selection acts to reduce recombination on specific chromosomes (i.e. sex chromosomes) and smaller regions of the genome capturing co-adapted loci, QTLs and reproductive isolating loci (i.e. inversions, supergenes). Recombination between these sets of co-adapted loci can negatively affect offspring fitness and adaptation, and strong selection against recombination in these regions is expected to outweigh relatively weak selection for increased recombination to reduce HRI [153]. Processes leading to tight physical linkage can reduce effective recombination between sets of adaptive and reproductive isolating loci, therefore playing a key role in adaptation and speciation [8,173], and can be selected for under prolonged periods of gene flow between locally adapted or diverging populations [125,153,174]. Regions of tight linkage can evolve as a consequence of several, non-exclusive mechanisms including: genomic rearrangements (translocations, inversions, TEs or duplications [8,174]), supergenes (i.e. a group of tightly linked loci that regulate a phenotype [175]) and an establishment bias where linkage with an already diverged locus can favour the establishment of new advantageous mutations nearby [174,176]. An increasing number of empirical studies find evidence for concentrated regions of adaptive and reproductive isolating loci (supergenes, tight linkage) or their presence in regions of reduced recombination (e.g. sex chromosomes, inversions), as well as evidence for a negative correlation between recombination rate and genetic differentiation (table 2 provides a non-exhaustive list of recent examples).

**Table 2.** Summary of selected studies demonstrating a link between regional suppression of recombination and adaptation and/or speciation. Details include study species, the main finding and the methods used to identify regions of suppressed recombination (CG, cytogenetic; LM, linkage mapping; LD, LD-based estimate of recombination rate and others). Studies are grouped according to the nature of the relationship between recombination suppression and either adaptive and/or reproductive isolating (RI) traits or genetic differentiation (GD). SNP, single nucleotide polymorphism.

study system	main finding	CG	LM	LD	other	ref.
*(a) adaptive and RI traits map to recombination coldspots*						
inversion clines related to local adaptation						
fruit fly (*Drosophila melanogaster*)	latitudinal cline in inversion, which has shifted with climate change				X	[177]
mosquito (*Anopheles gambiae*)	GD pronounced at inversion breakpoints across an aridity cline	X				[178]
seaweed fly (*Coelopa frigida*)	demonstrating local adaptation of the inversion along a tidal cline	X				[179]
inversions capture adaptive and/or RI traits						
humans *(Homo sapiens*)	inversion shows molecular signatures of positive selection and is associated with higher fitness				X	[180]
butterfly (*Heliconius numata*)	supergene for mimicry traits is associated with chromosomal rearrangements			X	X	[181]
threespine stickleback (*Gasterosteus aculeatus*)	elevated GD and adaptive loci associated with inversions				X	[182]
Atlantic cod (*Gadus morhua*)	putative inversion association with salinity tolerance			X		[183]
monkey flower (*Mimulus guttatus*)	inversion with adaptive QTLs is the most divergent region between annual and perennial ecotypes		X			[184]
European corn borer moth (*Ostrinia nubilalis*)	inversion contributed to accumulation of ecologically adaptive alleles and GD		X			[185]
Drummond's rockcress (*Boechera stricta*)	inversions captured multiple adaptive QTLs for phenology	X	X	X		[186]
sex chromosomes						
threespine stickleback (*G. aculeatus*)	loci for behavioural isolation and hybrid male sterility map to ancestral and neo X chromosome		X			[187]
house mouse (*Mus musculus / domesticus*)	recombination modifier (*Hstx2*/*Meir1*) and hybrid sterility locus (*Hstx2*) genetically linked on X	X				[188]
*(b) increased GD in recombination coldspots*						
involving chromosomal rearrangements						
mosquito (*Anopheles funestus*)	ecotypes segregate for inversion, but GD is low outside the inversion	X				[189]
apple maggot fly (*Rhagoletis pomonella*)	regions inside and near an inversion had higher GD compared to collinear regions further away			X		[190]
fruit fly (*D. pseudoobscura, D persimilis*)	pairwise GD higher in intergenic regions inside and near an inversion	X				[191]
house mouse (*M. m. domesticus*)	increased GD in proximal regions of Robertsonian fusions	X				[192]
monkey flower (*M. guttatus*)	increased GD in inversions, evidence that inversions have been under recent selection		X			[193]
concentrated in or around centromeres						
** **mosquito (*A. gambiae*)	elevated sequence divergence near centromeres				X	[194]
** **princess cichlid fish *(Neolamprologus savoryi*-complex)	introgression increased with distance from the chromosome centre				X	[195]
concentrated in or around sex chromosomes and/or centromeres						
** **rabbits (*Oryctolagus cuniculus algirus, O. c. cuniculus*)	regions of high GD more common on sex chromosomes and near centromeres				X	[196]
** **mosquito (*Anopheles* spp.)	barriers to introgression on X chromosomes and low recombining pericentromeric regions			X	X	[197]
*(c) genome-wide negative correlation of GD and recombination rate*						
genomic differentiation estimated with SNPs from whole-genome sequencing						
** **monkey flower (*Mimulus nasutus/guttatus*)	negative relationship between recombination rate and absolute divergence		X			[198]
** **flycatchers (*Ficedula albicollis, F. hypoleuca, F. speculigera, F. semitorquata*)	differentiation is explained by variation in recombination rate and the density of targets for selection		X			[199]
** **threespine stickleback (*G. aculeatus*)	recombination rates in regions of exceptional differentiation were often reduced			X		[200]
** **crows (*Corvus (corone)* spp)	heterogeneity in GD is explained by linked selection on a shared genome architecture			X		[201]
** **European and American aspens (*Populus tremula, P. tremuloides*)	linked selection generates heterogeneity of differentiation correlated with recombination			X		[202]
** **Darwin finches (*Geospiza, Camarhynchus, Platyspiza, Pinaroloxias* spp)	genomic islands of locally elevated sequence divergence have low recombination rates			X		[203]
genomic differentiation based on SNPs from transcriptome sequence data						
** **sunflowers (*Helianthus annuus, H. petiolaris, H. debilis, H. argophyllus*)	highly differentiated regions are associated with reduced recombination rates		X			[204]
** **house mouse (*M. m. musculus, M. m. domesticus, M. m. castaneus*)	levels of differentiation were generally higher in regions of low recombination		X			[205]
genomic differentiation based on SNPs sampled using SNP-chip, reduced representation libraries						
** **humans (*H. sapiens*)	*F*_ST_ reduced in the portion of the genome with the highest recombination rate		X			[206]
** **threespine stickleback (*G. aculeatus*)	recombination rate correlates with the magnitude of allele frequency shift		X			[207]
** **house mouse (*M. m. musculus, M. m. domesticus*)	reduced introgression and higher genomic differentiation associated with lower rates of recombination					[208]
** **threespine stickleback (*G. aculeatus*)	adaptive alleles occur more often in regions of low recombination in the presence of divergent selection and gene flow		X			[209]

### (d) Evolutionary explanations for sex differences in recombination

The prevailing hypothesis for the complete absence of recombination in the heterogametic sex is that achiasmy is a pleiotropic effect of selection for tight linkage on the Y or W chromosomes and/or suppression of recombination between the heterogametic sex chromosomes [94,96]. However, reduced recombination is not always observed in the heterogametic sex (i.e. birds and moths) and it cannot explain variation between the sexes in hermaphrodites [210]. Understanding the conditions under which heterochiasmy evolves has been the subject of extensive theoretical attention and debate (e.g. [96]), but as yet, there is no consensus on its evolutionary drivers. Arguments related to the relative strengths of sexual selection, sperm competition and dispersal remain weakly supported by empirical data [211], with some arguing that sex differences are primarily driven by drift [94,96]. Nevertheless, there are two arguments gaining broader theoretical and empirical support. The first is haploid selection; the sex experiencing the strongest haploid selection should recombine less (see [89]). In plants, both female and male gametes have a haploid phase, but Lenormand & Dutheil [89] proposed that selfing could be used as a proxy for the strength of selection on the female haploid phase, and showed that the degree of heterochiasmy (male–female ratio) was higher in species with moderate to high selfing. The second is the role of meiotic drive, for example where asymmetry in female meiosis can be exploited by selfish genetic elements associated with centromere strength [90,212,213]; selection for increased recombination at centromeric regions will counteract meiotic drive by increasing the uncertainty of segregation into the egg [212].

## 5. Concluding remarks and future directions

Recombination is a fundamental component of meiosis and a near universal mechanism in multicellular organisms, with far-reaching effects on an individual's fitness and on evolutionary processes. Whole-genome sequencing, dense marker panels and the development of new approaches to estimate population-scaled recombination rates have provided new opportunities to estimate recombination at much greater resolution and across natural populations, with great impact. Genome-wide averages of recombination rate are useful for broad-scale comparisons; however, averaging the number of recombination events across the genome can mask the dynamic nature of changes in distribution at a finer genomic scale. Studies in the future should consider the fine genomic landscape and not only the frequency. Across eukaryotes, there is large variation between taxa, populations and individuals in the frequency and distribution of recombination. In figure 1, we illustrate how variation collected and compared across different taxonomic scales provides complementary information to address many important and outstanding questions about how and why recombination varies.

Significant progress has been made recently in identifying the genetic and epigenetic mechanisms governing the recombination landscape; for example, the presence or absence of one locus in particular (*PRDM9*) can explain variation across species and how conserved or dynamic their recombination landscape is. However, it is unclear how widespread recombination hotspots are, and if all hotspots fall broadly into two categories—conserved versus rapidly evolving, although comparative studies are moving some way to elucidate this issue [88]. Other features of the recombination landscape, such as sex differences and plasticity, are also lacking empirical support across a wide range of taxa. We urge researchers to collect recombination data at the fine genomic scale in a greater range of species, in particular neglected taxa (marine microorganisms, basal animals and plants) and to estimate (and report) both sex-specific and sex-averaged recombination rates. LD-based estimates are likely to be especially powerful in this respect as they provide opportunities to estimate recombination rate from polymorphism data of sampled populations without the need to create crosses or use pedigrees. Data from a greater range of species can further our understanding of the molecular mechanisms underlying recombination and enable us to address a range of long-standing questions regarding the evolution of recombination.

Understanding the fitness consequences and evolutionary processes driving variation in recombination rate is still in its infancy. Investigation of how changes in recombination can directly influence phenotypic traits and fitness is needed and, although established theory on the evolution of sex considers the conditions under which changes in the GwRR may be favoured, there are almost no empirical data testing these predictions in sexual organisms. More comparisons across related taxa, populations and individuals in the field are needed to characterize natural variation in recombination rate. Comparisons across populations and taxa could ask if, for example, drift, fluctuating selection and modes of reproduction covary with variation in recombination. Studying the recombination landscape across an environmental or ecological gradient while controlling for possible confounding effects of drift and changes in *N*_e_ are likely to be most informative. Experimental evolution studies could manipulate population parameters and see if recombination rate evolves in response to changes in density, inbreeding, fluctuating selection and parasites, and could investigate how changes in recombination rate influence fitness-related traits.

More effort should be devoted to modelling recombination rate as a quantitative trait and consider how it will respond to different selection regimes in sexually reproducing organisms (see [60]). Models of the evolution of GwRRs may have limited power to explain variation in the landscape at fine genomic scales. Mathematical models could explore how selection influences patterns of recombination near loci under strong selection or loci involved in coevolutionary arms races, for example. Regional suppression of recombination on specific genomic features (inversions, supergenes) is receiving increased attention in the literature, spurred on by the recognition that the association of these features with suppressed recombination is key to adaptation and speciation in the presence of gene flow. Current empirical challenges reside in determining the sequence of events that have permitted favourable genomic features or recombination modifiers to establish and be maintained in the presence of gene flow, from the selection of pre-existing favourable genomic features to the selection of mechanisms generating them during the course of the processes of adaptation and speciation.

To summarize, there is enormous variation in recombination frequency and landscape across species and genomes. Great progress has been made in determining the genetic and epigenetic factors controlling recombination, but more theoretical and empirical data are needed to further our understanding of why recombination varies and to determine if this variation is the result of selection.

## Supplementary Material

Supplementary Methods and Results

## Supplementary Material

Complete list of species and linkage map data used in analysis

## Supplementary Material

Reference list for linkage map data

## Supplementary Material

Phylogenetic tree used in analysis

## Data accessibility

All additional data are provided in the electronic supplementary material.
