# Supplementary material for: Variation in recombination frequency and distribution across eukaryotes: patterns and processes
Source: Philos Trans R Soc Lond B Biol Sci. 2017 Nov 6;372(1736):20160455. doi: 10.1098/rstb.2016.0455 (PMC5698618; doi:10.1098/rstb.2016.0455)
Supplement: Supplementary Methods and Results [file rstb20160455supp1.pdf]

Supplementary Information

Methods

We obtained linkage map lengths, genome size and haploid chromosome number from published literature and online databases. For linkage maps, we searched Web of Science for all papers containing the terms “linkage map” and “genetic map” from 1998 onwards. This produced a list of over 10,000 references (referred to as WoS10k). We then filtered this list by excluding papers published before 2000 and including only papers with the term “linkage map” in the title. From this list of Linkage Map papers we extracted species names and created a list of species to focus on. This species list was complemented with additional species from previous studies (Ross-Ibarra 2007, Corbett-Detig et al. 2015, Tiley and Burleigh 2015). Using this species list we then searched our WoS10k library and identified the most comprehensive map for each species, i.e. the map with the greatest number of markers. If marker number was similar between maps, we chose the map with highest number of individuals. For each species we recorded sex-averaged linkage map length and number of linkage groups. In some cases the sex average map length was not reported in the paper, for these cases we took the average of the male and female specific maps. We then added genome size and haploid chromosome number information to this list. This data was extracted from multiple sources, including databases (Genome Size Databases: http://www.genomesize.com/index.php, http://data.kew.org/cvalues/, http://eupathdb.org/eupathdb/, http://www.zbi.ee/fungal-genomesize/index.php) and published literature including the Linkage map papers. From this species list we then excluded low quality maps; we removed maps that had low coverage (<50 makers) and maps where the number of linkage groups (LG) and the haploid chromosome number (HCN) differed markedly (absolute(LG-HCN)/HCN > 0.7). Our final dataset for analysis contained 353 species (see Table S1 for species list and Table S2 for list of references).

In our analysis we controlled for phylogeny by fitting a Phylogenetic Generalized Linear (PGLS) Model with the R Package ‘Caper’ (Orme et al. 2013). The phylogeny was obtained using Phylotastic Web Service (https://github.com/phylotastic/phylo_services_docs/blob/master/ServiceDescription/PhyloServicesDescription.md), which extracts a Supertree from openTree (Hinchliff et al. 2015). The branch lengths were all set to 1 in the Supertree.

We considered linear and quadratic effects in PGLS models. We considered patterns across all groups (all), and also considered the relationship across each of the three major groups (Fungi, Animals and Plants) separately. Relationships in SAR were not analysed because n<10. All analyses and plots were done with R (v3.3.2) (Ihaka and Gentleman 1996).

Example code for R

```
library(Hmisc)
library(caper)
library(ape)
library(picante)
```

'tree' is the phylogenetic tree in Supplementary Material

'data' is Table S1.

Make tree binary and make node labels

```
tree = multi2di(tree)
tree = makeNodeLabel(tree)
```

To see what species are in the tree but not in the data in column 'speciesOTT'

```
setdiff(tree$tip.label, data$speciesOTT)
```

To see what species are in the data in column 'speciesOTT' but not in the tree.

```
setdiff(data$speciesOTT, tree$tip.label)
```

Six species are in our data but not in the tree: *Astyanax mexicanus, Bactrocera cucurbitae, Brassica rapa oleifera, Capsella bursa pastoris, Fusarium verticilliodes, Taraxacum kok saghyz*

Combine phylogenetic tree and data

```
treedat = comparative.data(phy= tree, data= data, names.col="speciesOTT", vcv=TRUE,
 na.omit=FALSE, warn.drop=TRUE)
```

For analysis of taxonomic groups separately we subsetted the data.

For example

```
Plants = droplevels(subset(data, data$Group=="Plants"))
treePlant = comparative.data(phy= tree, data= Plants, names.col="speciesOTT", vcv=TRUE,
 na.omit=FALSE, warn.drop=TRUE)
```

Example model to investigate the relationship between log map length (lLM) and log haploid chromosome number (lHCN).

```
pgls.lLM.lHCN =pgls(lLM ~lHCN, treedat, lambda="ML")
```

For quadratic term

```
pgls.lLM.lHCN =pgls(lLM ~poly(lHCN,2), treedat, lambda="ML")
```

Supplementary Results

Linkage map length and genome size

Log Linkage map length (cM) was positively correlated with the log genome size (Mb) across the major taxonomic groups ($F_{(1,341)}$ = 14.78, *p* < 0.001). When considering the major taxonomic groups separately we found a positive linear relationship for Animals (Linear Term: est = 4.08, se = 0.97, *t* = 4.19, *p* < 0.001, Quadratic Term: est = -0.04, se = 0.68, *t* = -0.59, *p* = 0.52) and Fungi (Linear Term: est = 0.99, se = 0.42, *t* = 2.32, *p* = 0.04, Quadratic Term: est = 0.62, se = 0.46, *t* = 1.35, *p* = 0.20) and a significant quadratic term for Plants (Linear Term: est = 1.19, se = 0.76, *t* = 1.55, *p* = 0.12, Quadratic Term: est = -1.41, se = 0.65, *t* = -2.17, *p* = 0.03). The slopes of the linear relationships for Animals and Fungi were both less than one (0.32±0.07, 0.61±0.24 respectively).

Linkage map length and haploid chromosome number

Log linkage map length (cM) was positively correlated with haploid chromosome number across the major taxonomic groups ($F_{(1,345)}$ = 107.1, *p* < 0.001). When considering the major taxonomic groups separately we found a positive linear relationship for Plants (Linear Term: est = 0.83, se = 0.07, *t* = 11.43, *p* < 0.001, Quadratic Term: est = -0.22, se = 0.41, *t* = -0.55, *p*=0.58) and Fungi (Linear Term: est = 0.74, se = 0.3, *t* = 2.18, *p* = 0.05, Quadratic Term: est = 0.42, se = 0.47, *t* = 0.88, *p* = 0.39) and a significant quadratic term for Animals (Linear Term: est = 0.91, se = 0.08, *t* = 10.50, *p* < 0.001, Quadratic Term: est = 1.18, se = 0.59, *t* = -2.00, *p* = 0.04).

Genome-wide recombination rate and haploid chromosome number

Comparing across all Eukaryotes we found no relationship between log haploid chromosome number and log recombination rate (cM/Mb) ($F_{(1,341)}$ = 2.97, *p* = 0.08). When we consider each group separately we found no relationship for Fungi (Linear term est = 0.30, se = 0.51, *t* = 0.58, *p* = 0.57, Quadratic term est = -0.18, se = 0.55, *t* = -0.32, *p* = 0.75) or Animals (Linear Term: est = 1.37, se = 1.27, *t* = 1.047, *p* = 0.28, Quadratic Term: est = -0.42, se = 1.10, *t* = -0.38, *p* = 0.70), but we identified a significant linear relationship in Plants (Linear Term: est = 2.08, se = 0.85, *t* = 2.43, *p* = 0.01, Quadratic Term: est = 0.44, se = 0.83, *t* = 0.53, *p* = 0.59).

Relationship between sexual system and recombination rate

We investigated if sexual system explained variation in log (GwRR/HCN) in Animals. We did not investigate patterns in Plants because we do not have complete information on the sexual system. Using phylogenetic generalized linear models (PGLS) we found that log (GwRR/HCN) was higher in parthenogenic and male-haploid species compared to species of Animals where each individual is either male or female (Figure S4, $F_{(1,104)}$ = 8.05, *p* < 0.001, estimated difference between Gonochorous and hermaphrodite = 0.56, se = 0.35, *t* = 1.59, *p* = 0.11; estimated difference between Gonochorous and male.haploid = 1.29, se = 0.65, *t* = 1.99, *p* = 0.04; estimated difference between Gonochorous and parthenogenic = 1.73, se = 0.39, *t* = 4.44, *p* < 0.001; Samples size were: n(Gonochorous)=89, n(Hermaphrodite)=7, n(male.haploid)=9, n(parthenogenic)=5).

Differences between parasitic/pathogenic species and free living species

We compared the GwRR/HCN between free living and parasitic/pathogenic species of Fungi, SAR and Animals. We excluded Plants because we do not have any parasitic/pathogenic plant species in our dataset. Using phylogenetic generalized linear models (PGLS) we found that log (GwRR/HCN) was higher in parasitic or pathogenic species of Animals (Figure S5, $F_{(1,135)}$ = 7.42, *p* = 0.007, Linear Term: est = 1.05, se = 0.38, *t* = 2.72, *p* = 0.007, n(par/path)=7, n(free living)=133) and SAR ($F_{(1,7)}$ = 16.27, *p* = 0.004, Linear Term: est = 3.05, se = 0.75, *t* = 4.03, *p* = 0.004, n(par/path)=6, n(free living)=3), but not different in Fungi ($F_{(1,11)}$ = 1.08, *p* = 0.31, Linear Term: est = -0.48, se = 0.46, *t* = -1.05, *p* = 0.31, n(par/path)=11, n(free living)=4).

Supplementary References

Corbett-Detig, R. B., D. L. Hartl, and T. B. Sackton. 2015. Natural selection constrains neutral diversity across a wide range of species. PLoS Biol **13**:e1002112.

Hinchliff, C. E., S. A. Smith, J. F. Allman, J. G. Burleigh, R. Chaudhary, L. M. Coghill, K. A. Crandall, J. Deng, B. T. Drew, R. Gazis, K. Gude, D. S. Hibbett, L. A. Katz, H. D. Laughinghouse, E. J. McTavish, P. E. Midford, C. L. Owen, R. H. Ree, J. A. Rees, D. E. Soltis, T. Williams, and K. A. Cranston. 2015. Synthesis of phylogeny and taxonomy into a comprehensive tree of life. Proceedings of the National Academy of Sciences **112**:12764-12769.

Ihaka, R., and R. Gentleman. 1996. R: A language for data analysis and graphics. Journal of Computational and Graphical Statistics **5**:299-314.

Orme, C. D. L., R. P. Freckleton, G. H. Thomas, T. Petzoldt, S. A. Fritz, and N. J. B. Isaac. 2013. CAPER: comparative analyses of phylogenetics and evolution in R.

Ross-Ibarra, J. 2007. Genome size and recombination in angiosperms: a second look. Journal of Evolutionary Biology **20**:800-806.

Tiley, G. P., and G. Burleigh. 2015. The relationship of recombination rate, genome structure, and patterns of molecular evolution across angiosperms. BMC Evolutionary Biology **15**:194.

Supplementary Tables (attached pdfs)

Table S1. Linkage Map data.

Table S2. Corresponding Reference list to the Linkage Map data.

Supplementary Figures

Figure S1. Log Recombination rate across major non-vertebrate, Fungi and SAR Families. Families with only one species were grouped (Other Fungi: Pleosporales, Eurotiales, Tremellales, Magnaporthales, Uredinales, Pleosporales, Capnodiales; Other SAR: Ectocarpales, Peronosporales, Tyranosomatida; Other Helminth: 3x Tylenchida, Strigeidida; Other Crustacean: Anostraca, Harpticoida; Other Mollusc: Unionida, Venerida; Other Invert: Scleractinia, Aspidochirotida, Ixodida)

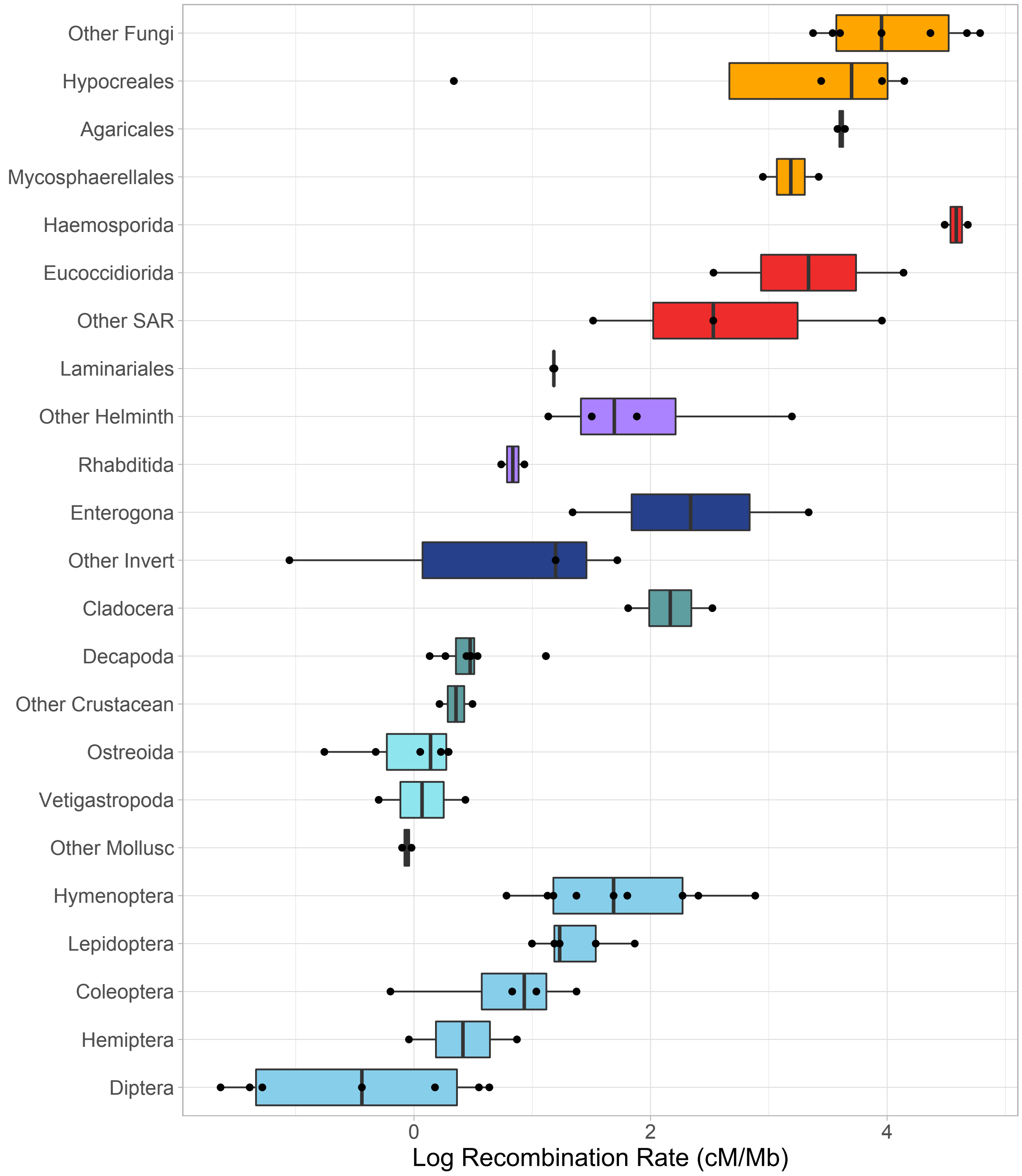

Figure S2. Log Recombination rate across major vertebrate Families. Families with only one species were grouped (Other Fishes: Anguilliformes, Scorpaeniformes, Characiformes, Esociformes, Gadiformes, Gasterosteiformes, Siluriformes, Cyprinodontiformes, Osteoglossiformes, Tetraodontiformes; Other Mammal: Perissodactyla, Diprotodontia, Didelphimorphia, Lagomorpha; Amphibian + Reptile: Caudata, Crocodilia).

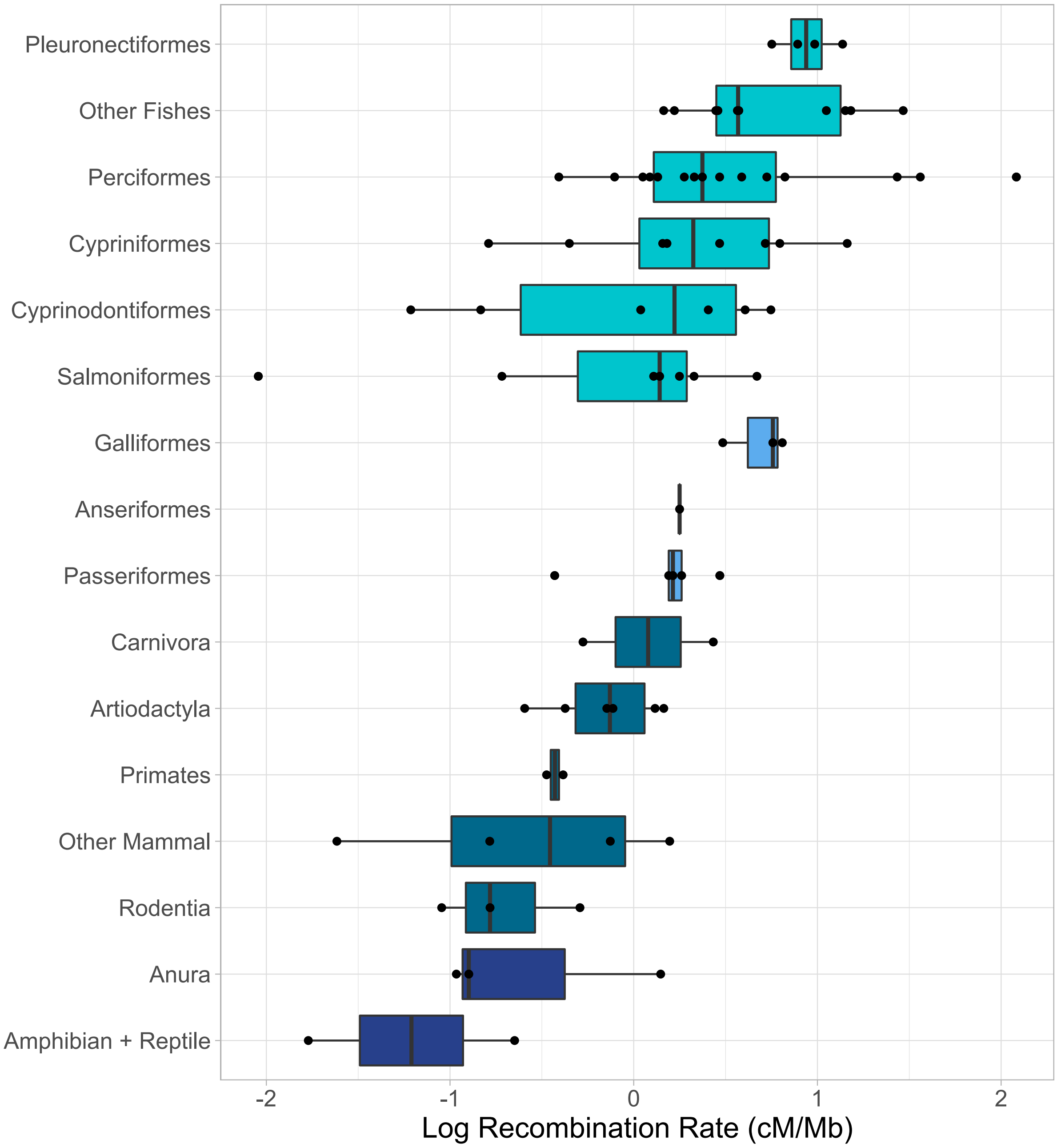

Figure S3. Log Recombination rate across major plant Families. Families with only one species were grouped (Other Plants: Polypodiales, Chlamydomoadales, Funariales; Other Monocots: Alismatales, Zingiberales; Other Eudicots: Cucurbitales, Cornales, Apiales, Garryales).

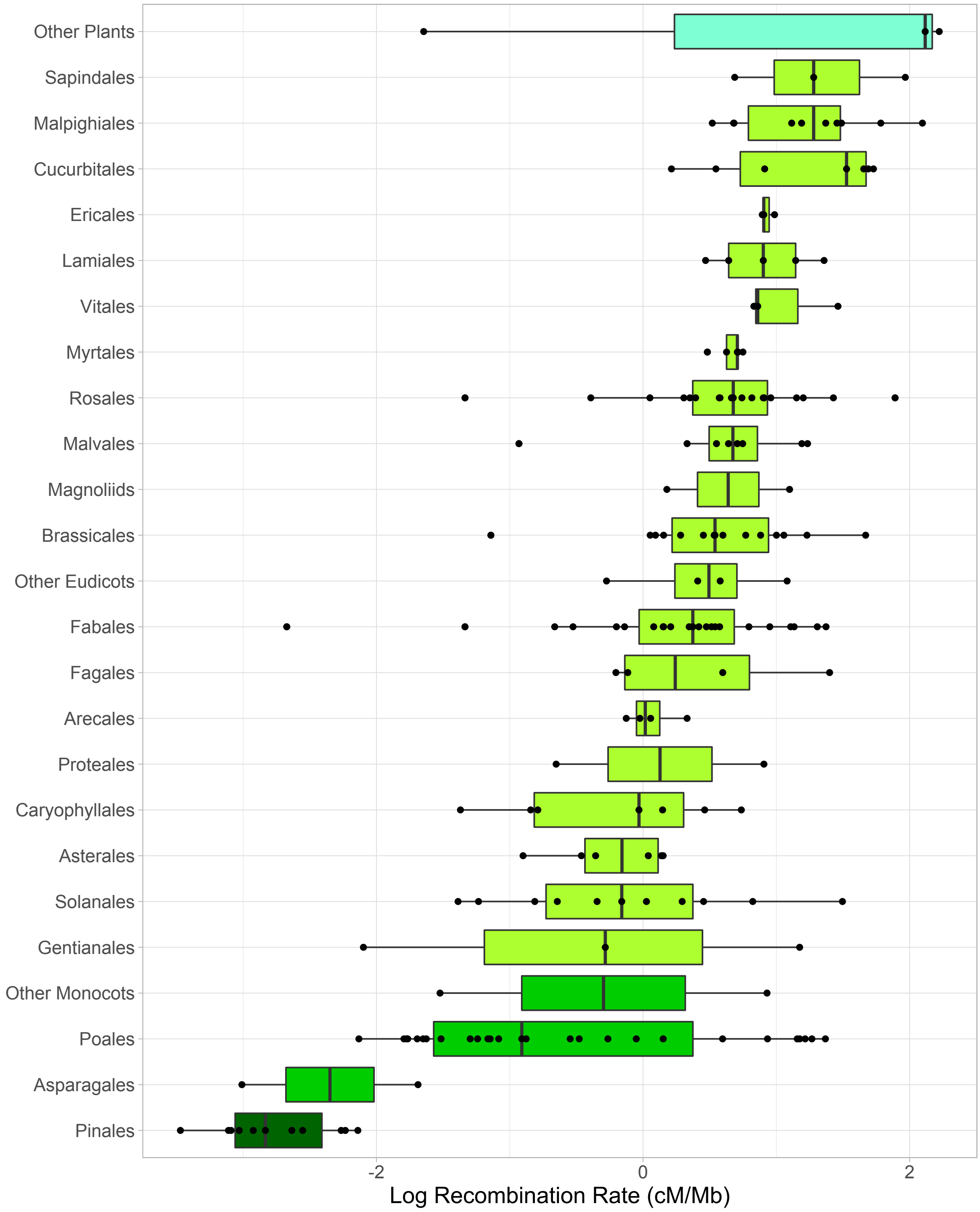

Figure S4. Log of Recombination rate (cM/Mb) divided by haploid chromosome number (HCN) in relation to reproductive system in Animals (gonochorous – individuals belong to separate sexes, hermaphrodite – individual have both sexes, male.haploid – haploid/diploid reproductive system, parthenogenetic– combination of sexual and asexual/clonal reproduction). Solid points are actual data points, shaded areas are boxplots.

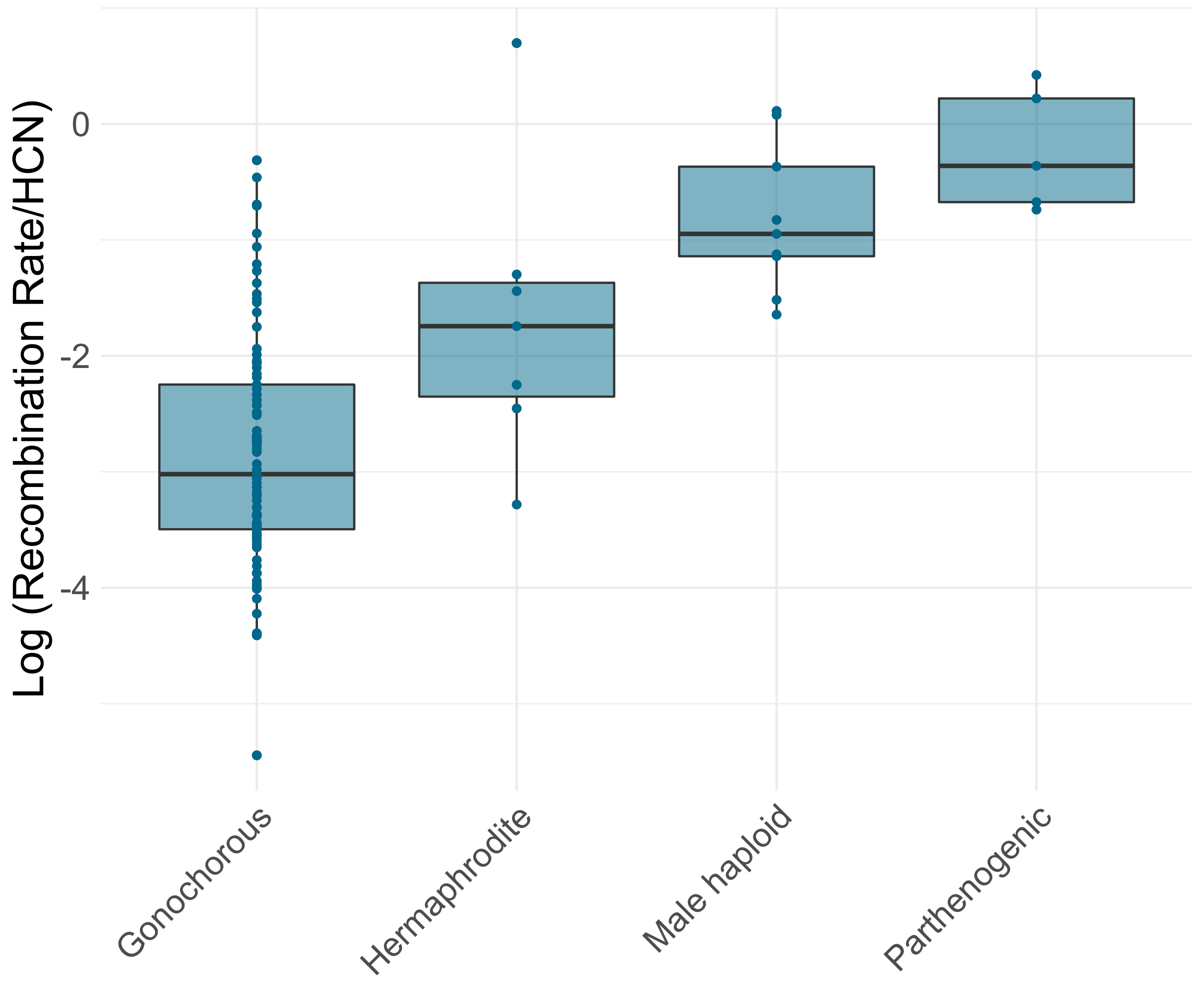

Figure S5. Log of Recombination rate (cM/Mb) divided by haploid chromosome number (HCN) in relation to life form – parasitic or parthenogenetic versus free-living across species of SAR (red), Fungi (Orange) and Animals (Blue). Solid points are actual data points, shaded areas are boxplots.

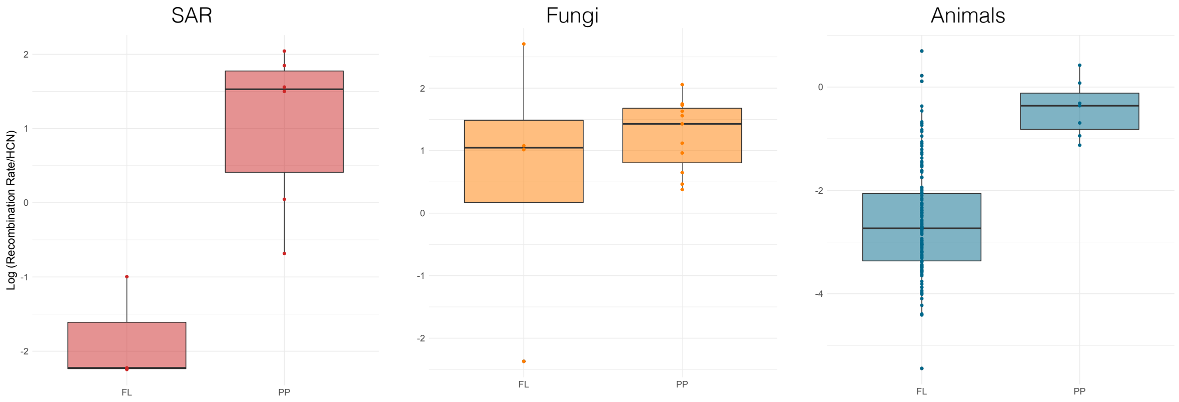

Life form: FL = Free living, PP=Parasitic or Pathogenic
