# Supplementary material for: Variation in recombination frequency and distribution across eukaryotes: patterns and processes
Source: Philos Trans R Soc Lond B Biol Sci. 2017 Nov 6;372(1736):20160455. doi: 10.1098/rstb.2016.0455 (PMC5698618; doi:10.1098/rstb.2016.0455)
Supplement: Complete list of species and linkage map data used in analysis [file rstb20160455supp2.pdf]

Ref	year	species
Butcher	2000	Acacia_mangium
Sirvio	2011	Acromyrmex_echinatior
Wang	2009	Acropora_millepora
Scaglione	2015	Actinidia_chinensis
Hawthorne	2001	Acyrthosiphon_pisum
Juneja	2014	Aedes_aegypti
Sutherland	2011	Aedes_albopictus
Olivera	2013	Aegilops_sharonensis
Luo	2013	Aegilops_tauschii
Edae	2017	Aegilops_umbellulata
Foulogne-Ori	2010	Agaricus_bisporus
Rotter	2009	Agrostis_capillaris
Honig	2014	Agrostis_stolonifera
Baldwin	2012	Allium_cepa
Maughan	2011	Amaranthus_hybridus
Smith	2005	Ambystoma_tigrinum
deSousa	2013	Ananas_comosus
Huang	2009	Anas_platyrhynchos
Kai	2014	Anguilla_japonica
Wondji	2007	Anopheles_funestus
Rondeau	2013	Anoplopoma_fimbria
Venkat	2014	Anthurium_andreanum
Shi	2013	Apis_cerana
Wilfert	2007	Apis_mellifera
Tian	2015	Apostichopus_japonicus
Willems	2007	Arabidopsis_halleri
Singer	2015	Arabidopsis_thaliana
Nagy	2010	Arachis_duranensis
Zhou	2014	Arachis_hypogaea
Li	2012	Argopecten_irradians
Zhu	2014	Aristichthys_nobilis
DeVos	2013	Artemia_franciscana
Phan	2003	Ascochyta_rabiei
Christians	2011	Aspergillus_nidulans
Sanetra	2009	Astatotilapia_burtoni
Carlson	2015	Astyanax_mexicanus
Chafffin	2016	Avena_sativa
Sim	2017	Bactrocera_cucurbitae
Kuzina	2011	Barbarea_vulgaris
Brennan	2012	Begonia_plebeja
Laurent	2007	Beta_vulgaris
Jiang	2011	Betula_pendula
Beldade	2009	Bicyclus_anynana
Schnabel	2003	Bison_bison

VantHof	2013	Biston_betularia
Liu	2014	Boehmeria_nivea
Stolle	2011	Bombus_terrestris
Zhan	2009	Bombyx_mori
Ihara	2004	Bos_taurus
Holloway	2001	Bracon_hebetor
Cheng	2009	Brassica_campestris
Zou	2014	Brassica_carinata
Zou	2016	Brassica_juncea
Zhang	2016	Brassica_napus
Zhao	2016	Brassica_oleracea
Yu	2016	Brassica_rapa
Bohra	2012	Cajanus_cajan
Gehringer	2006	Camelina_sativa
Taniguchi	2012	Camellia_sinensis
Wong	2010	Canis_lupus
Schibler	1998	Capra_hircus
Linde	2001	Capsella_bursa-pastoris
Han	2016	Capsicum_annuum
Lee	2016	Capsicum_baccatum
Kuang	2016	Carassius_auratus
Chen	2007	Carica_papaya
Bowers	2016	Carthamus_tinctorius
Kubisiak	2013	Castanea_mollissima
Sharma	2011	Catharanthus_roseus
Nakazato	2006	Ceratopteris_richardii
Maughan	2012	Chenopodium_quinoa
Chen	2010	Chimonanthus_praecox
Kathir	2003	Chlamydomonas_reinhardtii
Zhan	2009	Chlamys_farreri
Gaur	2015	Cicer_arietinum
Kano	2007	Ciona_intestinalis
Hill	2008	Ciona_savignyi
Reddy	2014	Citrullus_lanatus
Ollitrault	2012	Citrus_clementina
Guo	2015	Citrus_sinensis
Kaiser	2012	Clunio_marinus
Riedel	2009	Cocos_nucifera
Moncada	2016	Coffea_arabica
Muraguchi	2003	Coprinus_cinereus
Biswass	2015	Corchorus_capsularis
Kundu	2015	Corchorus_olitorius
Gagnaire	2013	Coregonus_clupeaformis
Wang	2009	Cornus_florida
Kikuchi	2005	Coturnix_japonica

Hedgecock	2015	Crassostrea_gigas
Yu	2003	Crassostrea_virginica
Miles	2009	Crocodylus_porosus
Marra	2004	Cryptococcus_neoformans
Moriguchi	2016	Cryptomeria_japonica
Xia	2010	Ctenopharyngodon_idella
Diaz	2011	Cucumis_melo
Zhu	2016	Cucumis_sativus
Zhang	2015	Cucurbita_maxima
Gong	2008	Cucurbita_moschata
Montero-Pau	2017	Cucurbita_pepo
Hickner	2013	Culex_pipiens
Hansson	2010	Cyanistes_caeruleus
Portis	2009	Cynara_cardunculus
Harris-Shultz	2010	Cynodon_dactylon
Song	2012	Cynoglossus_semilaevis
Zhao	2013	Cyprinus_carpio
Zhao	2016	Dactylis_glomerata
Bradley	2011	Danio_rerio
Dukic	2016	Daphnia_magna
Cristescu	2006	Daphnia_pulex
Cavagnaro	2014	Daucus_carota
Yagi	2013	Dianthus_caryophyllus
Chistiakov	2008	Dicentrarchus_labrax
Stocker	2012	Drosophila_serrata
Heesch	2010	Ectocarpus_siliculosus
Blake	2011	Eimeria_maxima
Shirley	2000	Eimeria_tenella
Ting	2016	Elaeis_guineensis
Montoya	2013	Elaeis_oleifera
Mott	2011	Elymus_lanceolatus
Dor	2014	Epinephelus_aeneus
Swinburne	2006	Equus_caballus
Zheng	2013	Eremochloa_ophiuroides
Fukuda	2016	Eriobotrya_japonica
Cui	2015	Eriocheir_sinensis
RONDEAU	2014	Esox_lucius
Agrama	2002	Eucalyptus_camaldulensis
Hudson	2012	Eucalyptus_grandis
Li	2015	Eucalyptus_tereticornis
Li	2015	Eucalyptus_urophylla
Li	2014	Eucommia_ulmoides
Yabe	2014	Fagopyrum_esculentum
Li	2016	Felis_silvestris
Wang	2012	Fenneropenaeus_chinensis

Dierking	2015	Festuca_arundinacea
Backstrom	2008	Ficedula_albicollis
Mahoney	2016	Fragaria_iinumae
Davik	2015	Fragaria_vesca
Sargent	2016	Fragaria_xananassa
DeVos	2007	Fusarium_circinatum
Lee	2008	Fusarium_graminearum
Teunissen	2003	Fusarium_oxysporum
Jurgenson	2002	Fusarium_verticillioides
Hubert	2010	Gadus_morhua
Pengelly	2016	Gallus_gallus
Rastas	2016	Gasterosteus_aculeatus
Nakatsuka	2012	Gentiana_scabra
vanderVoort	1999	Globodera_rostochiensis
Song	2016	Glycine_max
Shi	2016	Gossypium_barbadense
Jia	2016	Gossypium_hirsutum
Wang	2013	Gossypium_raimondii
Hou	2013	Gossypium_tomentosum
Ren	2016	Haliotis_diversicolor
Vervalle	2013	Haliotis_midae
Henning	2017	Haplochromis_chilotes
Henning	2014	Haplochromis_sauvagei
Celik	2016	Helianthus_annuus
Davey	2016	Heliconius_melpomene
Atibalentja	2005	Heterodera_glycines
Shearman	2015	Hevea_brasiliensis
Chen	2011	Hibiscus_cannabinus
Shirasawa	2015	Hieracium_
Palaiokostas	2013	Hippoglossus_hippoglossus
Zhou	2016	Hordeum_vulgare
Henning	2015	Humulus_lupulus
Brelsford	2016	Hyla_aborea
Guo	2013	Hypophthalmichthys_molitrix
Bai	2016	Hyriopsis_cumingii
Liu	2016	Ictalurus_punctatus
Zhao	2013	Ipomoea_batatas
Ullmann	2003	Ixodes_scapularis
Wu	2015	Jatropha_curcas
Zhu	2015	Juglans_regia
Kanomori	2016	Kryptolebias_marmoratus
Robinson	2014	Labeo_rohita
Truco	2013	Lactuca_sativa
Ao	2015	Larimichthys_crocea
Guan	2011	Larix_kaempferi

Wang	2017	Lates_calcarifer
Sudheesh	2016	Lens_culinaris
Wang	2010	Lepomis_macrochirus
Hawthorne	2001	Leptinotarsa_decemlineata
Cloutier	2012	Linum_usitatissimum
Yu	2015	Litopenaeus_vannamei
Guan	2014	Lolium_multiflorum
Velmurugan	2016	Lolium_perenne
Wang	2008	Lotus_japonicus
Berdan	2014	Lucania_goodei
Berdan	2014	Lucania_parva
Wu	2016	Luffa_acutangula
Vipin	2013	Lupinus_albus
Yang	2013	Lupinus_angustifolius
Rogers	2006	Macaca_mulatta
Wang	2011	Macropus_eugenii
Zheng	2008	Magnaporthe_grisea
DiPierro	2016	Malus_domestica
Liu	2016	Malus_sieversii
Clark	2014	Malus_pumila
Luo	2016	Mangifera_indica
Alaba	2015	Manihot_esculenta
Lu	2016	Marsupenaeus_japonicus
Li	2014	Medicago_sativa
Gorton	2012	Medicago_truncatula
Anderson	2016	Melampsora_lini
Aslam	2010	Meleagris_gallopavo
Thomas	2012	Meloidogyne_hapla
Nietlisbach	2015	Melospiza_melodia
O'Quin	2013	Metriaclima_zebra
McGraw	2011	Microtus_ochrogaster
Holeski	2014	Mimulus_guttatus
Liu	2016	Miscanthus_sinensis
Morishima	2008	Misgurnus_anguillicaudatus
Samollow	2007	Monodelphis_domestica
Hipplyte	2010	Musa_acuminata
Kema	2002	Mycosphaerella_graminicola
Diao	2016	Nasonia_giraulti
Zhang	2014	Nelumbo_lutea
Liu	2016	Nelumbo_nucifera
Zhang	2012	Nicotiana_langsdorffii
Gong	2016	Nicotiana_tabacum
Jairin	2013	Nilaparvata_lugens
Kirschner	2012	Nothobranchius_furzeri
Ipek	2016	Olea_europaea

McClelland	2008	Oncorhynchus_kisutch
Guyomard	2012	Oncorhynchus_mykiss
Larson	2016	Oncorhynchus_nerka
McKinney	2016	Oncorhynchus_tshawytscha
Liu	2013	Oreochromis_mossambicus
Kocher	1998	Oreochromis_niloticus
Sternstein	2015	Oryctolagus_cuniculus
Luo	2016	Oryza_rufipogon
DeLeon	2016	Oryza_sativa
Harrang	2015	Ostrea_edulis
Johnston	2017	Ovis_aries
Poissant	2010	Ovis_canadensis
Liu	2012	Panicum_virgatum
Winter	2010	Papilio_glaucus
Rogers	2006	Papio_hamadryas
Song	2012	Paralichthys_olivaceus
vanOers	2014	Parus_major
Wang	2017	Patinopecten_yessoensis
Baranski	2014	Penaeus_monodon
Punnuri	2016	Pennisetum_glaucum
Kenney-Hunt	2014	Peromyscus_maniculatus
Borrone	2009	Persea_americana
Bossolini	2011	Petunia_axillaris
Malkus	2009	Phaeosphaeria_nodorum
Gutierrez	2011	Phalloceros_caudimaculatus
Song	2015	Phaseolus_vulgaris
Mathew	2014	Phoenix_dactylifera
Kamisugi	2008	Physcomitrella_patens
vanderLee	2004	Phytophthora_infestans
Lind	2014	Picea_abies
Friesline	2015	Pinus_balfouriana
Yang	2013	Pinus_elliottii
Chen	2010	Pinus_koraiensis
Jermstad	2011	Pinus_lambertiana
Chen	2014	Pinus_massoniana
Chancerel	2013	Pinus_pinaster
Moraga-Suaz	2014	Pinus_radiata
Westbrook	2015	Pinus_taeda
Boutet	2016	Pisum_sativum
Martinello	2005	Plasmodium_chabaudi
Jiang	2011	Plasmodium_falciparum
Tripathi	2009	Poecilia_reticulata
Sirvio	2011	Pogonomyrmex_rugosus
Wang	2010	Populus_adenopoda
Paolucci	2010	Populus_alba

Mousavi	2016	Populus_deltoides
Gaudet	2008	Populus_nigra
Pakull	2009	Populus_tremula
Liu	2012	Portunus_trituberculatus
Feng	2016	Primulina_eburnea
Srinivasan	2002	Pristionchus_pacificus
Lalli	2008	Prunus_armeniaca
Wang	2015	Prunus_avium
Tavassolian	2010	Prunus_dulcis
Cao	2011	Prunus_kansuensis
Zhang	2015	Prunus_mume
Nunez-Lillo	2015	Prunus_persica
Arango_Isaza	2016	Pseudocercospora_fijiensis
Harel-Beja	2015	Punica_granatum
Wu	2014	Pyrus_communis
Bodenes	2016	Quercus_
Cano	2011	Rana_temporaria
Mun	2015	Raphanus_sativus
Steen	1999	Rattus_norvegicus
Schlipallus	2002	Rhyzopertha_dominica
Rukam	2016	Ricinus_communis
Ward	2013	Rubus_idaeus
Bushakra	2012	Rubus_occidentalis
Nie	2017	Ruditapes_philippinarum
Zhang	2015	Saccharina_japonica
Tsai	2016	Salmo_salar
Leitwein	2017	Salmo_trutta
Liu	2016	Salvia_miltiorrhiza
Criscione	2009	Schistosoma_mansoni
Hollenbeck	2015	Sciaenops_ocellatus
Shen	2014	Scleropages_formosus
Wang	2015	Scophthalmus_maximus
Ma	2016	Scylla_paramamosain
Milczarski	2015	Secale_cereale
Aoki	2015	Seriola_quinqueradiata
Zhang	2016	Sesamum_indicum
Fang	2016	Setaria_italica
Bergero	2013	Silene_latifolia
Javidfar	2013	Sinapis_alba
Viquez-Zamo	2014	Solanum_lycopersicum
Fukuoka	2012	Solanum_melongena
vandenOever	2016	Solanum_pennellii
Chen	2014	Solanum_pimpinellifolium
Endelman	2016	Solanum_tuberosum
Ji	2017	Sorghum_bicolor

Palaiokostas	2016	Sparus_aurata
Chan-Navarr[illegible]	2016	Spinacia_oleracea
Nemetschke	2010	Strongyloides_ratti
Tortereau	2012	Sus_scrofa
Backstrom	2010	Taeniopygia_guttata
Kai	2005	Takifugu_rubripes
Arias	2016	Taraxacum_koksaghyz
Royaert	2016	Theobroma_cacao
Uchino	2016	Thunnus_orientalis
Foley	2011	Tigriopus_californicus
Lorenzen	2005	Tribolium_castaneum
Yezerski	2003	Tribolium_confusum
Laurent	1998	Trichogramma_brassicae
Hirakawa	2016	Trifolium_pratense
Griffiths	2013	Trifolium_repens
Ghamkhar	2012	Trifolium_subterraneum
Zhai	2016	Triticum_aestivum
MacLeod	2005	Trypanosoma_brucei
Shan	2015	Undaria_pinnatifida
McCallum	2016	Vaccinium_corymbosum
Covarrubias-[illegible]	2016	Vaccinium_macrocarpon
Lepers-Andrz[illegible]	2012	Vanilla_tahitensis
Sirvio	2011	Vespula_vulgaris
Kaur	2014	Vicia_faba
Liu	2016	Vigna_angularis
Gupta	2008	Vigna_mungo
Wang	2016	Vigna_radiata
Kongjaimun	2012	Vigna_unguiculata
Marubodee	2015	Vigna_vexillata
Liu	2013	Vitis_amurensis
Zhang	2009	Vitis_cinerea
Guo	2015	Vitis_vinifera
Wells	2011	Xenopus_tropicalis
Amores	2014	Xiphophorus_maculatus
Zhou	2016	Zea_mays
Zhang	2016	Ziziphus_jujuba
Wang	2015	Zoysia_japonica
Huang	2016	Zoysia_matrella
Lendenmann	2014	Zymoseptoria_tritici

speciesOTT	Group	par.path	Genome Size
Acacia_mangium_ott444202	Plants	n	635.7
Acromyrmex_echinatior_ott735589	Animals	n	335
Acropora_millepora_ott5908120	Animals	n	420
Actinidia_chinensis_ott279981	Plants	n	1408.32
Acyrthosiphon_pisum_ott814484	Animals	n	165
Aedes_aegypti_ott269666	Animals	n	942.14
Aedes_albopictus_ott793181	Animals	n	1090.47
Aegilops_sharonensis_ott327444	Plants	n	6894.9
Aegilops_tauschii_ott881533	Plants	n	4327.21
Aegilops_umbellulata_ott790374	Plants	n	4938.9
Agaricus_bisporus_ott564405	Fungi	n	30.2337
Agrostis_capillaris_ott550221	Plants	n	4000.02
Agrostis_stolonifera_ott307879	Plants	n	3423
Allium_cepa_ott781600	Plants	n	16381.5
Amaranthus_hybridus_ott317810	Plants	n	616.14
Ambystoma_tigrinum_ott1092552	Animals	n	30875.46
Ananas_comosus_ott627039	Plants	n	537.9
Anas_platyrhynchos_ott765167	Animals	n	1375.72
Anguilla_japonica_ott854198	Animals	n	1151.14
Anopheles_funestus_ott286257	Animals	n	225.224
Anoplopoma_fimbria_ott441856	Animals	n	757.95
Anthurium_longicaudatum_ott3999674	Plants	n	4694.4
Apis_cerana_ott625771	Animals	n	185.82
Apis_mellifera_ott461645	Animals	n	229.83
Apostichopus_japonicus_ott721060	Animals	n	664.37
Arabidopsis_halleri_ott152273	Plants	n	234.72
Arabidopsis_thaliana_ott309263	Plants	n	123.5
Arachis_duranensis_ott607569	Plants	n	1242.06
Arachis_hypogaea_ott39541	Plants	n	2806.86
Argopecten_irradians_ott6370122	Animals	n	1173.6
Argopecten_irradians_ott887220	Animals	n	865.53
Artemia_franciscana_ott59094	Animals	n	948.66
Didymella_rabiei_ott5712886	Fungi	y	34.6583
Aspergillus_nidulans_ott117339	Fungi	n	30.2427
Astatotilapia_ott710024	Animals	n	948.66
Astyanax_mexicanus_ott701518	Animals	n	1191.24
Avena_sativa_ott790381	Plants	n	12938.94
Bactrocera_cucurbitae_ott227727	Animals	n	373
Barbarea_vulgaris_ott35910	Plants	n	167.352
Begonia_plebeja_ott434483	Plants	n	616.14
Beta_vulgaris_ott273185	Plants	n	1222.5
Betula_pendula_ott267719	Plants	n	1369.2
Mycalesis_anynana_ott3108421	Animals	n	479.22
Bison_bison_ott907693	Animals	n	4792.2

Biston_betularia_ott968114	Animals	n	500
Boehmeria_nivea_ott594991	Plants	n	716
Bombus_terrestris_ott161197	Animals	n	518.34
Bombyx_mori_ott440274	Animals	n	513.45
Bos_taurus_ott490099	Animals	n	3537.1
Bracon_brevicornis_ott3293364	Animals	y	165
Brassica_rapa_subsp_oleifera_ott240937	Plants	n	567.24
Brassica_carinata_ott749459	Plants	n	1545.24
Brassica_juncea_ott309279	Plants	n	1496.34
Brassica_napus_ott309234	Plants	n	1124.7
Brassica_oleracea_ott833642	Plants	n	762.8
Brassica_rapa_ott833632	Plants	n	782.4
Cajanus_cajan_ott612448	Plants	n	860.64
Camelina_sativa_ott776348	Plants	n	641.356
Camellia_sinensis_ott1058509	Plants	n	3814.2
Canis_lupus_ott247341	Animals	n	2748.18
Capra_hircus_ott19017	Animals	n	3168.72
Capsella_bursa_pastoris_ott833644	Plants	n	391.2
Capsicum_annuum_ott473836	Plants	n	3090.48
Capsicum_baccatum_ott232458	Plants	n	3628.38
Carassius_auratus_ott1005907	Animals	n	1643.04
Carica_papaya_ott429474	Plants	n	372
Carthamus_tinctorius_ott1061541	Plants	n	1369.2
Castanea_mollissima_ott540978	Plants	n	833.241
Catharanthus_roseus_ott524094	Plants	n	2376.5
Ceratopteris_cornuta_ott609865	Plants	n	11294
Chenopodium_quinoa_ott117198	Plants	n	1447.44
Chimonanthus_praecox_ott324777	Plants	n	600
Chlamydomonas_reinhardtii_ott33153	Plants	n	120.405
Notochlamys_hexactes_ott100122	Animals	n	1244.016
Cicer_arietinum_ott612429	Plants	n	929.1
Ciona_intestinalis_ott744224	Animals	n	150
Ciona_savignyi_ott677382	Animals	n	170
Citrullus_lanatus_ott1006251	Plants	n	440.1
Citrus_aurantium_ott3942423	Plants	n	301.365
Citrus_sinensis_ott3942433	Plants	n	490.956
Clunio_marinus_ott192116	Animals	n	95
Cocos_nucifera_ott366250	Plants	n	3107.106
Coffea_arabica_ott1001050	Plants	n	1173.6
Coprinopsis_cinerea_ott737441	Fungi	n	37.5
Corchorus_capsularis_ott679189	Plants	n	586.8
Corchorus_olitorius_ott578269	Plants	n	909.54
Coregonus_clupeaformis_ott629345	Animals	n	2386.32
Cornus_florida_ott148264	Plants	n	1545.24
Coturnix_japonica_ott803622	Animals	n	1320.3

Crassostrea_gigas_ott987409	Animals	n	557.736
Crassostrea_virginica_ott365708	Animals	n	675
Crocodylus_porosus_ott35872	Animals	n	2049.54
Cryptococcus_neoformans_ott48333	Fungi	y	19.0519
Cryptomeria_japonica_ott620749	Plants	n	10758
Ctenopharyngodon_idella_ott1005936	Animals	n	1004.54571
Cucumis_melo_ott1006256	Plants	n	929.1
Cucumis_sativus_ott1006246	Plants	n	195.669
Cucurbita_maxima_ott379272	Plants	n	449.88
Cucurbita_moschata_ott84409	Plants	n	420.54
Cucurbita_pepo_ott379282	Plants	n	537.9
Culex_pipiens_ott218684	Animals	n	684.6
Cyanistes_caeruleus_ott746120	Animals	n	1437.66
Cynara_cardunculus_ott991885	Plants	n	1075.8
Cynodon_dactylon_ott927435	Plants	n	1110.03
Cynoglossus_semilaevis_ott906506	Animals	n	606.36
Cyprinus_carpio_ott429083	Animals	n	1741.9158
Dactylis_glomerata_ott83469	Plants	n	4303.2
Danio_rerio_ott1005914	Animals	n	1817.124
Daphnia_magna_ott668392	Animals	n	129.543
Daphnia_pulex_ott59086	Animals	n	197.206
Daucus_carota_ott372836	Plants	n	421.539
Dianthus_caryophyllus_ott842200	Plants	n	616.14
Dicentrarchus_labrax_ott3549	Animals	n	762.84
Drosophila_serrata_ott86632	Animals	n	205.38
Ectocarpus_siliculosus_ott878345	SAR	n	195.811
Eimeria_maxima_ott775254	SAR	y	45.9751
Eimeria_tenella_ott775246	SAR	y	51.8944
Elaeis_guineensis_ott947656	Plants	n	1535.18
Elaeis_oleifera_ott210313	Plants	n	1402.73
Elymus_lanceolatus_ott173152	Plants	n	8244.54
Epinephelus_aeneus_ott306171	Animals	n	1075.8
Equus_caballus_ott1068218	Animals	n	3149.16
Eremochloa_ophiuroides_ott843929	Plants	n	811.74
Eriobotrya_japonica_ott207983	Plants	n	762.84
Eriocheir_sinensis_ott566723	Animals	n	1680
Esox_lucius_ott739941	Animals	n	1095.36
Eucalyptus_camaldulensis_ott505234	Plants	n	606.36
Eucalyptus_grandis_ott40959	Plants	n	591.5922
Eucalyptus_tereticornis_ott173799	Plants	n	586.8
Eucalyptus_urophylla_ott1049231	Plants	n	595.602
Eucommia_ulmoides_ott743836	Plants	n	723.72
Fagopyrum_esculentum_ott904381	Plants	n	1359.4
Felis_silvestris_ott563163	Animals	n	2894.88
Festuca_arundinacea_ott105576	Animals	n	1660

Festuca_rubra_ott202297	Plants	n	4185.84
Ficedula_albicollis_ott107840	Animals	n	1118.34
Fragaria_iinumae_ott555467	Plants	n	199.628
Fragaria_vesca_ott852873	Plants	n	214.373
Fusarium_circinatum_FSP_34_ott75556	Plants	n	697.762
Fusarium_graminearum_CS3005_ott5726284	Fungi	y	43.9486
Fusarium_verticillioides_7600_ott966934	Fungi	y	36.458
Fusarium_oxysporum_f.sp._conglutinans_ott5481360	Fungi	n	61.3869
Fusarium_verticillioides_ott966934	Fungi	y	41.8449
Gadus_morhua_ott114170	Animals	n	909.54
Gallus_gallus_ott153563	Animals	n	1230.26
Gasterosteus_aculeatus_ott401066	Animals	n	625.92
Gentiana_scabra_ott717875	Plants	n	5000
Globodera_rostochiensis_ott232963	Animals	y	95.8763
Glycine_max_ott681762	Plants	n	1105.14
Gossypium_barbadense_ott854476	Plants	n	2694.39
Gossypium_hirsutum_ott854480	Plants	n	2347.2
Gossypium_raimondii_ott79347	Plants	n	1101.228
Gossypium_tomentosum_ott733231	Plants	n	2386.32
Haliotis_diversicolor_ott780542	Animals	n	1418.1
Haliotis_midae_ott780532	Animals	n	1398.54
Haplochromis_chilotes_ott138490	Animals	n	1075.8
Haplochromis_sauvagei_ott685068	Animals	n	1075.8
Helianthus_annuus_ott515712	Plants	n	2376.54
Heliconius_melpomene_ott896444	Animals	n	293.4
Heterodera_glycines_ott332065	Animals	y	81.908
Hevea_brasiliensis_ott339361	Plants	n	2102.7
Hibiscus_cannabinus_ott393745	Plants	n	1496.34
Hieracium_mixtum_ott711719	Plants	n	4058.7
Hippoglossus_hippoglossus_ott408328	Animals	n	713.94
Hordeum_vulgare_ott657948	Plants	n	5418.1
Humulus_lupulus_ott84020	Plants	n	2836.2
Hyla_arborea_ott677266	Animals	n	4649.69
Hypophthalmichthys_molitrix_ott35786	Animals	n	978
Hyriopsis_cumingii_ott351029	Animals	n	3000
Ictalurus_punctatus_ott701523	Animals	n	993.648
Ipomoea_batatas_ott1071040	Plants	n	1833.75
Ixodes_scapularis_ott621065	Animals	n	1765.38
Jatropha_curcas_ott916001	Plants	n	420.54
Juglans_regia_ott138717	Plants	n	606.36
Kryptolebias_marmoratus_ott229709	Animals	n	680.367
Labeo_rohita_ott160616	Animals	n	1950
Lactuca_sativa_ott515700	Plants	n	2591.7
Larimichthys_crocea_ott679823	Animals	n	678.938
Larix_kaempferi_ott154040	Plants	n	9291

Lates_calcarifer_ott6362446	Animals	n	684.6
Lens_culinaris_ott156245	Plants	n	4107.6
Lepomis_macrochirus_ott836783	Animals	n	987.78
Leptinotarsa_decemlineata_ott185597	Animals	n	449.88
Linum_usitatissimum_ott1000262	Plants	n	684.6
Penaeus_vannamei_ott169168	Animals	n	2640
Lolium_multiflorum_ott135777	Plants	n	2660.16
Lolium_perenne_ott135774	Plants	n	2365.782
Lotus_japonicus_ott1081728	Plants	n	469.44
Lucania_goodei_ott84261	Animals	n	1319.322
Lucania_parva_ott839518	Animals	n	1391.694
Luffa_acutangula_ott565719	Plants	n	831.3
Lupinus_albus_ott1030424	Plants	n	586.8
Lupinus_angustifolius_ott1030433	Plants	n	1153
Macaca_mulatta_ott689650	Animals	n	3290.97
Macropus_eugenii_ott539171	Animals	n	1153
Magnaporthe_grisea_ott258235	Fungi	y	42.711
Malus_domestica_ott3902985	Plants	n	1874.77
Malus_sieversii_ott321926	Plants	n	733.5
Malus_pumila_ott854961	Plants	n	753.06
Mangifera_foetida_ott422407	Plants	n	440.1
Manihot_esculenta_ott339369	Plants	n	1222.5
Penaeus_japonicus_ott5787510	Animals	n	2767.74
Medicago_sativa_ott1086789	Plants	n	1276.29
Medicago_truncatula_ott1060499	Plants	n	469.44
Melampsora_lini_ott809207	Fungi	y	170.172
Meleagris_gallopavo_ott446482	Animals	n	1431.14
Meloidogyne_hapla_ott1043271	Animals	y	53.013
Melospiza_melodia_ott265548	Animals	n	1398.54
Maylandia_zebra_ott677719	Animals	n	848.776495
Microtus_ochrogaster_ott927240	Animals	n	2287.34
Erythranthe_guttata_ott504496	Plants	n	450
Miscanthus_sinensis_ott764796	Plants	n	2591.7
Misgurnus_anguillicaudatus_ott312382	Animals	n	1594.14
Monodelphis_domestica_ott122362	Animals	n	3598.44
Musa_acuminata_ott207474	Plants	n	472.231
Mycosphaerella_graminicola_ott5319573	Fungi	y	39.7
Nasonia_giraulti_ott729592	Animals	n	283.607
Nelumbo_lutea_ott532278	Plants	n	948.66
Nelumbo_nucifera_ott532288	Plants	n	234.72
Nicotiana_langsdorffii_ott733968	Plants	n	4254.3
Nicotiana_tabacum_ott222787	Plants	n	5066.04
Nilaparvata_lugens_ott876508	Animals	n	1140.79
Nothobranchius_furzeri_ott513643	Animals	n	1242.52
Olea_europaea_ott23729	Plants	n	1907.1

Oncorhynchus_kisutch_ott739917	Animals	n	2767.74
Oncorhynchus_mykiss_ott5256670	Animals	n	2592.678
Oncorhynchus_nerka_ott165375	Animals	n	2858.694
Oncorhynchus_tshawytscha_ott730762	Animals	n	2709.06
Oreochromis_mossambicus_ott288069	Animals	n	978
Oreochromis_niloticus_ott288063	Animals	n	1058.196
Oryctolagus_cuniculus_ott864596	Animals	n	3107.24571
Oryza_rufipogon_ott135756	Plants	n	449.88
Oryza_sativa_ott709894	Plants	n	489
Ostrea_edulis_ott1033104	Animals	n	1144.26
Ovis_aries_ott70819	Animals	n	2941.824
Ovis_canadensis_ott123206	Animals	n	2590.55
Panicum_virgatum_ott205619	Plants	n	1792.674
Papilio_glaucus_ott947948	Animals	n	430.32
Papio_hamadryas_ott115463	Animals	n	3457.23
Paralichthys_olivaceus_ott883392	Animals	n	694.38
Parus_major_ott515143	Animals	n	1476.78
Mizuhopecten_yessoensis_ott6370603	Animals	n	1437.66
Penaeus_monodon_ott212713	Animals	n	2170
Pennisetum_schweinfurthii_ott1027230	Plants	n	2621.04
Peromyscus_maniculatus_ott377525	Animals	n	4264.08
Persea_americana_ott913249	Plants	n	909.54
Petunia_axillaris_ott232467	Plants	n	1369.2
Phaeosphaeria_nodorum_ott5346507	Fungi	y	37.1
Phalloceros_caudimaculatus_ott808637	Animals	n	700
Phaseolus_vulgaris_ott825707	Plants	n	586.8
Phoenix_dactylifera_ott789008	Plants	n	929.1
Physcomitrella_patens_ott821359	Plants	n	477.948
Phytophthora_infestans_ott775725	SAR	y	240
Picea_abies_ott517943	Plants	n	18190.8
Pinus_balfouriana_ott1044480	Plants	n	29281.32
Pinus_elliottii_ott245809	Plants	n	22787.4
Pinus_koraiensis_ott830806	Plants	n	27579.6
Pinus_lambertiana_ott568626	Plants	n	27602.7
Pinus_massoniana_ott714296	Plants	n	25134.6
Pinus_pinaster_ott837883	Plants	n	23814.3
Pinus_radiata_ott568644	Plants	n	21936.54
Pinus_taeda_ott1075736	Plants	n	21516
Pinus_thunbergii_ott1075728	Plants	n	4772.64
Plasmodium_chabaudi_ott724404	SAR	y	18.8678
Plasmodium_falciparum_ott201128	SAR	y	23.2703
Poecilia_reticulata_ott312452	Animals	n	865.53
Pogonomyrmex_rugosus_ott1042743	Animals	n	255
Populus_adenopoda_ott5763644	Plants	n	508.56
Populus_alba_ott1030862	Plants	n	508.56

Populus_deltoides_ott8867	Plants	n	500
Populus_nigra_ott8848	Plants	n	514.0368
Populus_tremula_ott839584	Plants	n	469.44
Portunus_trituberculatus_ott718120	Animals	n	2259.18
Primulina_eburnea_ott5264953	Plants	n	1985.34
Pristionchus_pacificus_ott197875	Animals	n	133.114
Prunus_armeniaca_ott217632	Plants	n	293.4
Prunus_avium_ott162547	Plants	n	342.3
Prunus_dulcis_ott731568	Plants	n	240
Prunus_kansuensis_ott1020749	Plants	n	293.4
Prunus_mume_ott426562	Plants	n	234.03
Prunus_persica_ott259054	Plants	n	273.84
Pseudocercospora_eucalypticola_ott4073293	Fungi	y	74.1412
Punica_granatum_ott646814	Plants	n	704.16
Pyrus_communis_ott972654	Plants	n	537.9
Pyrus_ussuriensis_ott116277	Plants	n	909.5
Rana_temporaria_ott14718	Animals	n	4169.21
Raphanus_sativus_ott359073	Plants	n	978
Rattus_norvegicus_ott271555	Animals	n	3286.08
Rhyzopertha_dominica_ott633644	Animals	n	476
Ricinus_communis_ott339346	Plants	n	508.56
Rubus_idaeus_ott156929	Plants	n	311.982
Rubus_occidentalis_ott670811	Plants	n	293.4
Ruditapes_philippinarum_ott913666	Animals	n	1967
Saccharina_japonica_ott246789	SAR	n	543.426
Salmo_salar_ott688328	Animals	n	3048.426
Salmo_trutta_ott688332	Animals	n	2875.32
Salvia_miltiorrhiza_ott847543	Plants	n	615
Schistosoma_mansoni_ott191483	Animals	y	364.538
Sciaenops_ocellatus_ott433079	Animals	n	381.42
Scleropages_formosus_ott335717	Animals	n	777.359
Scophthalmus_maximus_ott778695	Animals	n	841.08
Scylla_paramamosain_ott1084541	Animals	n	1603.92
Secale_cereale_ott553015	Plants	n	8097.84
Seriola_quinqueradiata_ott186469	Animals	n	811.74
Sesamum_indicum_ott504628	Plants	n	948.66
Setaria_italica_ott553005	Plants	n	518.34
Silene_latifolia_ott609504	Plants	n	2640.6
Sinapis_alba_ott359058	Plants	n	489
Solanum_lycopersicum_ott378964	Plants	n	823.786
Solanum_melongena_ott494843	Plants	n	958.44
Solanum_pennellii_ott1069768	Plants	n	926.426
Solanum_pimpinellifolium_ott797186	Plants	n	688.247
Solanum_tuberosum_ott494835	Plants	n	860.64
Sorghum_bicolor_ott552986	Plants	n	1188.27

Sparus_aurata_ott760723	Animals	n	929.1
Spinacia_oleracea_ott317784	Plants	n	1007.3
Strongyloides_ratti_ott1040287	Animals	y	43.1502
Sus_scrofa_ott730013	Animals	n	2922.264
Taeniopygia_guttata_ott708327	Animals	n	1222.5
Takifugu_rubripes_ott65341	Animals	n	391.2
Taraxacum_kok_saghyz_ott843397	Plants	n	1420
Theobroma_cacao_ott388185	Plants	n	420.5
Thunnus_orientalis_ott833202	Animals	n	800
Tigriopus_californicus_ott461524	Animals	n	244.5
Tribolium_castaneum_ott148904	Animals	n	202.935
Tribolium_echinatum_ott677520	Animals	n	245
Trichogramma_brassicae_ott137951	Animals	y	246
Trifolium_pratense_ott839027	Plants	n	528.12
Trifolium_repens_ott116218	Plants	n	1095.36
Trifolium_subterraneum_ott539553	Plants	n	547.68
Triticum_aestivum_ott31926	Plants	n	16948.74
Trypanosoma_brucei_ott494245	SAR	y	22.1481
Undaria_pinnatifida_ott616561	SAR	n	560
Vaccinium_corymbosum_ott567253	Plants	n	655.26
Vaccinium_macrocarpon_ott295602	Plants	n	414.622
Vanilla_tahitensis_ott1064791	Plants	n	5603.94
Vespula_vulgaris_ott52839	Animals	n	219.8
Vicia_faba_ott539544	Plants	n	17622.582
Vigna_angularis_ott1065959	Plants	n	537.9
Vigna_mungo_ott1065954	Plants	n	537.9
Vigna_radiata_ott806245	Plants	n	894.87
Vigna_unguiculata_ott1065941	Plants	n	586.8
Vigna_vexillata_ott581960	Plants	n	567.24
Vitis_amurensis_ott805071	Plants	n	489
Vitis_cinerea_ott314458	Plants	n	489
Vitis_vinifera_ott756728	Plants	n	446.946
Xenopus_tropicalis_ott940184	Animals	n	1440.4
Xiphophorus_maculatus_ott416852	Animals	n	885.09
Zea_mays_ott605194	Plants	n	2669.9
Ziziphus_jujuba_ott73483	Plants	n	437.754
Zoysia_japonica_ott586007	Plants	n	420.54
Zoysia_matrella_ott205630	Plants	n	563.439
Zymoseptoria_tritici_ott906425	Fungi	y	39.6863

HCN	MapLength.c	Sexual.sys	Ref.#
13	966	NA	1
19	2033.8	male.haploid	2
14	1391	hermaphrodi	3
29	3445	NA	4
5	394	parthenogen	5
3	235	gonochorous	6
3	212.6	gonochorous	7
7	818	NA	8
7	1374.4	NA	9
7	948.72	NA	10
13	1156	NA	11
14	1156	NA	12
14	1424	NA	13
9	808	NA	14
16	1288	NA	15
14	5251.3	gonochorous	16
25	2113	NA	17
40	1766	gonochorous	18
19	1436.5	gonochorous	19
3	145	gonochorous	20
24	1332.8	NA	21
15	1023.5	NA	22
16	574.5	male.haploid	23
16	4115	male.haploid	24
22	3706.6	gonochorous	25
8	567	NA	26
5	422.5	NA	27
10	1081.3	NA	28
20	1446.7	NA	29
16	849.3	NA	30
24	1917.3	gonochorous	31
21	1177	gonochorous	32
14	1271	NA	33
8	3624.7	NA	34
20	1249.3	NA	35
25	2110.7	gonochorous	36
21	2843	NA	37
5	646	gonochorous	38
8	889	NA	39
14	1099	NA	40
9	555	NA	41
14	2489.7	NA	42
28	1642.2	gonochorous	43
30	2647	gonochorous	44

31	1638	gonochorous	45
14	2265.1	NA	46
18	2047	male.haploid	47
28	3320	gonochorous	48
31	3159.1	gonochorous	49
10	536.1	male.haploid	50
10	973.3	NA	51
17	2048	NA	52
18	1579	NA	53
19	1917.9	NA	54
9	890.01	NA	55
10	858.98	NA	56
11	1059	NA	57
20	1385.6	NA	58
15	1218	NA	59
39	2085.1	gonochorous	60
30	2737	gonochorous	61
16	1064.4	NA	62
12	1372	NA	63
12	1056	NA	64
50	5252	NA	65
18	1069.9	NA	66
12	959.44	NA	67
12	743	NA	68
6	1790.4	NA	69
39	2178.8	NA	70
18	1404	NA	71
11	1801	NA	72
17	1000	NA	73
19	1561.8	NA	74
8	1083.93	NA	75
14	4218.9	hermaphrodi	76
14	650	hermaphrodi	77
11	1096	NA	78
9	1084.1	NA	79
9	976.58	NA	80
3	179.65	gonochorous	81
16	2739	NA	82
22	3800	NA	83
13	1346	NA	84
7	2016	NA	85
7	358.5	NA	86
40	3061	NA	87
7	1175	NA	88
32	2816	gonochorous	89

10	588	seq.hermaph	90
10	905	NA	91
17	1071.55	gonochorous	92
14	1500	NA	93
11	1266.2	NA	94
24	1176.1	gonochorous	95
12	1150	NA	96
7	1061.19	NA	97
20	2533.8	NA	98
20	1936	NA	99
20	2817.6	NA	100
3	189.9	gonochorous	101
39	935	gonochorous	102
17	1251.15	NA	103
18	1055	NA	104
21	1624	gonochorous	105
50	3565.9	gonochorous	106
7	715.77	NA	107
25	2177.363	gonochorous	108
10	1614.5	parthenogen	109
12	1206	parthenogen	110
9	635.1	NA	111
15	978.3	NA	112
24	1373.1	NA	113
4	245.3	gonochorous	114
34	2459.3	NA	115
14	2883.9	NA	116
12	653	NA	117
16	1499.5	NA	118
16	1485	NA	119
14	2574	NA	120
24	969.5	hermaphrodi	121
32	2772	gonochorous	122
9	623.6	NA	123
17	1036	NA	124
73	5125.53	gonochorous	125
25	1289.3	gonochorous	126
11	1236	NA	127
11	1107	NA	128
11	1241.4	NA	129
11	1208.5	NA	130
17	2133	NA	131
8	1574.3	NA	132
19	4464	gonochorous	133
44	1895.795	gonochorous	134

21	1417.5	NA	135
30	1787	gonochorous	136
7	451.7	NA	137
7	416.2	NA	138
28	1820	NA	139
11	2774.4	parthenogen	140
4	1140	parthenogen	141
15	86	parthenogen	142
11	2188	parthenogen	143
23	1421.92	gonochorous	144
35	2762.2	gonochorous	145
22	1980.74	gonochorous	146
13	613.7	NA	147
9	431	gonochorous	148
20	2445.8	NA	149
26	5115.6	NA	150
26	4071.98	NA	151
13	2325	NA	152
26	3320.8	NA	153
16	2190.1	NA	154
18	1038	NA	155
22	1225.68	NA	156
22	1130.63	NA	157
17	2472	NA	158
21	1364.23	gonochorous	159
9	539	gonochorous	160
18	4160	NA	161
18	4924.8	NA	162
18	1650	NA	163
24	1514	gonochorous	164
7	927.07	NA	165
10	745.9	NA	166
12	1770.722	gonochorous	167
24	1561.1	gonochorous	168
19	2713	NA	169
29	3240	gonochorous	170
90	8184.5	NA	171
15	616	gonochorous	172
11	1655.8	NA	173
16	2457.82	NA	174
24	1248	NA	175
25	1373.8	NA	176
9	2974	NA	177
24	5451.3	NA	178
12	546.5	NA	179

24	1412.9	hermaphrodi	180
7	2429.61	NA	181
24	1576.75	NA	182
18	1032	gonochorous	183
15	1151	NA	184
44	4271.43	gonochorous	185
7	488.8	NA	186
7	952.6	NA	187
6	508.5	NA	188
24	392	gonochorous	189
23	605	gonochorous	190
13	1436.12	NA	191
25	2169	NA	192
20	1629.9	NA	193
22	2048	gonochorous	194
7	1402.4	gonochorous	195
7	1247	NA	196
17	1267	NA	197
17	1299.67	NA	198
17	1481.72	NA	199
20	3148.28	NA	200
18	2412	NA	201
43	3610.9	gonochorous	202
32	2133	NA	203
8	1215	NA	204
18	5860	NA	205
32	2324	gonochorous	206
16	1294.1	parthenogen	207
40	1731	gonochorous	208
22	1933	NA	209
28	1707	gonochorous	210
14	1750	NA	211
19	1605.5	NA	212
25	723.35	gonochorous	213
9	715	gonochorous	214
11	1197	NA	215
21	1216	NA	216
5	620	male.haploid	217
8	494.3	NA	218
8	581.3	NA	219
9	1062	NA	220
24	2662.43	NA	221
15	1093.3	gonochorous	222
19	1965	NA	223
23	3049	NA	224

30	358.55	gonochorous
29	3600	gonochorous
29	3186.5	NA
34	3119.7	gonochorous
22	1067.6	NA
22	704	gonochorous
22	1419	gonochorous
12	1596.8	NA
12	1650	NA
10	536.4	NA
27	3304	gonochorous
27	3051	gonochorous
18	2085.2	NA
28	1167	gonochorous
20	2354	gonochorous
24	1695.15	gonochorous
32	1916.82	gonochorous
19	1918.65	NA
44	3488.5	gonochorous
7	716.7	NA
24	1499	gonochorous
12	1087.4	NA
7	970	NA
17	1932.1	NA
24	1477	NA
11	1042.2	NA
18	1293	NA
27	4410	NA
9	1091	NA
12	1889.2	NA
12	1572.8	NA
12	1036.3	NA
12	857.46	NA
12	1231	NA
12	1956	NA
12	1708	NA
12	1060	NA
12	2305	NA
7	1255	NA
14	1676	NA
14	2514	NA
23	899	gonochorous
16	2823	male.haploid
19	2178.5	NA
19	3027.1	NA

19	4067.16	NA	270
19	2278.5	NA	271
19	1542.35	NA	272
53	3519.45	gonochorous	273
18	3774.7	NA	274
6	338.6	NA	275
8	522.69	NA	276
8	849	NA	277
8	591.4	NA	278
8	616	NA	279
8	1550.62	NA	280
8	389.2	NA	281
12	1416	NA	282
8	1141	NA	283
17	2243.4	NA	284
12	742	NA	285
13	1698.8	gonochorous	286
9	1538	NA	287
22	1503	gonochorous	288
9	390.1	NA	289
10	1551	NA	290
7	462.7	NA	291
7	309	NA	292
19	1926.98	NA	293
31	1782.75	NA	294
29	5961	gonochorous	295
40	1403	gonochorous	296
8	1516	NA	297
8	1134.84	gonochorous	298
24	1815.3	NA	299
22	2218.3	gonochorous	300
22	2622.09	NA	301
44	2746.4	gonochorous	302
7	1593	NA	303
24	1128.595	NA	304
16	2981.28	NA	305
9	1318.8	NA	306
12	671.1	NA	307
12	890.4	NA	308
12	1297.3	NA	309
12	1285.5	NA	310
12	789.7	NA	311
12	1567.5	NA	312
12	883	NA	313
10	2158	NA	314

24	3899	hermaphrodi	315
6	433.6	NA	316
3	90.2	parthenogen	317
20	2012	gonochorous	318
35	1479	gonochorous	319
22	1696.3	gonochorous	320
8	894.1	NA	321
10	852.8	NA	322
24	1162.6	gonochorous	323
12	401	gonochorous	324
10	571	gonochorous	325
8	968	gonochorous	326
5	1330	male.haploid	327
7	2084	NA	328
16	1274	NA	329
8	1701.7	NA	330
21	2875.3	NA	331
11	1157.5	NA	332
30	1816.28	NA	333
12	1621	NA	334
12	1112	NA	335
13	1035.85	NA	336
25	2129	male.haploid	337
6	1216.8	NA	338
11	1628.15	NA	339
11	865.1	NA	340
11	732.9	NA	341
11	852.4	NA	342
11	973.9	NA	343
19	1123	NA	344
19	1155.98	NA	345
19	1929.13	NA	346
10	1668	gonochorous	347
24	1328.3	gonochorous	348
10	1545.65	NA	349
12	1456.53	NA	350
20	1337.2	NA	351
20	1824.95	NA	352
21	4255.4	NA	353
