# Supplementary material for: Variation in recombination frequency and distribution across eukaryotes: patterns and processes
Source: Philos Trans R Soc Lond B Biol Sci. 2017 Nov 6;372(1736):20160455. doi: 10.1098/rstb.2016.0455 (PMC5698618; doi:10.1098/rstb.2016.0455)
Supplement: Reference list for linkage map data [file rstb20160455supp3.pdf]

Ref.#	First Author	Year	Reference
1	Butcher	2000	Butcher, P. A., and G. F. Moran. 2000. Genetic linkage mapping in Acacia mangium. 2. Development of an integrated map from two outbred pedigrees using RFLP and microsatellite loci. Theoretical and Applied Genetics **101**:594-
2	Sirvio	2011	Sirvio, A., J. Gadau, O. Rueppell, D. Lamatsch, J. J. Boomsma, P. Pamilo, and R. E. Page. 2006. High recombination frequency creates genotypic diversity in colonies of the leaf-cutting ant Acromyrmex echinatior. Journal of
3	Wang	2009	Wang, S., L. L. Zhang, E. Meyer, and M. V. Matz. 2009. Construction of a high-resolution genetic linkage map and comparative genome analysis for the reef-building coral Acropora millepora. Genome Biology **10**.
4	Scaglione	2015	Scaglione, D., A. Fornasiero, C. Pinto, F. Cattonaro, A. Spadotto, R. Infante, C. Meneses, R. Messina, O. Lain, G. Cipriani, and R. Testolin. 2015. A RAD-based linkage map of kiwifruit (Actinidia chinensis Pl.) as a tool to improve the genome assembly and to scan the genomic region of the gender determinant for the marker-assisted breeding. Tree Genetics & Genomes **11**.
5	Hawthorne	2001	Hawthorne, D. J., and S. Via. 2001. Genetic linkage of ecological specialization and reproductive isolation in pea aphids. Nature **412**:904-907.
6	Juneja	2014	Juneja, P., J. Osei-Poku, Y. S. Ho, C. V. Ariani, W. J. Palmer, A. Pain, and F. M. Jiggins. 2014. Assembly of the Genome of the Disease Vector Aedes aegypti onto a Genetic Linkage Map Allows Mapping of Genes Affecting
7	Sutherland	2011	Sutherland, I. W., A. Mori, J. Montgomery, K. L. Fleming, J. M. Anderson, J. G. Valenzuela, D. W. Severson, and W. C. Black. 2011. A Linkage Map of the Asian Tiger Mosquito (Aedes albopictus) Based on cDNA Markers. Journal of
8	Olivera	2013	Olivera, P. D., A. Kilian, P. Wenzl, and B. J. Steffenson. 2013. Development of a genetic linkage map for Sharon goatgrass (Aegilops sharonensis) and mapping of a leaf rust resistance gene. Genome **56**:367-376.
9	Luo	2013	Luo, M. C., Y. Q. Gu, F. M. You, K. R. Deal, Y. Q. Ma, Y. Q. Hu, N. X. Huo, Y. Wang, J. R. Wang, S. Y. Chen, C. M. Jorgensen, Y. Zhang, P. E. McGuire, S. Pasternak, J. C. Stein, D. Ware, M. Kramer, W. R. McCombie, S. F. Kianian, M. M. Martis, K. F. X. Mayer, S. K. Sehgal, W. L. Li, B. S. Gill, M. W. Bevan, H. Simkova, J. Dolezel, W. N. Song, G. R. Lazo, O. D. Anderson, and J. Dvorak. 2013. A 4-gigabase physical map unlocks the structure and evolution of the complex genome of Aegilops tauschii, the wheat D-genome progenitor.
10	Edae	2017	Edae, E. A., P. D. Olivera, Y. Jin, and M. N. Rouse. 2017. Genotyping-by-Sequencing Facilitates a High-Density Consensus Linkage Map for Aegilops umbellulata, a Wild Relative of Cultivated Wheat. G3:
11	Foulogne-Oriol	2010	Foulongne-Oriol, M., C. Spataro, V. Cathalot, S. Monllor, and J. M. Savoie. 2010. An expanded genetic linkage map of an intervarietal Agaricus bisporus var. bisporus x A. bisporus var. burnettii hybrid based on AFLP, SSR and CAPS markers sheds light on the recombination behaviour of the species.
12	Rotter	2009	Rotter, D., K. Amundsen, S. A. Bonos, W. A. Meyer, S. E. Warnke, and F. C. Belanger. 2009. Molecular Genetic Linkage Map for Allotetraploid Colonial Bentgrass. Crop Science **49**:1609-1619.
13	Honig	2014	Honig, J. A., C. Kubik, M. Majewski, C. Poulsen, E. Weibel, K. Amundsen, S. E. Warnke, W. A. Meyer, and S. A. Bonos. 2014. A PCR-based linkage map of Agrostis stolonifera and identification of QTL markers for dollar spot
14	Baldwin	2012	Baldwin, S., R. Revanna, S. Thomson, M. Pither-Joyce, K. Wright, R. Crowhurst, M. Fiers, L. S. Chen, R. Macknight, and J. A. McCallum. 2012. A Toolkit for bulk PCR-based marker design from next-generation sequence data: application for development of a framework linkage map in bulb onion
15	Maughan	2011	Maughan, P. J., S. M. Smith, D. J. Fairbanks, and E. N. Jellen. 2011. Development, Characterization, and Linkage Mapping of Single Nucleotide Polymorphisms in the Grain Amaranths (Amaranthus sp.). Plant Genome **4**:92-
16	Smith	2005	Smith, J. J., D. K. Kump, J. A. Walker, D. M. Parichy, and S. R. Voss. 2005. A comprehensive expressed sequence tag linkage map for tiger salamander and Mexican axolotl: enabling gene mapping and comparative genomics in
17	deSousa	2013	de Sousa, N., J. Carlier, T. Santo, and J. Leitao. 2013. An integrated genetic map of pineapple (Ananas comosus (L.) Merr.). Scientia Horticulturae
18	Huang	2009	Huang, C. W., Y. S. Cheng, R. Rouvier, K. T. Yang, C. P. Wu, H. L. Huang, and M. C. Huang. 2009. Duck (Anas platyrhynchos) linkage mapping by AFLP fingerprinting. Genetics Selection Evolution **41**.

19	Kai	2014	Kai, W., K. Nomura, A. Fujiwara, Y. Nakamura, M. Yasuike, N. Ojima, T. Masaoka, A. Ozaki, Y. Kazeto, K. Gen, J. Nagao, H. Tanaka, T. Kobayashi, and M. Ototake. 2014. A ddRAD-based genetic map and its integration with the genome assembly of Japanese eel (Anguilla japonica) provides insights into genome evolution after the teleost-specific genome duplication. Bmc
20	Wondji	2007	Wondji, C. S., J. Morgan, M. Coetzee, R. H. Hunt, K. Steen, W. C. Black, J. Hemingway, and H. Ranson. 2007. Mapping a quantitative trait locus (QTL) conferring pyrethroid resistance in the African malaria vector Anopheles
21	Rondeau	2013	Rondeau, E. B., A. M. Messmer, D. S. Sanderson, S. G. Jantzen, K. R. von Schalburg, D. R. Minkley, J. S. Leong, G. M. Macdonald, A. E. Davidsen, W. A. Parker, R. S. A. Mazzola, B. Campbell, and B. F. Koop. 2013. Genomics of sablefish (Anoplopoma fimbria): expressed genes, mitochondrial phylogeny, linkage map and identification of a putative sex gene. Bmc Genomics **14**.
22	Venkat	2014	Venkat, S. K., P. Bommisetty, M. S. Patil, L. Reddy, and A. Chennareddy. 2014. The genetic linkage maps of Anthurium species based on RAPID, ISSR and SRAP markers. Scientia Horticulturae **178**:132-137.
23	Shi	2013	Shi, Y. Y., L. X. Sun, Z. Y. Huang, X. B. Wu, Y. Q. Zhu, H. J. Zheng, and Z. J. Zeng. 2013. A SNP Based High-Density Linkage Map of Apis cerana Reveals a High Recombination Rate Similar to Apis mellifera. Plos One **8**.
24	Wilfert	2007	Solignac, M., F. Mougel, D. Vautrin, M. Monnerot, and J. M. Cornuet. 2007. A third-generation microsatellite-based linkage map of the honey bee, Apis mellifera, and its comparison with the sequence-based physical map.
25	Tian	2015	Tian, M. L., Y. P. Li, J. Jing, C. Mu, H. X. Du, J. Z. Dou, J. X. Mao, X. Li, W. Q. Jiao, Y. F. Wang, X. L. Hu, S. Wang, R. J. Wang, and Z. M. Bao. 2015. Construction of a High-Density Genetic Map and Quantitative Trait Locus Mapping in the Sea Cucumber Apostichopus japonicus. Scientific Reports **5**.
26	Willems	2007	Willems, G., D. B. Drager, M. Courbot, C. Gode, N. Verbruggen, and P. Saumitou-Laprade. 2007. The genetic basis of zinc tolerance in the metallophyte Arabidopsis halleri ssp halleri (Brassicaceae): An analysis of
27	Singer	2015	Singer, T., Y. P. Fan, H. S. Chang, T. Zhu, S. P. Hazen, and S. P. Briggs. 2006. A high-resolution map of Arabidopsis recombinant inbred lines by whole-genome exon array hybridization. Plos Genetics **2**:1352-1361.
28	Nagy	2010	Nagy, E. D., Y. Chu, Y. F. Guo, S. Khanal, S. X. Tang, Y. Li, W. B. B. Dong, P. Timper, C. Taylor, P. Ozias-Akins, C. C. Holbrook, V. Beilinson, N. C. Nielsen, H. T. Stalker, and S. J. Knapp. 2010. Recombination is suppressed in an alien introgression in peanut harboring Rma, a dominant root-knot
29	Zhou	2014	Zhou, X. J., Y. L. Xia, X. P. Ren, Y. L. Chen, L. Huang, S. M. Huang, B. S. Liao, Y. Lei, L. Y. Yan, and H. F. Jiang. 2014. Construction of a SNP-based genetic linkage map in cultivated peanut based on large scale marker development using next-generation double-digest restriction-site-associated
30	Li	2012	Li, H. J., X. Liu, and G. F. Zhang. 2012. Consensus Microsatellite-Based Linkage Map for the Hermaphroditic Bay Scallop (Argopecten irradians) and Its Application in Size-Related QTL Analysis. Plos One **7**.
31	Zhu	2014	Zhu, C., J. Tong, X. Yu, W. Guo, X. Wang, H. Liu, X. Feng, Y. Sun, L. Liu, and B. Fu. 2014. A second-generation genetic linkage map for bighead carp (Aristichthys nobilis) based on microsatellite markers. Animal Genetics **45**:699-
32	DeVos	2013	De Vos, S., P. Bossier, G. Van Stappen, I. Vercauteren, P. Sorgeloos, and M. Vuylsteke. 2013. A first AFLP-Based Genetic Linkage Map for Brine Shrimp Artemia franciscana and Its Application in Mapping the Sex Locus. Plos One
33	Phan	2003	Phan, H. T. T., R. Ford, and P. W. J. Taylor. 2003. Mapping the mating type locus of Ascochyta rabiei, the causal agent of ascochyta blight of chickpea.
34	Christians	2011	Christians, J. K., M. S. Cheema, I. A. Vergara, C. A. Watt, L. J. Pinto, N. S. Chen, and M. M. Moore. 2011. Quantitative Trait Locus (QTL) Mapping Reveals a Role for Unstudied Genes in Aspergillus Virulence. Plos One **6**.
35	Sanetra	2009	Sanetra, M., F. Henning, S. Fukamachi, and A. Meyer. 2009. A Microsatellite-Based Genetic Linkage Map of the Cichlid Fish, Astatotilapia burtoni (Teleostei): A Comparison of Genomic Architectures Among Rapidly
36	Carlson	2015	Carlson, B. M., S. W. Onusko, and J. B. Gross. 2015. A High-Density Linkage Map for Astyanax mexicanus Using Genotyping-by-Sequencing Technology. G3-Genes Genomes Genetics **5**:241-251.

37	Chafffin	2016	Chaffin, A. S., Y.-F. Huang, S. Smith, W. A. Bekele, E. Babiker, B. N. Gnanesh, B. J. Foresman, S. G. Blanchard, J. J. Jay, R. W. Reid, C. P. Wight, S. Chao, R. Oliver, E. Islamovic, F. L. Kolb, C. McCartney, J. W. M. Fetch, A. D. Beattie, A. Bjornstad, J. M. Bonman, T. Langdon, C. J. Howarth, C. R. Brouwer, E. N. Jellen, K. E. Klos, J. A. Poland, T.-F. Hsieh, R. Brown, E. Jackson, J. A. Schlueter, and N. A. Tinker. 2016. A Consensus Map in Cultivated Hexaploid Oat Reveals Conserved Grass Synteny with Substantial
38	Sim	2017	Sim, S. B., and S. M. Geib. 2017. A Chromosome-Scale Assembly of the *Bactrocera cucurbitae* Genome Provides Insight to the Genetic Basis of
39	Kuzina	2011	Kuzina, V., J. K. Nielsen, J. M. Augustin, A. M. Torp, S. Bak, and S. B. Andersen. 2011. Barbarea vulgaris linkage map and quantitative trait loci for saponins, glucosinolates, hairiness and resistance to the herbivore Phyllotreta
40	Brennan	2012	Brennan, A. C., S. Bridgett, M. S. Ali, N. Harrison, A. Matthews, J. Pellicer, A. D. Twyford, and C. A. Kidner. 2012. Genomic Resources for Evolutionary Studies in the Large, Diverse, Tropical Genus, Begonia. Tropical Plant
41	Laurent	2007	Laurent, V., P. Devaux, T. Thiel, F. Viard, S. Mielordt, P. Touzet, and M. C. Quillet. 2007. Comparative effectiveness of sugar beet microsatellite markers isolated from genomic libraries and GenBank ESTs to map the sugar beet genome. Theoretical and Applied Genetics **115**:793-805.
42	Jiang	2011	Jiang, T. B., B. R. Zhou, F. L. Gao, and B. Z. Guo. 2011. Genetic linkage maps of white birches (Betula platyphylla Suk. and B. pendula Roth) based on RAPD and AFLP markers. Molecular Breeding **27**:347-356.
43	Beldade	2009	Beldade, P., S. V. Saenko, N. Pul, and A. D. Long. 2009. A Gene-Based Linkage Map for Bicyclus anynana Butterflies Allows for a Comprehensive Analysis of Synteny with the Lepidopteran Reference Genome. Plos Genetics
44	Schnabel	2003	Schnabel, R. D., J. F. Taylor, and J. N. Derr. 2003. Development of a linkage map and QTL scan for growth traits in North American bison. Cytogenetic and
45	VantHof	2013	Van't Hof, A. E., P. Nguyen, M. Dalikova, N. Edmonds, F. Marec, and I. J. Saccheri. 2013. Linkage map of the peppered moth, Biston betularia (Lepidoptera, Geometridae): a model of industrial melanism. Heredity **110**:283-
46	Liu	2014	Liu, T. M., S. W. Tang, S. Y. Zhu, and Q. M. Tang. 2014. QTL mapping for fiber yield-related traits by constructing the first genetic linkage map in ramie (Boehmeria nivea L. Gaud). Molecular Breeding **34**:883-892.
47	Stolle	2011	Stolle, E., L. Wilfert, R. Schmid-Hempel, P. Schmid-Hempel, M. Kube, R. Reinhardt, and R. F. A. Moritz. 2011. A second generation genetic map of the bumblebee Bombus terrestris (Linnaeus, 1758) reveals slow genome and chromosome evolution in the Apidae. Bmc Genomics **12**.
48	Zhan	2009	Zhan, S. A., J. H. Huang, Q. H. Guo, Y. P. Zhao, W. H. Li, X. X. Miao, M. R. Goldsmith, M. W. Li, and Y. P. Huang. 2009. An integrated genetic linkage map for silkworms with three parental combinations and its application to the
49	Ihara	2004	Ihara, N., A. Takasuga, K. Mizoshita, H. Takeda, M. Sugimoto, Y. Mizoguchi, T. Hirano, T. Itoh, T. Watanabe, K. M. Reed, W. M. Snelling, S. M. Kappes, C. W. Beattie, G. L. Bennett, and Y. Sugimoto. 2004. A comprehensive genetic map of the cattle genome based on 3802 microsatellites. Genome Research
50	Holloway	2001	Holloway, A. K., M. R. Strand, W. C. Black, and M. F. Antolin. 2000. Linkage analysis of sex determination in Bracon sp near hebetor (Hymenoptera :
51	Cheng	2009	Cheng, Y., J. F. Geng, J. Y. Zhang, Q. Wang, Q. Y. Ban, and X. L. Hou. 2009. The construction of a genetic linkage map of non-heading Chinese cabbage (Brassica campestris ssp chinensis Makino). Journal of Genetics and
52	Zou	2014	Zou, J., H. Raman, S. M. Guo, D. D. Hu, Z. L. Wei, Z. L. Luo, Y. Long, W. X. Shi, Z. Fu, D. Z. Du, and J. L. Meng. 2014. Constructing a dense genetic linkage map and mapping QTL for the traits of flower development in Brassica carinata. Theoretical and Applied Genetics **127**:1593-1605.
53	Zou	2016	Zou, J., D. D. Hu, P. F. Liu, H. Raman, Z. S. Liu, X. J. Liu, I. A. P. Parkin, B. Chalhoub, and J. L. Meng. 2016. Co-linearity and divergence of the A subgenome of Brassica juncea compared with other Brassica species
54	Zhang	2016	Zhang, Y., C. L. Thomas, J. X. Xiang, Y. Long, X. H. Wang, J. Zou, Z. L. Luo, G. D. Ding, H. M. Cai, N. S. Graham, J. P. Hammond, G. J. King, P. J. White, F. S. Xu, M. R. Broadley, L. Shi, and J. L. Meng. 2016. QTL meta-analysis of root traits in Brassica napus under contrasting phosphorus supply in two

55	Zhao	2016	Zhao, Z. D., H. H. Gu, X. G. Sheng, H. F. Yu, J. S. Wang, L. Huang, and D. Wang. 2016. Genome-Wide Single-Nucleotide Polymorphisms Discovery and High-Density Genetic Map Construction in Cauliflower Using Specific-Locus Amplified Fragment Sequencing. Frontiers in Plant Science **7**.
56	Yu	2016	Yu, S. C., T. B. Su, S. H. Zhi, F. L. Zhang, W. H. Wang, D. S. Zhang, X. Y. Zhao, and Y. J. Yu. 2016. Construction of a sequence-based bin map and mapping of QTLs for downy mildew resistance at four developmental stages in Chinese cabbage (Brassica rapa L. ssp pekinensis). Molecular Breeding
57	Bohra	2012	Bohra, A., R. K. Saxena, B. N. Gnanesh, K. Saxena, M. Byregowda, A. Rathore, P. B. KaviKishor, D. R. Cook, and R. K. Varshney. 2012. An intra-specific consensus genetic map of pigeonpea Cajanus cajan (L.) Millspaugh derived from six mapping populations. Theoretical and Applied Genetics
58	Gehringer	2006	Gehringer, A., W. Friedt, W. Luhs, and R. J. Snowdon. 2006. Genetic mapping of agronomic traits in false flax (Camelina sativa subsp sativa).
59	Taniguchi	2012	Taniguchi, F., K. Furukawa, S. Ota-Metoku, N. Yamaguchi, T. Ujihara, I. Kono, H. Fukuoka, and J. Tanaka. 2012. Construction of a high-density reference linkage map of tea (Camellia sinensis). Breeding Science **62**:263-
60	Wong	2010	Wong, A. K., A. L. Ruhe, B. L. Dumont, K. R. Robertson, G. Guerrero, S. M. Shull, J. S. Ziegle, L. V. Millon, K. W. Broman, B. A. Payseur, and M. W. Neff. 2010. A Comprehensive Linkage Map of the Dog Genome. Genetics **184**:595-
61	Schibler	1998	Schibler, L., D. Vaiman, A. Oustry, C. Giraud-Delville, and E. P. Cribiu. 1998. Comparative gene mapping: A fine-scale survey of chromosome rearrangements between ruminants and humans. Genome Research **8**:901-
62	Linde	2001	Linde, M., S. Diel, and B. Neuffer. 2001. Flowering ecotypes of Capsella bursa-pastoris (L.) Medik. (Brassicaceae) analysed by a cosegregation of phenotypic characters (QTL) and molecular markers. Annals of Botany **87**:91-
63	Han	2016	Han, K., H. J. Jeong, H. B. Yang, S. M. Kang, J. K. Kwon, S. Kim, D. Choi, and B. C. Kang. 2016. An ultra-high-density bin map facilitates high-throughput QTL mapping of horticultural traits in pepper (Capsicum annuum).
64	Lee	2016	Lee, Y. R., J. B. Yoon, and J. Lee. 2016. A SNP-based genetic linkage map of Capsicum baccatum and its comparison to the Capsicum annuum reference physical map. Molecular Breeding **36**.
65	Kuang	2016	Kuang, Y. Y., X. H. Zheng, C. Y. Li, X. M. Li, D. C. Cao, G. X. Tong, W. H. Lv, W. Xu, Y. Zhou, X. F. Zhang, Z. P. Sun, S. Mahboob, K. A. Al-Ghanim, J. T. Li, and X. W. Sun. 2016. The genetic map of goldfish (Carassius auratus) provided insights to the divergent genome evolutions in the Cyprinidae family.
66	Chen	2007	Chen, C. X., Q. Y. Yu, S. B. Hou, Y. J. Li, M. Eustice, R. L. Skelton, O. Veatch, R. E. Herdes, L. Diebold, J. Saw, Y. Feng, W. B. Qian, L. Bynum, L. Wang, P. H. Moore, R. E. Paull, M. Alam, and R. Ming. 2007. Construction of a sequence-tagged high-density genetic map of papaya for comparative
67	Bowers	2016	Bowers, J. E., S. A. Pearl, and J. M. Burke. 2016. Genetic Mapping of Millions of SNPs in Safflower (Carthamus tinctorius L.) via Whole-Genome Resequencing. G3-Genes Genomes Genetics **6**:2203-2211.
68	Kubisiak	2013	Kubisiak, T. L., C. D. Nelson, M. E. Staton, T. Zhebentyayeva, C. Smith, B. A. Olukolu, G. C. Fang, F. V. Hebard, S. Anagnostakis, N. Wheeler, P. H. Sisco, A. G. Abbott, and R. R. Sederoff. 2013. A transcriptome-based genetic map of Chinese chestnut (Castanea mollissima) and identification of regions of segmental homology with peach (Prunus persica). Tree Genetics & Genomes
69	Sharma	2011	Sharma, V., S. Chaudhary, S. Srivastava, R. Pandey, and S. Kumar. 2012. Characterization of variation and quantitative trait loci related to terpenoid indole alkaloid yield in a recombinant inbred line mapping population of
70	Nakazato	2006	Nakazato, T., M. K. Jung, E. A. Housworth, L. H. Rieseberg, and G. J. Gastony. 2006. Genetic map-based analysis of genome structure in the homosporous fern Ceratopteris richardii. Genetics **173**:1585-1597.
71	Maughan	2012	Maughan, P. J., S. M. Smith, J. A. Rojas-Beltran, D. Elzinga, J. A. Raney, E. N. Jellen, A. Bonifacio, J. A. Udall, and D. J. Fairbanks. 2012. Single Nucleotide Polymorphism Identification, Characterization, and Linkage
72	Chen	2010	Chen, D. W., and L. Q. Chen. 2010. The first intraspecific genetic linkage maps of wintersweet Chimonanthus praecox (L.) Link based on AFLP and ISSR markers. Scientia Horticulturae **124**:88-94.

73	Kathir	2003	Kathir, P., M. LaVoie, W. J. Brazelton, N. A. Haas, P. A. Lefebvre, and C. D. Silflow. 2003. Molecular map of the Chlamydomonas reinhardtii nuclear
74	Zhan	2009	Zhan, A., J. Hu, X. Hu, M. Hui, M. Wang, W. Peng, X. Huang, S. Wang, W. Lu, C. Sun, and Z. Bao. 2009. Construction of microsatellite-based linkage maps and identification of size-related quantitative trait loci for Zhikong
75	Gaur	2015	Gaur, R., G. Jeena, N. Shah, S. Gupta, S. Pradhan, A. K. Tyagi, M. Jain, D. Chattopadhyay, and S. Bhatia. 2015. High density linkage mapping of genomic and transcriptomic SNPs for synteny analysis and anchoring the
76	Kano	2007	Kano, S., N. Satoh, and P. Sordino. 2006. Primary Genetic Linkage Maps of the Ascidian, Ciona intestinalis. Zoological Science **23**:31-39.
77	Hill	2008	Hill, M. M., K. W. Broman, E. Stupka, W. C. Smith, D. Jiang, and A. Sidow. 2008. The C. savignyi genetic map and its integration with the reference sequence facilitates insights into chordate genome evolution. Genome
78	Reddy	2014	Reddy, U. K., P. Nimmakayala, A. Levi, V. L. Abburi, T. Saminathan, Y. R. Tomason, G. Vajja, R. Reddy, L. Abburi, T. C. Wehner, Y. Ronin, and A. Karol. 2014. High-Resolution Genetic Map for Understanding the Effect of Genome-Wide Recombination Rate on Nucleotide Diversity in Watermelon.
79	Ollitrault	2012	Ollitrault, P., J. Terol, C. X. Chen, C. T. Federici, S. Lotfy, I. Hippolyte, F. Ollitrault, A. Berard, A. Chauveau, J. Cuenca, G. Costantino, Y. Kacar, L. Mu, A. Garcia-Lor, Y. Froelicher, P. Aleza, A. Boland, C. Billot, L. Navarro, F. Luro, M. L. Roose, F. G. Gmitter, M. Talon, and D. Brunel. 2012. A reference genetic map of C. clementina hort. ex Tan.; citrus evolution inferences from
80	Guo	2015	Guo, F., H. W. Yu, Z. Tang, X. L. Jiang, L. Wang, X. Wang, Q. Xu, and X. X. Deng. 2015. Construction of a SNP-based high-density genetic map for pummelo using RAD sequencing. Tree Genetics & Genomes **11**.
81	Kaiser	2012	Kaiser, T. S., and D. G. Heckel. 2012. Genetic Architecture of Local Adaptation in Lunar and Diurnal Emergence Times of the Marine Midge Clunio marinus (Chironomidae, Diptera). Plos One **7**.
82	Riedel	2009	Riedel, M., M. Riederer, D. Becker, A. Herran, A. Kullaya, G. Arana-Lopez, L. Pena-Rodriguez, N. Billotte, V. Sniady, W. Rohde, and E. Ritter. 2009. Cuticular wax composition in Cocos nucifera L.: physicochemical analysis of wax components and mapping of their QTLs onto the coconut molecular
83	Moncada	2016	Moncada, M. D., E. Tovar, J. C. Montoya, A. Gonzalez, J. Spindel, and S. McCouch. 2016. A genetic linkage map of coffee (Coffea arabica L.) and QTL for yield, plant height, and bean size. Tree Genetics & Genomes **12**.
84	Muraguchi	2003	Muraguchi, H., Y. Ito, T. Kamada, and S. O. Yanagi. 2003. A linkage map of the basidiomycete Coprinus cinereus based on random amplified polymorphic DNAs and restriction fragment length polymorphisms. Fungal Genetics and
85	Biswass	2015	Biswas, C., P. Dey, P. G. Karmakar, and S. Satpathy. 2015. Discovery of large-scale SNP markers and construction of linkage map in a RIL population of jute (Corchorus capsularis). Molecular Breeding **35**.
86	Kundu	2015	Kundu, A., A. Chakraborty, N. A. Mandal, D. Das, P. G. Karmakar, N. K. Singh, and D. Sarkar. 2015. A restriction-site-associated DNA (RAD) linkage map, comparative genomics and identification of QTL for histological fibre content coincident with those for retted bast fibre yield and its major components in jute (Corchorus olitorius L., Malvaceae s. l.). Molecular
87	Gagnaire	2013	Gagnaire, P. A., E. Normandeau, S. A. Pavey, and L. Bernatchez. 2013. Mapping phenotypic, expression and transmission ratio distortion QTL using RAD markers in the Lake Whitefish (Coregonus clupeaformis). Molecular
88	Wang	2009	Wang, X. W., P. A. Wadl, T. A. Rinehart, B. E. Scheffler, M. T. Windham, J. M. Spiers, D. H. Johnson, and R. N. Trigiano. 2009. A linkage map for flowering dogwood (Cornus florida L.) based on microsatellite markers.
89	Kikuchi	2005	Kikuchi, S., D. Fujima, S. Sasazaki, S. Tsuji, M. Mizutani, A. Fujiwara, and H. Mannen. 2005. Construction of a genetic linkage map of Japanese quail (Coturnix japonica) based on AFLP and microsatellite markers. Animal
90	Hedgecock	2015	Hedgecock, D., G. Shin, A. Y. Gracey, D. Van Den Berg, and M. P. Samanta. 2015. Second-Generation Linkage Maps for the Pacific Oyster Crassostrea gigas Reveal Errors in Assembly of Genome Scaffolds. G3-Genes Genomes
91	Yu	2003	Yu, Z. N., and X. M. Guo. 2003. Genetic linkage map of the eastern oyster Crassostrea virginica Gmelin. Biological Bulletin **204**:327-338.

92	Miles	2009	Miles, L. G., S. R. Isberg, T. C. Glenn, S. L. Lance, P. Dalzell, P. C. Thomson, and C. Moran. 2009. A genetic linkage map for the saltwater crocodile (Crocodylus porosus). Bmc Genomics **10**.
93	Marra	2004	Marra, R. E., J. C. Huang, E. Fung, K. Nielsen, J. Heitman, R. Vilgalys, and T. G. Mitchell. 2004. A genetic linkage map of Cryptococcus neoformans variety neoformans serotype D (Filobasidiella neoformans). Genetics **167**:619-631.
94	Moriguchi	2016	Moriguchi, Y., K. Uchiyama, S. Ueno, T. Ujino-Ihara, A. Matsumoto, J. Iwai, D. Miyajima, M. Saito, M. Sato, and Y. Tsumura. 2016. A high-density linkage map with 2560 markers and its application for the localization of the male-sterile genes ms3 and ms4 in Cryptomeria japonica D. Don. Tree Genetics &
95	Xia	2010	Xia, J. H., F. Liu, Z. Y. Zhu, J. J. Fu, J. B. Feng, J. L. Li, and G. H. Yue. 2010. A consensus linkage map of the grass carp (Ctenopharyngodon idella) based on microsatellites and SNPs. Bmc Genomics **11**.
96	Diaz	2011	Diaz, A., M. Fergany, G. Formisano, P. Ziarsolo, J. Blanca, Z. J. Fei, J. E. Staub, J. E. Zalapa, H. E. Cuevas, G. Dace, M. Oliver, N. Boissot, C. Dogimont, M. Pitrat, R. Hofstede, P. van Koert, R. Harel-Beja, G. Tzuri, V. Portnoy, S. Cohen, A. Schaffer, N. Katzir, Y. Xu, H. Y. Zhang, N. Fukino, S. Matsumoto, J. Garcia-Mas, and A. J. Monforte. 2011. A consensus linkage map for molecular markers and Quantitative Trait Loci associated with
97	Zhu	2016	Zhu, W. Y., L. Huang, L. Chen, J. T. Yang, J. N. Wu, M. L. Qu, D. Q. Yao, C. L. Guo, H. L. Lian, H. L. He, J. S. Pan, and R. Cai. 2016. A High-Density Genetic Linkage Map for Cucumber (Cucumis sativus L.): Based on Specific Length Amplified Fragment (SLAF) Sequencing and QTL Analysis of Fruit
98	Zhang	2015	Zhang, G. Y., Y. Ren, H. H. Sun, S. G. Guo, F. Zhang, J. Zhang, H. Y. Zhang, Z. C. Jia, Z. J. Fei, Y. Xu, and H. Z. Li. 2015. A high-density genetic map for anchoring genome sequences and identifying QTLs associated with dwarf vine in pumpkin (Cucurbita maxima Duch.). Bmc Genomics **16**.
99	Gong	2008	Gong, L., M. Pachner, K. Kalai, and T. Lelley. 2008. SSR-based genetic linkage map of Cucurbita moschata and its synteny with Cucurbita pepo.
100	Montero-Pau	2017	Montero-Pau, J., J. Blanca, C. Esteras, E. M. Martínez-Pérez, P. Gómez, A. J. Monforte, J. Cañizares, and B. Picó. 2017. An SNP-based saturated genetic map and QTL analysis of fruit-related traits in Zucchini using
101	Hickner	2013	Hickner, P. V., A. Mori, D. D. Chadee, and D. W. Severson. 2013. Composite Linkage Map and Enhanced Genome Map for Culex pipiens Complex Mosquitoes. Journal of Heredity **104**:649-655.
102	Hansson	2010	Hansson, B., M. Ljungqvist, D. A. Dawson, J. C. Mueller, J. Olano-Marin, H. Ellegren, and J. A. Nilsson. 2010. Avian genome evolution: insights from a linkage map of the blue tit (Cyanistes caeruleus). Heredity **104**:67-78.
103	Portis	2009	Portis, E., G. Mauromicale, R. Mauro, A. Acquadro, D. Scaglione, and S. Lanteri. 2009. Construction of a reference molecular linkage map of globe artichoke (Cynara cardunculus var. scolymus). Theoretical and Applied
104	Harris-Shultz	2010	Harris-Shultz, K. R., B. M. Schwartz, W. W. Hanna, and J. A. Brady. 2010. Development, Linkage Mapping, and Use of Microsatellites in Bermudagrass. Journal of the American Society for Horticultural Science **135**:511-520.
105	Song	2012	Song, W. T., Y. Z. Li, Y. W. Zhao, Y. Liu, Y. Z. Niu, R. Y. Pang, G. D. Miao, X. L. Liao, C. W. Shao, F. T. Gao, and S. L. Chen. 2012. Construction of a High-Density Microsatellite Genetic Linkage Map and Mapping of Sexual and Growth-Related Traits in Half-Smooth Tongue Sole (Cynoglossus semilaevis).
106	Zhao	2013	Zhao, L., Y. Zhang, P. F. Ji, X. F. Zhang, Z. X. Zhao, G. Y. Hou, L. H. Huo, G. M. Liu, C. Li, P. Xu, and X. W. Sun. 2013. A Dense Genetic Linkage Map for Common Carp and Its Integration with a BAC-Based Physical Map. Plos One
107	Zhao	2016	Zhao, X., L. Huang, X. Zhang, J. Wang, D. Yan, J. Li, L. Tang, X. Li, and T. Shi. 2016. Construction of high-density genetic linkage map and identification of flowering-time QTLs in orchardgrass using SSRs and SLAF-seq. Scientific
108	Bradley	2011	Bradley, K. M., J. P. Breyer, D. B. Melville, K. W. Broman, E. W. Knapik, and J. R. Smith. 2011. An SNP-Based Linkage Map for Zebrafish Reveals Sex Determination Loci. G3-Genes Genomes Genetics **1**:3-9.
109	Dukic	2016	Dukic, M., D. Berner, M. Roesti, C. R. Haag, and D. Ebert. 2016. A high-density genetic map reveals variation in recombination rate across the

110	Cristescu	2006	Cristescu, M. E. A., J. K. Colbourne, J. Radivojc, and M. Lynch. 2006. A micro satellite-based genetic linkage map of the waterflea, Daphnia pulex: On the prospect of crustacean genomics. Genomics **88**:415-430.
111	Cavagnaro	2014	Cavagnaro, P. F., M. Iorizzo, M. Yildiz, D. Senalik, J. Parsons, S. Ellison, and P. W. Simon. 2014. A gene-derived SNP-based high resolution linkage map of carrot including the location of QTL conditioning root and leaf anthocyanin
112	Yagi	2013	Yagi, M., T. Yamamoto, S. Isobe, H. Hirakawa, S. Tabata, K. Tanase, H. Yamaguchi, and T. Onozaki. 2013. Construction of a reference genetic linkage map for carnation (Dianthus caryophyllus L.). Bmc Genomics **14**.
113	Chistiakov	2008	Chistiakov, D. A., C. S. Tsigenopoulos, J. Lagnel, Y. M. Guo, B. Hellemans, C. S. Haley, F. A. M. Volckaert, and G. Kotoulas. 2008. A combined AFLP and microsatellite linkage map and pilot comparative genomic analysis of European sea bass Dicentrarchus labrax L. Animal Genetics **39**:623-634.
114	Stocker	2012	Stocker, A. J., B. B. Rusuwa, M. J. Blacket, F. D. Frentiu, M. Sullivan, B. R. Foley, S. Beatson, A. A. Hoffmann, and S. F. Chenoweth. 2012. Physical and Linkage Maps for Drosophila serrata, a Model Species for Studies of Clinal Adaptation and Sexual Selection. G3-Genes Genomes Genetics **2**:287-297.
115	Heesch	2010	Heesch, S., G. Y. Cho, A. F. Peters, G. Le Corguille, C. Falentin, G. Boutet, S. Coedel, C. Jubin, G. Samson, E. Corre, S. M. Coelho, and J. M. Cock. 2010. A sequence-tagged genetic map for the brown alga Ectocarpus siliculosus provides large-scale assembly of the genome sequence. New
116	Blake	2011	Blake, D. P., R. Oakes, and A. L. Smith. 2011. A genetic linkage map for the apicomplexan protozoan parasite Eimeria maxima and comparison with Eimeria tenella. International Journal for Parasitology **41**:263-270.
117	Shirley	2000	Shirley, M. W., and D. A. Harvey. 2000. A genetic linkage map of the apicomplexan protozoan parasite Eimeria tenella. Genome Research **10**:1587-
118	Ting	2016	Ting, N. C., Z. Yaakub, K. Kamaruddin, S. Mayes, F. Massawe, R. Sambanthamurthi, J. Jansen, L. E. T. Low, M. Ithnin, A. Kushairi, X. Arulandoo, R. Rosli, K. L. Chan, N. Amiruddin, K. Sritharan, C. C. Lim, R. Nookiah, M. D. Amiruddin, and R. Singh. 2016. Fine-mapping and cross-validation of QTLs linked to fatty acid composition in multiple independent
119	Montoya	2013	Montoya, C., R. Lopes, A. Flori, D. Cros, T. Cuellar, M. Summo, S. Espeout, R. Rivallan, A. M. Risterucci, D. Bittencourt, J. R. Zambrano, W. H. Alarcon, P. Villeneuve, M. Pina, B. Nouy, P. Amblard, E. Ritter, T. Leroy, and N. Billotte. 2013. Quantitative trait loci (QTLs) analysis of palm oil fatty acid composition in an interspecific pseudo-backcross from Elaeis oleifera (HBK)
120	Mott	2011	Mott, I. W., S. R. Larson, T. A. Jones, J. G. Robins, K. B. Jensen, and M. D. Peel. 2011. A molecular genetic linkage map identifying the St and H subgenomes of Elymus (Poaceae: Triticeae) wheatgrass. Genome **54**:819-
121	Dor	2014	Dor, L., A. Shirak, S. Gorshkov, M. R. Band, A. Korol, Y. Ronin, A. Curzon, G. Hulata, E. Seroussi, and M. Ron. 2014. Construction of a Microsatellites-Based Linkage Map for the White Grouper (Epinephelus aeneus). G3-Genes
122	Swinburne	2006	Swinburne, J. E., M. Boursnell, G. Hill, L. Pettitt, T. Allen, B. Chowdhary, T. Hasegawa, M. Kurosawa, T. Leeb, S. Mashima, J. R. Mickelson, T. Raudsepp, T. Tozaki, and M. Binns. 2006. Single linkage group per chromosome genetic linkage map for the horse, based on two three-
123	Zheng	2013	Zheng, Y. Q., H. L. Guo, G. Z. Zang, and J. X. Liu. 2013. Genetic linkage maps of centipedegrass Eremochloa ophiuroides (Munro) Hack based on sequence-related amplified polymorphism and expressed sequence tag-simple sequence repeat markers. Scientia Horticulturae **156**:86-92.
124	Fukuda	2016	Fukuda, S., K. Ishimoto, S. Sato, S. Terakami, N. Hiehata, and T. Yamamoto. 2016. A high-density genetic linkage map of bronze loquat based on SSR and RAPD markers. Tree Genetics & Genomes **12**.
125	Cui	2015	Cui, Z., M. Hui, Y. Liu, C. Song, X. Li, Y. Li, L. Liu, G. Shi, S. Wang, F. Li, X. Zhang, C. Liu, J. Xiang, and K. H. Chu. 2015. High-density linkage mapping aided by transcriptomics documents ZW sex determination system in the Chinese mitten crab Eriocheir sinensis. Heredity **115**:206-215.
126	RONDEAU	2014	Rondeau, E. B., D. R. Minkley, J. S. Leong, A. M. Messmer, J. R. Jantzen, K. R. von Schalburg, C. Lemon, N. H. Bird, and B. F. Koop. 2014. The Genome and Linkage Map of the Northern Pike (Esox lucius): Conserved Synteny Revealed between the Salmonid Sister Group and the Neoteleostei. Plos One

127	Agrama	2002	Agrama, H. A., T. L. George, and S. F. Salah. 2002. Construction of genome map for Eucalyptus camaldulensis DEHN. Silvae Genetica **51**:201-206.
128	Hudson	2012	Hudson, C. J., J. S. Freeman, A. R. K. Kullan, C. D. Petroli, C. P. Sansaloni, A. Kilian, F. Detering, D. Grattapaglia, B. M. Potts, A. A. Myburg, and R. E. Vaillancourt. 2012. A reference linkage map for Eucalyptus. Bmc Genomics
129	Li	2015	Li, F. G., C. P. Zhou, Q. J. Weng, M. Li, X. L. Yu, Y. Guo, Y. Wang, X. H. Zhang, and S. M. Gan. 2015. Comparative Genomics Analyses Reveal Extensive Chromosome Colinearity and Novel Quantitative Trait Loci in
130	Li	2015	Li, F. G., C. P. Zhou, Q. J. Weng, M. Li, X. L. Yu, Y. Guo, Y. Wang, X. H. Zhang, and S. M. Gan. 2015. Comparative Genomics Analyses Reveal Extensive Chromosome Colinearity and Novel Quantitative Trait Loci in
131	Li	2014	Li, Y., D. W. Wang, Z. Q. Li, J. K. Wei, C. F. Jin, and M. H. Liu. 2014. A Molecular Genetic Linkage Map of Eucommia ulmoides and Quantitative Trait Loci (QTL) Analysis for Growth Traits. International Journal of Molecular
132	Yabe	2014	Yabe, S., T. Hara, M. Ueno, H. Enoki, T. Kimura, S. Nishimura, Y. Yasui, R. Ohsawa, and H. Iwata. 2014. Rapid genotyping with DNA micro-arrays for high-density linkage mapping and QTL mapping in common buckwheat (Fagopyrum esculentum Moench). Breeding Science **64**:291-299.
133	Li	2016	Li, G., L. W. Hillier, R. A. Grahn, A. V. Zimin, V. A. David, M. Menotti-Raymond, R. Middleton, S. Hannah, S. Hendrickson, A. Makunin, S. J. O'Brien, P. Minx, R. K. Wilson, L. A. Lyons, W. C. Warren, and W. J. Murphy. 2016. A High-Resolution SNP Array-Based Linkage Map Anchors a New Domestic Cat Draft Genome Assembly and Provides Detailed Patterns of
134	Wang	2012	Wang, W., Y. Tian, J. Kong, X. Li, X. Liu, and C. Yang. 2012. Integration genetic linkage map construction and several potential QTLs mapping of Chinese shrimp (Fenneropenaeus chinensis) based on three types of
135	Dierking	2015	Dierking, R., P. Azhaguvel, R. Kallenbach, M. Saha, J. Bouton, K. Chekhovskiy, D. Kopecky, and A. Hopkins. 2015. Linkage Maps of a Mediterranean x Continental Tall Fescue Population and their Comparative
136	Backstrom	2008	Backstrom, N., N. Karaiskou, E. H. Leder, L. Gustafsson, C. R. Primmer, A. Qvarnstrom, and H. Ellegren. 2008. A gene-based genetic linkage map of the collared flycatcher (Ficedula albicollis) reveals extensive synteny and gene-order conservation during 100 million years of avian evolution. Genetics
137	Mahoney	2016	Mahoney, L. L., D. J. Sargent, F. Abebe-Akele, D. J. Wood, J. A. Ward, N. V. Bassil, J. F. Hancock, K. M. Folta, and T. M. Davis. 2016. A High-Density Linkage Map of the Ancestral Diploid Strawberry, Fragaria iinumae, Constructed with Single Nucleotide Polymorphism Markers from the IStraw90
138	Davik	2015	Davik, J., D. J. Sargent, M. B. Brurberg, S. Lien, M. Kent, and M. Alsheikh. 2015. A ddRAD Based Linkage Map of the Cultivated Strawberry, Fragaria
139	Sargent	2016	Sargent, D. J., Y. Yang, N. Surbanovski, L. Bianco, M. Buti, R. Velasco, L. Giongo, and T. M. Davis. 2016. HaploSNP affinities and linkage map positions illuminate subgenome composition in the octoploid, cultivated
140	DeVos	2007	De Vos, L., A. A. Myburg, M. J. Wingfield, A. E. Desjardins, T. R. Gordon, and B. D. Wingfield. 2007. Complete genetic linkage maps from an interspecific cross between Fusarium circinatum and Fusarium subglutinans. Fungal
141	Lee	2008	Lee, J., J. E. Jurgenson, J. F. Leslie, and R. L. Bowden. 2008. Alignment of genetic and physical maps of Gibberella zeae. Applied and Environmental
142	Teunissen	2003	Teunissen, H. A. S., M. Rep, P. M. Houterman, B. J. C. Cornelissen, and M. A. Haring. 2003. Construction of a mitotic linkage map of Fusarium oxysporum based on Foxy-AFLPs. Molecular Genetics and Genomics
143	Jurgenson	2002	Jurgenson, J. E., K. A. Zeller, and J. F. Leslie. 2002. Expanded genetic map of Gibberella moniliformis (Fusarium verticillioides). Applied and
144	Hubert	2010	Hubert, S., B. Higgins, T. Borza, and S. Bowman. 2010. Development of a SNP resource and a genetic linkage map for Atlantic cod (Gadus morhua).
145	Pengelly	2016	Pengelly, R. J., A. A. Gheyas, R. Kuo, E. Mossotto, E. G. Seaby, D. W. Burt, S. Ennis, and A. Collins. 2016. Commercial chicken breeds exhibit highly divergent patterns of linkage disequilibrium. Heredity **117**:375-382.
146	Rastas	2016	Rastas, P., F. C. F. Calboli, B. C. Guo, T. Shikano, and J. Merila. 2016. Construction of Ultradense Linkage Maps with Lep-MAP2: Stickleback F-2 Recombinant Crosses as an Example. Genome Biology and Evolution **8**:78-

147	Nakatsuka	2012	Nakatsuka, T., E. Yamada, M. Saito, T. Hikage, Y. Ushiku, and M. Nishihara. 2012. Construction of the first genetic linkage map of Japanese gentian (Gentianaceae). Bmc Genomics **13**.
148	vanderVoort	1999	van der Voort, J., H. J. van Eck, P. M. van Zandvoort, H. Overmars, J. Helder, and J. Bakker. 1999. Linkage analysis by genotyping of sibling populations: a genetic map for the potato cyst nematode constructed using a "pseudo-F2" mapping strategy. Molecular and General Genetics **261**:1021-1031.
149	Song	2016	Song, Q. J., J. Jenkins, G. F. Jia, D. L. Hyten, V. Pantalone, S. A. Jackson, J. Schmutz, and P. B. Cregan. 2016. Construction of high resolution genetic linkage maps to improve the soybean genome sequence assembly
150	Shi	2016	Shi, Y. Z., W. T. Li, A. G. Li, R. H. Ge, B. C. Zhang, J. Z. Li, G. P. Liu, J. W. Li, A. Y. Liu, H. H. Shang, J. W. Gong, W. K. Gong, Z. M. Yang, F. Y. Tang, Z. Liu, W. P. Zhu, J. X. Jiang, X. N. Yu, T. Wang, W. Wang, T. T. Chen, K. B. Wang, Z. S. Zhang, and Y. L. Yuan. 2015. Constructing a high-density linkage map for Gossypium hirsutum x Gossypium barbadense and identifying QTLs
151	Jia	2016	Jia, X. Y., C. Y. Pang, H. L. Wei, H. T. Wang, Q. F. Ma, J. L. Yang, S. S. Cheng, J. J. Su, S. L. Fan, M. Z. Song, N. Wusiman, and S. X. Yu. 2016. High-density linkage map construction and QTL analysis for earliness-related traits
152	Wang	2013	Wang, Z. N., D. Zhang, X. Y. Wang, X. Tan, H. Guo, and A. H. Paterson. 2013. A Whole-Genome DNA Marker Map for Cotton Based on the D-Genome Sequence of Gossypium raimondii L. G3-Genes Genomes Genetics
153	Hou	2013	Hou, M. Y., C. P. Cai, S. W. Zhang, W. Z. Guo, T. Z. Zhang, and B. L. Zhou. 2013. Construction of microsatellite-based linkage map and mapping of nectarilessness and hairiness genes in Gossypium tomentosum. Journal of
154	Ren	2016	Ren, P., W. Z. Peng, W. W. You, Z. K. Huang, Q. Guo, N. Chen, P. R. He, J. W. Ke, J. C. Gwo, and C. H. Ke. 2016. Genetic mapping and quantitative trait loci analysis of growth-related traits in the small abalone Haliotis diversicolor using restriction-site-associated DNA sequencing. Aquaculture **454**:163-170.
155	Vervalle	2013	Vervalle, J., J. A. Hepple, S. Jansen, J. Du Plessis, P. Z. Wang, C. Rhode, and R. Roodt-Wilding. 2013. INTEGRATED LINKAGE MAP OF HALIOTIS MIDAE LINNAEUS BASED ON MICROSATELLITE AND SNP MARKERS.
156	Henning	2017	Henning, F., H. J. Lee, P. Franchini, and A. Meyer. 2014. Genetic mapping of horizontal stripes in Lake Victoria cichlid fishes: benefits and pitfalls of using RAD markers for dense linkage mapping. Molecular Ecology **23**:5224-5240.
157	Henning	2014	Henning, F., G. Machado-Schiaffino, L. Baumgarten, and A. Meyer. 2017. Genetic dissection of adaptive form and function in rapidly-speciating cichlid
158	Celik	2016	Celik, I., S. Bodur, A. Frary, and S. Doganlar. 2016. Genome-wide SNP discovery and genetic linkage map construction in sunflower (Helianthus annuus L.) using a genotyping by sequencing (GBS) approach. Molecular
159	Davey	2016	Davey, J. W., M. Chouteau, S. L. Barker, L. Maroja, S. W. Baxter, F. Simpson, M. Joron, J. Mallet, K. K. Dasmahapatra, and C. D. Jiggins. 2016. Major Improvements to the Heliconius melpomene Genome Assembly Used to Confirm 10 Chromosome Fusion Events in 6 Million Years of Butterfly
160	Atibalentja	2005	Atibalentja, N., S. Bekal, L. L. Domier, T. L. Niblack, G. R. Noel, and K. N. Lambert. 2005. A genetic linkage map of the soybean cyst nematode Heterodera glycines. Molecular Genetics and Genomics **273**:273-281.
161	Shearman	2015	Shearman, J. R., D. Sangsrakru, N. Jomchai, P. Ruang-Areerate, C. Sonthirod, C. Naktang, K. Theerawattanasuk, S. Tragoonrung, and S. Tangphatsornruang. 2015. SNP Identification from RNA Sequencing and Linkage Map Construction of Rubber Tree for Anchoring the Draft Genome.
162	Chen	2011	Chen, M. X., C. L. Wei, J. M. Qi, X. B. Chen, J. G. Su, A. Q. Li, A. F. Tao, and W. R. Wu. 2011. Genetic linkage map construction for kenaf using SRAP, ISSR and RAPD markers. Plant Breeding **130**:679-687.
163	Shirasawa	2015	Shirasawa, K., M. L. Hand, S. T. Henderson, T. Okada, S. D. Johnson, J. M. Taylor, A. Spriggs, H. Siddons, H. Hirakawa, S. Isobe, S. Tabata, and A. M. G. Koltunow. 2015. A reference genetic linkage map of apomictic Hieracium species based on expressed markers derived from developing ovule
164	Palaiokostas	2013	Palaiokostas, C., M. Bekaert, A. Davie, M. E. Cowan, M. Oral, J. B. Taggart, K. Gharbi, B. J. McAndrew, D. J. Penman, and H. Migaud. 2013. Mapping the sex determination locus in the Atlantic halibut (Hippoglossus hippoglossus)

165	Zhou	2016	Zhou, H., S. H. Liu, Y. J. Liu, Y. X. Liu, J. You, M. Deng, J. Ma, G. D. Chen, Y. M. Wei, C. J. Liu, and Y. L. Zheng. 2016. Mapping and validation of major quantitative trait loci for kernel length in wild barley (Hordeum vulgare ssp
166	Henning	2015	Henning, J. A., D. H. Gent, M. C. Twomey, M. S. Townsend, N. J. Pitra, and P. D. Matthews. 2015. Precision QTL mapping of downy mildew resistance in hop (Humulus lupulus L.). Euphytica **202**:487-498.
167	Brelsford	2016	Brelsford, A., C. Dufresnes, and N. Perrin. 2016. High-density sex-specific linkage maps of a European tree frog (Hyla arborea) identify the sex chromosome without information on offspring sex. Heredity **116**:177-181.
168	Guo	2013	Guo, W. J., J. G. Tong, X. M. Yu, C. K. Zhu, X. Feng, B. D. Fu, S. P. He, F. Z. Zeng, X. H. Wang, H. Y. Liu, and L. S. Liu. 2013. A second generation genetic linkage map for silver carp (Hypophthalmichehys molitrix) using microsatellite
169	Bai	2016	Bai, Z. Y., X. K. Han, X. J. Liu, Q. Q. Li, and J. L. Li. 2016. Construction of a high-density genetic map and QTL mapping for pearl quality-related traits in Hyriopsis cumingii. Scientific Reports **6**.
170	Liu	2016	Liu, S., Y. Li, Z. Qin, X. Geng, L. Bao, L. Kaltenboeck, H. Kucuktas, R. Dunham, and Z. Liu. 2016. High-density interspecific genetic linkage mapping provides insights into genomic incompatibility between channel catfish and
171	Zhao	2013	Zhao, N., X. X. Yu, Q. Jie, H. Li, H. Li, J. Hu, H. Zhai, S. Z. He, and Q. C. Liu. 2013. A genetic linkage map based on AFLP and SSR markers and mapping of QTL for dry-matter content in sweetpotato. Molecular Breeding **32**:807-820.
172	Ullmann	2003	Ullmann, A. J., J. Piesman, M. C. Dolan, and W. C. Black. 2003. A preliminary linkage map of the hard tick, Ixodes scapularis. Insect Molecular Biology
173	Wu	2015	Wu, P. Z., C. P. Zhou, S. F. Cheng, Z. Y. Wu, W. J. Lu, J. L. Han, Y. B. Chen, Y. Chen, P. X. Ni, Y. Wang, X. Xu, Y. Huang, C. Song, Z. W. Wang, N. Shi, X. D. Zhang, X. H. Fang, Q. Yang, H. W. Jiang, Y. P. Chen, M. R. Li, Y. Wang, F. Chen, J. Wang, and G. J. Wu. 2015. Integrated genome sequence and linkage map of physic nut (Jatropha curcas L.), a biodiesel plant. Plant
174	Zhu	2015	Zhu, Y. F., Y. F. Yin, K. Q. Yang, J. H. Li, Y. L. Sang, L. Huang, and S. Fan. 2015. Construction of a high-density genetic map using specific length amplified fragment markers and identification of a quantitative trait locus for anthracnose resistance in walnut (Juglans regia L.). Bmc Genomics **16**.
175	Kanomori	2016	Kanamori, A., Y. Sugita, Y. Yuasa, T. Suzuki, K. Kawamura, Y. Uno, K. Kamimura, Y. Matsuda, C. A. Wilson, A. Amores, J. H. Postlethwait, K. Suga, and Y. Sakakura. 2016. A Genetic Map for the Only Self-Fertilizing
176	Robinson	2014	Robinson, N., M. Baranski, K. Das Mahapatra, J. N. Saha, S. Das, J. Mishra, P. Das, M. Kent, M. Arnyasi, and P. K. Sahoo. 2014. A linkage map of transcribed single nucleotide polymorphisms in rohu (Labeo rohita) and QTL associated with resistance to Aeromonas hydrophila. Bmc Genomics **15**.
177	Truco	2013	Truco, M. J., H. Ashrafi, A. Kozik, H. van Leeuwen, J. Bowers, S. R. C. Wo, K. Stoffel, H. Q. Xu, T. Hill, A. Van Deynze, and R. W. Michelmore. 2013. An Ultra-High-Density, Transcript-Based, Genetic Map of Lettuce. G3-Genes
178	Ao	2015	Ao, J. Q., J. Li, X. X. You, Y. N. Mu, Y. Ding, K. Q. Mao, C. Bian, P. F. Mu, Q. Shi, and X. H. Chen. 2015. Construction of the High-Density Genetic Linkage Map and Chromosome Map of Large Yellow Croaker (Larimichthys crocea). International Journal of Molecular Sciences **16**:26237-26248.
179	Guan	2011	Guan, C. Y., H. G. Zhang, L. Zhang, X. F. Li, J. F. Deng, and T. B. Jiang. 2011. CONSTRUCTION OF GENETIC LINKAGE MAPS OF LARCH (LARIX KAEMPFERI x LARIX GMELINI) BY RAPD MARKERS AND MAPPING OF QTLS FOR LARCH. Biotechnology & Biotechnological Equipment **25**:2197-
180	Wang	2017	Wang, L., B. Bai, P. Liu, S. Q. Huang, Z. Y. Wan, E. Chua, B. Ye, and G. H. Yue. 2017. Construction of high-resolution recombination maps in Asian
181	Sudheesh	2016	Sudheesh, S., M. S. Rodda, J. Davidson, M. Javid, A. Stephens, A. T. Slater, N. O. I. Cogan, J. W. Forster, and S. Kaur. 2016. SNP-Based Linkage Mapping for Validation of QTLs for Resistance to Ascochyta Blight in Lentil.
182	Wang	2010	Wang, W. J., H. P. Wang, H. Yao, G. K. Wallat, L. G. Tiu, and Q. Y. Wang. 2010. A first genetic linkage map of bluegill sunfish (Lepomis macrochirus) using AFLP markers. Aquaculture International **18**:825-835.
183	Hawthorne	2001	Hawthorne, D. J. 2001. AFLP-based genetic linkage map of the Colorado potato beetle Leptinotarsa decemlineata: Sex chromosomes and a pyrethroid-resistance candidate gene. Genetics **158**:695-700.

184	Cloutier	2012	Cloutier, S., R. Ragupathy, E. Miranda, N. Radovanovic, E. Reimer, A. Walichnowski, K. Ward, G. Rowland, S. Duguid, and M. Banik. 2012. Integrated consensus genetic and physical maps of flax (Linum usitatissimum
185	Yu	2015	Yu, Y., X. J. Zhang, J. B. Yuan, F. H. Li, X. H. Chen, Y. Z. Zhao, L. Huang, H. K. Zheng, and J. H. Xiang. 2015. Genome survey and high-density genetic map construction provide genomic and genetic resources for the Pacific White
186	Guan	2014	Guan, X. L., M. Hirata, C. L. Ding, N. X. Xu, N. Yuyama, L. B. Tan, Y. C. Fu, J. P. Wang, and H. W. Cai. 2014. Genetic linkage map of Lolium multiflorum Lam. constructed from a BC1 population derived from an interspecific hybridization, L. multiflorum x Lolium temulentum L. x L. temulentum.
187	Velmurugan	2016	Velmurugan, J., E. Mollison, S. Barth, D. Marshall, L. Milne, C. J. Creevey, B. Lynch, H. Meally, M. McCabe, and D. Milbourne. 2016. An ultra-high density genetic linkage map of perennial ryegrass (Lolium perenne) using genotyping by sequencing (GBS) based on a reference shotgun genome assembly.
188	Wang	2008	Wang, X. W., S. Sato, S. Tabata, and S. Kawasaki. 2008. A High-density Linkage Map of Lotus japonicus Based on AFLP and SSR Markers. DNA
189	Berdan	2014	Berdan, E. L., G. M. Kozak, R. Ming, A. L. Rayburn, R. Kiehart, and R. C. Fuller. 2014. Insight Into Genomic Changes Accompanying Divergence: Genetic Linkage Maps and Synteny of Lucania goodei and L. parva Reveal a Robertsonian Fusion. G3-Genes Genomes Genetics **4**:1363-1372.
190	Berdan	2014	Berdan, E. L., G. M. Kozak, R. Ming, A. L. Rayburn, R. Kiehart, and R. C. Fuller. 2014. Insight Into Genomic Changes Accompanying Divergence: Genetic Linkage Maps and Synteny of Lucania goodei and L. parva Reveal a Robertsonian Fusion. G3-Genes Genomes Genetics **4**:1363-1372.
191	Wu	2016	Wu, H., X. He, H. Gong, S. Luo, M. Li, J. Chen, C. Zhang, T. Yu, W. Huang, and J. Luo. 2016. Genetic Linkage Map Construction and QTL Analysis of Two Interspecific Reproductive Isolation Traits in Sponge Gourd. Frontiers in
192	Vipin	2013	Vipin, C. A., D. J. Luckett, J. D. I. Harper, G. J. Ash, A. Kilian, S. R. Ellwood, H. T. T. Phan, and H. Raman. 2013. Construction of integrated linkage map of a recombinant inbred line population of white lupin (Lupinus albus L.).
193	Yang	2013	Yang, H. A., Y. Tao, Z. Q. Zheng, D. Shao, Z. Z. Li, M. W. Sweetingham, B. J. Buirchell, and C. D. Li. 2013. Rapid development of molecular markers by next-generation sequencing linked to a gene conferring phomopsis stem blight disease resistance for marker-assisted selection in lupin (Lupinus
194	Rogers	2006	Rogers, J., R. Garcia, W. Shelledy, J. Kaplan, A. Arya, Z. Johnson, M. Bergstrom, L. Novakowski, P. Nair, A. Vinson, D. Newman, G. Heckman, and J. Cameron. 2006. An initial genetic linkage map of the rhesus macaque (Macaca mulatta) genome using human microsatellite loci. Genomics **87**:30-
195	Wang	2011	Wang, C. W., L. Webley, K. J. Wei, M. J. Wakefield, H. R. Patel, J. E. Deakin, A. Alsop, J. A. M. Graves, D. W. Cooper, F. W. Nicholas, and K. R. Zenger. 2011. A second-generation anchored genetic linkage map of the tammar
196	Zheng	2008	Zheng, Y., G. Zhang, F. C. Lin, Z. H. Wang, G. L. Jin, L. Yang, Y. Wang, X. Chen, Z. H. Xu, X. Q. Zhao, H. K. Wang, J. P. Lu, G. D. Lu, and W. R. Wu. 2008. Development of microsatellite markers and construction of genetic map in rice blast pathogen Magnaporthe grisea. Fungal Genetics and Biology
197	DiPierro	2016	Di Pierro, E. A., L. Gianfranceschi, M. Di Guardo, H. J. J. Koehorst-van Putten, J. W. Kruisselbrink, S. Longhi, M. Troggio, L. Bianco, H. Muranty, G. Pagliarani, S. Tartarini, T. Letschka, L. L. Luis, L. Garkava-Gustavsson, D. Micheletti, M. Bink, R. E. Voorrips, E. Aziz, R. Velasco, F. Laurens, and W. E. van de Weg. 2016. A high-density, multi-parental SNP genetic map on apple validates a new mapping approach for outcrossing species. Horticulture
198	Liu	2016	Liu, Z. C., D. E. Bao, D. L. Liu, and X. S. Chen. 2016. Construction of a genetic linkage map and identification of QTL associated with growth traits in Malus sieversii. Journal of Environmental Biology **37**:1043-1047.
199	Clark	2014	Clark, M. D., C. A. Schmitz, U. R. Rosyara, J. J. Luby, and J. M. Bradeen. 2014. A consensus 'Honeycrisp' apple (Malus x domestica) genetic linkage map from three full-sib progeny populations. Tree Genetics & Genomes
200	Luo	2016	Luo, C., B. Shu, Q. S. Yao, H. X. Wu, W. T. Xu, and S. B. Wang. 2016. Construction of a High-Density Genetic Map Based on Large-Scale Marker Development in Mango Using Specific-Locus Amplified Fragment Sequencing

201	Alaba	2015	Alaba, O. A., J. V. Bredeson, C. N. Egesi, W. Esuma, L. Ezenwaka, M. E. Ferguson, C. M. Ha, M. Hall, L. Herselman, A. Ikpan, E. Kafiriti, E. Kanju, F. Kapinga, A. Karugu, R. Kawuki, B. Kimata, P. Kimurto, P. Kulakow, H. Kulembeka, P. Kusolwa, J. B. Lyons, E. Masumba, A. van de Merwe, G. Mkamilo, A. A. Myburg, A. Nwaogu, I. Nzuki, B. Olasanmi, E. Okogbenin, O. Onyegbule, J. Owuoche, A. Pariyo, S. E. Prochnik, I. Y. Rabbi, D. S. Rokhsar, S. Rounsley, K. Salum, K. S. Shuaibu, C. Sichalwe, M. Stephen, and Icgmc. 2015. High-Resolution Linkage Map and Chromosome-Scale Genome
202	Lu	2016	Lu, X., S. Luan, L. Y. Hu, Y. Mao, Y. Tao, S. P. Zhong, and J. Kong. 2016. High-resolution genetic linkage mapping, high-temperature tolerance and growth-related quantitative trait locus (QTL) identification in Marsupenaeus japonicus. Molecular Genetics and Genomics **291**:1391-1405.
203	Li	2014	Li, X. H., Y. L. Wei, A. Acharya, Q. Z. Jiang, J. M. Kang, and E. C. Brummer. 2014. A Saturated Genetic Linkage Map of Autotetraploid Alfalfa (Medicago sativa L.) Developed Using Genotyping-by-Sequencing Is Highly Syntenous with the Medicago truncatula Genome. G3-Genes Genomes Genetics **4**:1971-
204	Gorton	2012	Gorton, A. J., K. D. Heath, M.-L. Pilet-Nayel, A. Baranger, and J. R. Stinchcombe. 2012. Mapping the Genetic Basis of Symbiotic Variation in Legume-Rhizobium Interactions in Medicago truncatula. G3:
205	Anderson	2016	Anderson, C., M. A. Khan, A. M. Catanzariti, C. A. Jack, A. Nemri, G. J. Lawrence, N. M. Upadhyaya, A. R. Hardham, J. G. Ellis, P. N. Dodds, and D. A. Jones. 2016. Genome analysis and avirulence gene cloning using a high-density RADseq linkage map of the flax rust fungus, Melampsora lini. Bmc
206	Aslam	2010	Aslam, M. L., J. W. M. Bastiaansen, R. Crooijmans, A. Vereijken, H. J. Megens, and M. A. M. Groenen. 2010. A SNP based linkage map of the turkey genome reveals multiple intrachromosomal rearrangements between the Turkey and Chicken genomes. Bmc Genomics **11**.
207	Thomas	2012	Thomas, V. P., S. L. Fudali, J. E. Schaff, Q. L. Liu, E. H. Scholl, C. H. Opperman, D. M. Bird, and V. M. Williamson. 2012. A Sequence-Anchored Linkage Map of the Plant-Parasitic Nematode Meloidogyne hapla Reveals Exceptionally High Genome-Wide Recombination. G3-Genes Genomes
208	Nietlisbach	2015	Nietlisbach, P., G. Camenisch, T. Bucher, J. Slate, L. F. Keller, and E. Postma. 2015. A microsatellite-based linkage map for song sparrows (Melospiza melodia). Molecular Ecology Resources **15**:1486-1496.
209	O'Quin	2013	O'Quin, C. T., A. C. Drilea, M. A. Conte, and T. D. Kocher. 2013. Mapping of pigmentation QTL on an anchored genome assembly of the cichlid fish,
210	McGraw	2011	McGraw, L. A., J. K. Davis, L. J. Young, and J. W. Thomas. 2011. A genetic linkage map and comparative mapping of the prairie vole (Microtus
211	Holeski	2014	Holeski, L. M., P. Monnahan, B. Koseva, N. McCool, R. L. Lindroth, and J. K. Kelly. 2014. A High-Resolution Genetic Map of Yellow Monkeyflower Identifies Chemical Defense QTLs and Recombination Rate Variation. G3-
212	Liu	2016	Liu, S. Y., L. V. Clark, K. Swaminathan, J. M. Gifford, J. A. Juvik, and E. J. Sacks. 2016. High-density genetic map of Miscanthussinensis reveals inheritance of zebra stripe. Global Change Biology Bioenergy **8**:616-630.
213	Morishima	2008	Morishima, K., I. Nakayama, and K. Arai. 2008. Genetic linkage map of the loach Misgurnus anguillicaudatus (Teleostei : Cobitidae). Genetica **132**:227-
214	Samollow	2007	Samollow, P. B., N. Gouin, P. Miethke, S. M. Mahaney, M. Kenney, J. L. VandeBerg, J. A. M. Graves, and C. M. Kammerer. 2007. A microsatellite-based, physically anchored linkage map for the gray, short-tailed Opossum (Monodelphis domestica). Chromosome Research **15**:269-281.
215	Hipplyte	2010	Hippolyte, I., F. Bakry, M. Seguin, L. Gardes, R. Rivallan, A. M. Risterucci, C. Jenny, X. Perrier, F. Carreel, X. Argout, P. Piffanelli, I. A. Khan, R. N. G. Miller, G. J. Pappas, D. Mbeguie-A-Mbeguie, T. Matsumoto, V. De Bernardinis, E. Huttner, A. Kilian, F. C. Baurens, A. D'Hont, F. Cote, B. Courtois, and J. C. Glaszmann. 2010. A saturated SSR/DArT linkage map of
216	Kema	2002	Kema, G. H. J., S. B. Goodwin, S. Hamza, E. C. P. Verstappen, J. R. Cavaletto, T. A. J. Van der Lee, M. de Weerdt, P. J. M. Bonants, and C. Waalwijk. 2002. A combined amplified fragment length polymorphism and randomly amplified polymorphism DNA genetic linkage map of Mycosphaerella graminicola, the septoria tritici leaf blotch pathogen of wheat.

217	Diao	2016	Diao, W., M. Mousset, G. J. Horsburgh, C. J. Vermeulen, F. Johannes, L. van de Zande, M. G. Ritchie, T. Schmitt, and L. W. Beukeboom. 2016. Quantitative Trait Locus Analysis of Mating Behavior and Male Sex Pheromones in Nasonia Wasps. G3-Genes Genomes Genetics **6**:1549-1562.
218	Zhang	2014	Zhang, Q., L. T. Li, R. VanBuren, Y. L. Liu, M. Yang, L. M. Xu, J. E. Bowers, C. H. Zhong, Y. P. Han, S. H. Li, and R. Ming. 2014. Optimization of linkage mapping strategy and construction of a high-density American lotus linkage
219	Liu	2016	Liu, Z., H. Zhu, Y. Liu, J. Kuang, K. Zhou, F. Liang, Z. Liu, D. Wang, and W. Ke. 2016. Construction of a high-density, high-quality genetic map of cultivated lotus (Nelumbo nucifera) using next-generation sequencing. Bmc
220	Zhang	2012	Zhang, S. A., M. Q. Gao, and D. Zaitlin. 2012. Molecular linkage mapping and marker-trait associations with NlRPT, a downy mildew resistance gene in Nicotiana langsdorffii. Frontiers in Plant Science **3**.
221	Gong	2016	Gong, D., L. Huang, X. Xu, C. Wang, M. Ren, C. Wang, and M. Chen. 2016. Construction of a high-density SNP genetic map in fluecured tobacco based on SLAF-seq. Molecular Breeding **36**.
222	Jairin	2013	Jairin, J., T. Kobayashi, Y. Yamagata, S. Sanada-Morimura, K. Mori, K. Tashiro, S. Kuhara, S. Kuwazaki, M. Urio, Y. Suetsugu, K. Yamamoto, M. Matsumura, and H. Yasui. 2013. A Simple Sequence Repeat- and Single-Nucleotide Polymorphism-Based Genetic Linkage Map of the Brown
223	Kirschner	2012	Kirschner, J., D. Weber, C. Neuschl, A. Franke, M. Bottger, L. Zielke, E. Powalsky, M. Groth, D. Shagin, A. Petzold, N. Hartmann, C. Englert, G. A. Brockmann, M. Platzer, A. Cellerino, and K. Reichwald. 2012. Mapping of quantitative trait loci controlling lifespan in the short-lived fish Nothobranchius
224	Ipek	2016	Ipek, A., K. Yilmaz, P. Sikici, N. A. Tangu, A. T. Oz, M. Bayraktar, M. Ipek, and H. Gulen. 2016. SNP Discovery by GBS in Olive and the Construction of a High-Density Genetic Linkage Map. Biochemical Genetics **54**:313-325.
225	McClelland	2008	McClelland, E. K., and K. A. Naish. 2008. A genetic linkage map for coho salmon (Oncorhynchus kisutch). Animal Genetics **39**:169-179.
226	Guyomard	2012	Guyomard, R., M. Boussaha, F. Krieg, C. Hervet, and E. Quillet. 2012. A synthetic rainbow trout linkage map provides new insights into the salmonid whole genome duplication and the conservation of synteny among teleosts.
227	Larson	2016	Larson, W. A., G. J. McKinney, M. T. Limborg, M. V. Everett, L. W. Seeb, and J. E. Seeb. 2016. Identification of Multiple QTL Hotspots in Sockeye Salmon (Oncorhynchus nerka) Using Genotyping-by-Sequencing and a Dense
228	McKinney	2016	McKinney, G. J., L. W. Seeb, W. A. Larson, D. Gomez-Uchida, M. T. Limborg, M. S. O. Brieuc, M. V. Everett, K. A. Naish, R. K. Waples, and J. E. Seeb. 2016. An integrated linkage map reveals candidate genes underlying adaptive variation in Chinook salmon (Oncorhynchus tshawytscha). Molecular Ecology
229	Liu	2013	Liu, F., F. Sun, J. Li, J. H. Xia, G. Lin, R. J. Tu, and G. H. Yue. 2013. A microsatellite-based linkage map of salt tolerant tilapia (Oreochromis mossambicus x Oreochromis spp.) and mapping of sex-determining loci. Bmc
230	Kocher	1998	Kocher, T. D., W. J. Lee, H. Sobolewska, D. Penman, and B. McAndrew. 1998. A genetic linkage map of a cichlid fish, the tilapia (Oreochromis
231	Sternstein	2015	Sternstein, I., M. Reissmann, D. Maj, J. Bieniek, and G. A. Brockmann. 2015. A comprehensive linkage map and QTL map for carcass traits in a cross between Giant Grey and New Zealand White rabbits. Bmc Genetics **16**.
232	Luo	2016	Luo, X. D., J. Zhao, L. F. Dai, F. T. Zhang, Y. Zhou, Y. Wan, and J. K. Xie. 2016. Linkage map construction and QTL mapping for cold tolerance in Oryza rufipogon Griff. at early seedling stage. Journal of Integrative Agriculture
233	DeLeon	2016	De Leon, T. B., S. Linscombe, and P. K. Subudhi. 2016. Molecular Dissection of Seedling Salinity Tolerance in Rice (Oryza sativa L.) Using a High-Density GBS-Based SNP Linkage Map. Rice **9**.
234	Harrang	2015	Harrang, E., S. Heurtebise, N. Faury, M. Robert, I. Arzul, and S. Lapegue. 2015. Can survival of European flat oysters following experimental infection with Bonamia ostreae be predicted using QTLs? Aquaculture **448**:521-530.
235	Johnston	2017	Johnston, S. E., C. Berenos, J. Slate, and J. M. Pemberton. 2016. Conserved Genetic Architecture Underlying Individual Recombination Rate Variation in a Wild Population of Soay Sheep (Ovis aries). Genetics **203**:583-+.

236	Poissant	2010	Poissant, J., J. T. Hogg, C. S. Davis, J. M. Miller, J. F. Maddox, and D. W. Coltman. 2010. Genetic linkage map of a wild genome: genomic structure, recombination and sexual dimorphism in bighorn sheep. Bmc Genomics **11**.
237	Liu	2012	Liu, L. L., Y. Q. Wu, Y. W. Wang, and T. Samuels. 2012. A High-Density Simple Sequence Repeat-Based Genetic Linkage Map of Switchgrass. G3-
238	Winter	2010	Winter, C. B., and A. H. Porter. 2010. AFLP Linkage Map of Hybridizing Swallowtail Butterflies, Papilio glaucus and Papilio canadensis. Journal of
239	Cox	2006	Cox, L. A., M. C. Mahaney, J. L. VandeBerg, and J. Rogers. 2006. A second-generation genetic linkage map of the baboon (Papio hamadryas) genome.
240	Song	2012	Liu, Y. X., H. Z. Han, Q. L. Wang, L. Jiang, S. L. Wang, X. Y. Zhang, Y. Liu, Y. F. Wang, Y. J. Liu, and H. J. Liu. 2013. Choice of microsatellite markers for identifying homozygosity of mitotic gynogenetic diploids in Japanese flounder Paralichthys olivaceus. Journal of Fish Biology **82**:588-599.
241	vanOers	2014	van Oers, K., A. W. Santure, I. De Cauwer, N. E. M. van Bers, R. Crooijmans, B. C. Sheldon, M. E. Visser, J. Slate, and M. A. M. Groenen. 2014. Replicated high-density genetic maps of two great tit populations reveal fine-scale genomic departures from sex-equal recombination rates. Heredity **112**:307-
242	Wang	2017	Wang, S., J. Zhang, W. Jiao, J. Li, X. Xun, Y. Sun, X. Guo, P. Huan, B. Dong, L. Zhang, X. Hu, X. Sun, J. Wang, C. Zhao, Y. Wang, D. Wang, X. Huang, R. Wang, J. Lv, Y. Li, Z. Zhang, B. Liu, W. Lu, Y. Hui, J. Liang, Z. Zhou, R. Hou, X. Li, Y. Liu, H. Li, X. Ning, Y. Lin, L. Zhao, Q. Xing, J. Dou, Y. Li, J. Mao, H. Guo, H. Dou, T. Li, C. Mu, W. Jiang, Q. Fu, X. Fu, Y. Miao, J. Liu, Q. Yu, R. Li, H. Liao, X. Li, Y. Kong, Z. Jiang, D. Chourrout, R. Li, and Z. Bao. 2017. Scallop genome provides insights into evolution of bilaterian karyotype and
243	Baranski	2014	Baranski, M., G. Gopikrishna, N. A. Robinson, V. K. Katneni, M. S. Shekhar, J. Shanmugakarthik, S. Jothivel, C. Gopal, P. Ravichandran, M. Kent, M. Arnyasi, and A. G. Ponniah. 2014. The Development of a High Density Linkage Map for Black Tiger Shrimp (Penaeus monodon) Based on cSNPs.
244	Punnuri	2016	Punnuri, S. M., J. G. Wallace, J. E. Knoll, K. E. Hyma, S. E. Mitchell, E. S. Buckler, R. K. Varshney, and B. P. Singh. 2016. Development of a High-Density Linkage Map and Tagging Leaf Spot Resistance in Pearl Millet Using Genotyping-by-Sequencing Markers. Plant Genome **9**.
245	Kenney-Hunt	2014	Kenney-Hunt, J., A. Lewandowski, T. C. Glenn, J. L. Glenn, O. V. Tsyusko, R. J. O'Neill, J. Brown, C. M. Ramsdell, Q. Nguyen, T. Phan, K. R. Shorter, M. J. Dewey, G. Szalai, P. B. Vrana, and M. R. Felder. 2014. A genetic map of Peromyscus with chromosomal assignment of linkage groups (a Peromyscus
246	Borrone	2009	Borrone, J. W., J. S. Brown, C. L. Tondo, M. Mauro-Herrera, D. N. Kuhn, H. A. Violi, R. T. Sautter, and R. J. Schnell. 2009. An EST-SSR-based linkage map for Persea americana Mill. (avocado). Tree Genetics & Genomes **5**:553-
247	Bossolini	2011	Bossolini, E., U. Klahre, A. Brandenburg, D. Reinhardt, and C. Kuhlemeier. 2011. High resolution linkage maps of the model organism Petunia reveal substantial synteny decay with the related, genome of tomato. Genome
248	Malkus	2009	Malkus, A., Q. Song, P. Cregan, E. Arseniuk, and P. P. Ueng. 2009. Genetic linkage map of Phaeosphaeria nodorum, the causal agent of stagonospora nodorum blotch disease of wheat. European Journal of Plant Pathology
249	Gutierrez	2011	Gutierrez, M. L., and G. Garcia. 2011. A preliminary linkage map using spotted melanic laboratory strains of the livebearing fish Phalloceros caudimaculatus var. reticulata (Cyprinodontiformes: Poeciliidae). Journal of
250	Song	2015	Song, Q. J., G. F. Jia, D. L. Hyten, J. Jenkins, E. Y. Hwang, S. G. Schroeder, J. M. Osorno, J. Schmutz, S. A. Jackson, P. E. McClean, and P. B. Cregan. 2015. SNP Assay Development for Linkage Map Construction, Anchoring Whole-Genome Sequence, and Other Genetic and Genomic Applications in
251	Mathew	2014	Mathew, L. S., M. Spannagl, A. Al-Malki, B. George, M. F. Torres, E. K. Al-Dous, E. K. Al-Azwani, E. Hussein, S. Mathew, K. F. X. Mayer, Y. A. Mohamoud, K. Suhre, and J. A. Malek. 2014. A first genetic map of date palm (Phoenix dactylifera) reveals long-range genome structure conservation in the
252	Kamisugi	2008	Kamisugi, Y., M. von Stackelberg, D. Lang, M. Care, R. Reski, S. A. Rensing, and A. C. Cuming. 2008. A sequence-anchored genetic linkage map for the moss, Physcomitrella patens. Plant Journal **56**:855-866.

253	vanderLee	2004	van der Lee, T., A. Testa, A. Robold, J. van 't Klooster, and F. Govers. 2004. High-density genetic linkage maps of Phytophthora infestans reveal trisomic progeny and chromosomal rearrangements. Genetics **167**:1643-1661.
254	Lind	2014	Lind, M., T. Kallman, J. Chen, X. F. Ma, J. Bousquet, M. Morgante, G. Zaina, B. Karlsson, M. Elfstrand, M. Lascoux, and J. Stenlid. 2014. A Picea abies Linkage Map Based on SNP Markers Identifies QTLs for Four Aspects of Resistance to Heterobasidion parviporum Infection. Plos One **9**.
255	Friesline	2015	Friedline, C. J., B. M. Lind, E. M. Hobson, D. E. Harwood, A. D. Mix, P. E. Maloney, and A. J. Eckert. 2015. The genetic architecture of local adaptation I: the genomic landscape of foxtail pine (Pinus balfouriana Grev. & Balf.) as revealed from a high-density linkage map. Tree Genetics & Genomes **11**.
256	Yang	2013	Yang, H. X., R. Luo, F. C. Zhao, T. Y. Liu, C. X. Liu, and S. W. Huang. 2013. Constructing genetic linkage maps for Pinus elliottii var. elliottii and Pinus caribaea var. hondurensis using SRAP, SSR, EST and ISSR markers. Trees-
257	Chen	2010	Chen, M. M., F. J. Feng, X. Sui, M. H. Li, D. Zhao, and S. J. Han. 2010. Construction of a framework map for Pinus koraiensis Sieb. et Zucc. using SRAP, SSR and ISSR markers. Trees-Structure and Function **24**:685-693.
258	Jermstad	2011	Jermstad, K. D., A. J. Eckert, J. L. Wegrzyn, A. Delfino-Mix, D. A. Davis, D. C. Burton, and D. B. Neale. 2011. Comparative mapping in Pinus: sugar pine (Pinus lambertiana Dougl.) and loblolly pine (Pinus taeda L.). Tree Genetics &
259	Chen	2014	Chen, W. X., M. Cao, Y. X. Wang, Z. C. Zhou, and L. A. Xu. 2014. A genetic linkage map of Pinus massoniana based on SRAP, SSR and ESTP markers.
260	Chancerel	2013	Chancerel, E., J. B. Lamy, I. Lesur, C. Noirot, C. Klopp, F. Ehrenmann, C. Boury, G. Le Provost, P. Label, C. Lalanne, V. Leger, F. Salin, J. M. Gion, and C. Plomion. 2013. High-density linkage mapping in a pine tree reveals a genomic region associated with inbreeding depression and provides clues to
261	Moraga-Suazo	2014	Moraga-Suazo, P., L. Orellana, P. Quiroga, C. Balocchi, E. Sanfuentes, R. W. Whetten, R. Hasbun, and S. Valenzuela. 2014. Development of a genetic linkage map for Pinus radiata and detection of pitch canker disease resistance associated QTLs. Trees-Structure and Function **28**:1823-1835.
262	Westbrook	2015	Westbrook, J. W., V. E. Chhatre, L. S. Wu, S. Chamala, L. G. Neves, P. Munoz, P. J. Martinez-Garcia, D. B. Neale, M. Kirst, K. Mockaitis, C. D. Nelson, G. F. Peter, J. M. Davis, and C. S. Echt. 2015. A Consensus Genetic Map for Pinus taeda and Pinus elliottii and Extent of Linkage Disequilibrium in Two Genotype-Phenotype Discovery Populations of Pinus taeda. G3-Genes
263	Boutet	2016	Boutet, G., S. A. Carvalho, M. Falque, P. Peterlongo, E. Lhuillier, O. Bouchez, C. Lavaud, M. L. Pilet-Nayel, N. Riviere, and A. Baranger. 2016. SNP discovery and genetic mapping using genotyping by sequencing of whole genome genomic DNA from a pea RIL population. Bmc Genomics **17**.
264	Martinello	2005	Martinelli, A., P. Hunt, R. Fawcett, P. V. L. Cravo, D. Walliker, and R. Carter. 2005. An AFLP-based genetic linkage map of Plasmodium chabaudi
265	Jiang	2011	Jiang, H. Y., N. Li, V. Gopalan, M. M. Zilversmit, S. Varma, V. Nagarajan, J. Li, J. B. Mu, K. Hayton, B. Henschen, M. Yi, R. Stephens, G. McVean, P. Awadalla, T. E. Wellems, and X. Z. Su. 2011. High recombination rates and hotspots in a Plasmodium falciparum genetic cross. Genome Biology **12**.
266	Tripathi	2009	Tripathi, N., M. Hoffmann, E. M. Willing, C. Lanz, D. Weigel, and C. Dreyer. 2009. Genetic linkage map of the guppy, Poecilia reticulata, and quantitative trait loci analysis of male size and colour variation. Proceedings of the Royal
267	Sirvio	2011	Sirvio, A., P. Pamilo, R. A. Johnson, R. E. Page, and J. Gadau. 2011. ORIGIN AND EVOLUTION OF THE DEPENDENT LINEAGES IN THE GENETIC CASTE DETERMINATION SYSTEM OF POGONOMYRMEX ANTS.
268	Wang	2010	Wang, Y. X., X. Y. Sun, B. Y. Tan, B. Zhang, L. A. Xu, M. R. Huang, and M. X. Wang. 2010. A genetic linkage map of Populus adenopoda Maxim. x P. alba L. hybrid based on SSR and SRAP markers. Euphytica **173**:193-205.
269	Paolucci	2010	Paolucci, I., M. Gaudet, V. Jorge, I. Beritognolo, S. Terzoli, E. Kuzminsky, R. Muleo, G. S. Mugnozza, and M. Sabatti. 2010. Genetic linkage maps of Populus alba L. and comparative mapping analysis of sex determination across Populus species. Tree Genetics & Genomes **6**:863-875.
270	Mousavi	2016	Mousavi, M., C. F. Tong, F. X. Liu, S. T. Tao, J. Y. Wu, H. G. Li, and J. S. Shi. 2016. De novo SNP discovery and genetic linkage mapping in poplar using restriction site associated DNA and whole-genome sequencing technologies.

271	Gaudet	2008	Gaudet, M., V. Jorge, I. Paolucci, I. Beritognolo, G. S. Mugnozza, and M. Sabatti. 2008. Genetic linkage maps of Populus nigra L. including AFLPs, SSRs, SNPs, and sex trait. Tree Genetics & Genomes **4**:25-36.
272	Pakull	2009	Pakull, B., K. Groppe, M. Meyer, T. Markussen, and M. Fladung. 2009. Genetic linkage mapping in aspen (Populus tremula L. and Populus tremuloides Michx.). Tree Genetics & Genomes **5**:505-515.
273	Liu	2012	Liu, L., J. Li, P. Liu, F. Z. Zhao, B. Q. Gao, and Y. Du. 2012. A genetic linkage map of swimming crab (Portunus trituberculatus) based on SSR and AFLP
274	Feng	2016	Feng, C., C. Feng, and M. Kang. 2016. The first genetic linkage map of Primulina eburnea (Gesneriaceae) based on EST-derived SNP markers.
275	Srinivasan	2002	Srinivasan, J., W. Sinz, C. Lanz, A. Brand, R. Nandakumar, G. Raddatz, H. Witte, H. Keller, I. Kipping, A. Pires-daSilva, T. Jesse, J. Millare, M. de Both, S. C. Schuster, and R. J. Sommer. 2002. A bacterial artificial chromosome-based genetic linkage map of the nematode Pristionchus pacificus. Genetics
276	Lalli	2008	Lalli, D. A., A. G. Abbott, T. N. Zhebentyayeva, M. L. Badenes, V. Damsteegt, J. Polak, B. Krska, and J. Salava. 2008. A genetic linkage map for an apricot (Prunus armeniaca L.) BC(1) population mapping plum pox virus resistance.
277	Wang	2015	Wang, J., K. C. Zhang, X. M. Zhang, G. H. Yan, Y. Zhou, L. B. Feng, Y. Ni, and X. W. Duan. 2015. Construction of Commercial Sweet Cherry Linkage Maps and QTL Analysis for Trunk Diameter. Plos One **10**.
278	Tavassolian	2010	Tavassolian, I., G. Rabiei, D. Gregory, M. Mnejja, M. G. Wirthensohn, P. W. Hunt, J. P. Gibson, C. M. Ford, M. Sedgley, and S. B. Wu. 2010. Construction of an almond linkage map in an Australian population Nonpareil x Lauranne.
279	Cao	2011	Cao, K., L. R. Wang, G. R. Zhu, W. C. Fang, C. W. Chen, and P. Zhao. 2011. Construction of a Linkage Map and Identification of Resistance Gene Analog Markers for Root-knot Nematodes in Wild Peach, Prunus kansuensis. Journal of the American Society for Horticultural Science **136**:190-197.
280	Zhang	2015	Zhang, J., Q. X. Zhang, T. R. Cheng, W. R. Yang, H. T. Pan, J. J. Zhong, L. Huang, and E. Z. Liu. 2015. High-density genetic map construction and identification of a locus controlling weeping trait in an ornamental woody plant (Prunus mume Sieb. et Zucc). DNA Research **22**:183-191.
281	Nunez-Lillo	2015	Nunez-Lillo, G., A. Cifuentes-Esquivel, M. Troggio, D. Micheletti, R. Infante, R. Campos-Vargas, A. Orellana, F. Blanco-Herrera, and C. Meneses. 2015. Identification of candidate genes associated with mealiness and maturity date in peach Prunus persica (L.) Batsch using QTL analysis and deep
282	Arango_Isaza	2016	Arango Isaza, R. E., C. Diaz-Trujillo, B. Dhillon, A. Aerts, J. Carlier, C. F. Crane, T. V. de Jong, I. de Vries, R. Dietrich, A. D. Farmer, C. F. Fereira, S. Garcia, M. Guzman, R. C. Hamelin, E. A. Lindquist, R. Mehrabi, O. Quiros, J. Schmutz, H. Shapiro, E. Reynolds, G. Scalliet, M. Souza, Jr., I. Stergiopoulos, T. A. J. Van der Lee, P. J. G. M. De Wit, M.-F. Zapater, L.-H. Zwiers, I. V. Grigoriev, S. B. Goodwin, and G. H. J. Kema. 2016. Combating a Global Threat to a Clonal Crop: Banana Black Sigatoka Pathogen Pseudocercospora
283	Harel-Beja	2015	Sarkhosh, A., Z. Zamani, R. Fatahi, C. Wiedow, D. Chagne, and S. E. Gardiner. 2012. A pomegranate (Punica granatum L.) linkage map based on AFLP markers. Journal of Horticultural Science & Biotechnology **87**:1-6.
284	Wu	2014	Wu, J., L. T. Li, M. Li, M. A. Khan, X. G. Li, H. Chen, H. Yin, and S. L. Zhang. 2014. High-density genetic linkage map construction and identification of fruit-related QTLs in pear using SNP and SSR markers. Journal of Experimental
285	Bodenes	2016	Bodenes, C., E. Chancerel, F. Ehrenmann, A. Kremer, and C. Plomion. 2016. High-density linkage mapping and distribution of segregation distortion regions in the oak genome. DNA Research **23**:115-124.
286	Cano	2011	Cano, J. M., M. H. Li, A. Laurila, J. Vilkki, and J. Merila. 2011. First-generation linkage map for the common frog Rana temporaria reveals sex-linkage group.
287	Mun	2015	Mun, J. H., H. Chung, W. H. Chung, M. Oh, Y. M. Jeong, N. Kim, B. O. Ahn, B. S. Park, S. Park, K. B. Lim, Y. J. Hwang, and H. J. Yu. 2015. Construction of a reference genetic map of Raphanus sativus based on genotyping by whole-genome resequencing. Theoretical and Applied Genetics **128**:259-272.

288	Steen	1999	Steen, R. G., A. E. Kwitek-Black, C. Glenn, J. Gullings-Handley, W. Van Etten, O. S. Atkinson, D. Appel, S. Twigger, M. Muir, T. Mull, M. Granados, M. Kissebah, K. Russo, R. Crane, M. Popp, M. Peden, T. Matise, D. M. Brown, J. Lu, S. Kingsmore, P. J. Tonellato, S. Rozen, D. Slonim, P. Young, M. Knoblauch, A. Provoost, D. Ganten, S. D. Colman, J. Rothberg, E. S. Lander, and H. J. Jacob. 1999. A high-density integrated genetic linkage and radiation
289	Schlipallus	2002	Schlipalius, D. I., Q. Cheng, P. E. B. Reilly, P. J. Collins, and P. R. Ebert. 2002. Genetic linkage analysis of the lesser grain borer Rhyzopertha dominica identifies two loci that confer high-level resistance to the fumigant
290	Rukam	2016	Rukam, S. T., M. V. Parakhia, J. R. Thakkar, V. M. Rathod, S. M. Padhiyar, V. D. Thummar, H. Dalal, V. V. Kothari, J. V. Kheni, R. M. Dhingani, and B. A. Golakiya. 2016. Development of linkage map and identification of QTLs responsible for fusarium wilt resistance in castor (Ricinus communis L.).
291	Ward	2013	Ward, J. A., J. Bhangoo, F. Fernandez-Fernandez, P. Moore, J. D. Swanson, R. Viola, R. Velasco, N. Bassil, C. A. Weber, and D. J. Sargent. 2013. Saturated linkage map construction in Rubus idaeus using genotyping by sequencing and genome-independent imputation. Bmc Genomics **14**.
292	Bushakra	2012	Bushakra, J. M., M. J. Stephens, A. N. Atmadjaja, K. S. Lewers, V. V. Symonds, J. A. Udall, D. Chagne, E. J. Buck, and S. E. Gardiner. 2012. Construction of black (Rubus occidentalis) and red (R. idaeus) raspberry linkage maps and their comparison to the genomes of strawberry, apple, and
293	Nie	2017	Nie, H., X. Yan, Z. Huo, L. Jiang, P. Chen, H. Liu, J. Ding, and F. Yang. 2017. Construction of a High-Density Genetic Map and Quantitative Trait Locus Mapping in the Manila clam Ruditapes philippinarum. Scientific Reports
294	Zhang	2015	Zhang, N., L. N. Zhang, Y. Tao, L. Guo, J. Sun, X. Li, N. Zhao, J. Peng, X. J. Li, L. Zeng, J. S. Chen, and G. P. Yang. 2015. Construction of a high density SNP linkage map of kelp (Saccharina japonica) by sequencing Taq I site associated DNA and mapping of a sex determining locus. Bmc Genomics **16**.
295	Tsai	2016	Tsai, H. Y., D. Robledo, N. R. Lowe, M. Bekaert, J. B. Taggart, J. E. Bron, and R. D. Houston. 2016. Construction and Annotation of a High Density SNP Linkage Map of the Atlantic Salmon (Salmo salar) Genome. G3-Genes
296	Leitwein	2017	Leitwein, M., B. Guinand, J. Pouzadoux, E. Desmarais, P. Berrebi, and P.-A. Gagnaire. 2017. A Dense Brown Trout (Salmo trutta) Linkage Map Reveals Recent Chromosomal Rearrangements in the Salmo Genus and the Impact of Selection on Linked Neutral
297	Liu	2016	Liu, T., L. L. Guo, Y. L. Pan, Q. Zhao, J. H. Wang, and Z. Q. Song. 2016. Construction of the first high-density genetic linkage map of Salvia miltiorrhiza using specific length amplified fragment (SLAF) sequencing. Scientific
298	Criscione	2009	Criscione, C. D., C. L. L. Valentim, H. Hirai, P. T. LoVerde, and T. J. C. Anderson. 2009. Genomic linkage map of the human blood fluke Schistosoma
299	Hollenbeck	2015	Hollenbeck, C. M., D. S. Portnoy, and J. R. Gold. 2015. A genetic linkage map of red drum (Sciaenops ocellatus) and comparison of chromosomal syntenies with four other fish species. Aquaculture **435**:265-274.
300	Shen	2014	Shen, X. Y., H. Y. Kwan, N. M. Thevasagayam, S. R. S. Prakki, I. S. Kuznetsova, S. Y. Ngoh, Z. Lim, F. Feng, A. Chang, and L. Orban. 2014. The first transcriptome and genetic linkage map for Asian arowana. Molecular
301	Wang	2015	Wang, W. J., Y. L. Hu, Y. Ma, L. Y. Xu, J. T. Guan, and J. Kong. 2015. High-Density Genetic Linkage Mapping in Turbot (Scophthalmus maximus L.) Based on SNP Markers and Major Sex- and Growth-Related Regions
302	Ma	2016	Ma, H. Y., S. J. Li, N. N. Feng, C. Y. Ma, W. Wang, W. Chen, and L. B. Ma. 2016. First genetic linkage map for the mud crab (Scylla paramamosain) constructed using microsatellite and AFLP markers. Genetics and Molecular
303	Milczarski	2015	Milczarski, P., M. Hanek, M. Tyrka, and S. Stojalowski. 2016. The application of GBS markers for extending the dense genetic map of rye (Secale cereale L.) and the localization of the Rfc1 gene restoring male fertility in plants with the C source of sterility-inducing cytoplasm. Journal of Applied Genetics
304	Aoki	2015	Aoki, J., W. Kai, Y. Kawabata, A. Ozaki, K. Yoshida, T. Koyama, T. Sakamoto, and K. Araki. 2015. Second generation physical and linkage maps of yellowtail (Seriola quinqueradiata) and comparison of synteny with four

305	Zhang	2016	Zhang, H. Y., H. M. Miao, C. Li, L. B. Wei, Y. H. Duan, Q. Ma, J. J. Kong, F. F. Xu, and S. X. Chang. 2016. Ultra-dense SNP genetic map construction and identification of SiDt gene controlling the determinate growth habit in
306	Fang	2016	Fang, X. M., K. J. Dong, X. Q. Wang, T. P. Liu, J. H. He, R. Y. Ren, L. Zhang, R. Liu, X. Y. Liu, M. Li, M. Z. Huang, Z. S. Zhang, and T. Y. Yang. 2016. A high density genetic map and QTL for agronomic and yield traits in Foxtail
307	Bergero	2013	Bergero, R., S. Qiu, A. Forrest, H. Borthwick, and D. Charlesworth. 2013. Expansion of the Pseudo-autosomal Region and Ongoing Recombination Suppression in the Silene latifolia Sex Chromosomes. Genetics **194**:673-+.
308	Javidfar	2013	Javidfar, F., and B. F. Cheng. 2013. Construction of a genetic linkage map and QTL analysis of erucic acid content and glucosinolate components in yellow mustard (Sinapis alba L.). Bmc Plant Biology **13**.
309	Viquez-Zamora	2014	Viquez-Zamora, M., M. Caro, R. Finkers, Y. Tikunov, A. Bovy, R. G. F. Visser, Y. L. Bai, and S. van Heusden. 2014. Mapping in the era of sequencing: high density genotyping and its application for mapping TYLCV resistance in
310	Fukuoka	2012	Fukuoka, H., K. Miyatake, T. Nunome, S. Negoro, K. Shirasawa, S. Isobe, E. Asamizu, H. Yamaguchi, and A. Ohyama. 2012. Development of gene-based markers and construction of an integrated linkage map in eggplant by using Solanum orthologous (SOL) gene sets. Theoretical and Applied Genetics
311	vandenOever-vandenElsen	2016	van den Oever-van den Elsen, F., A. F. Lucatti, S. van Heusden, C. Broekgaarden, R. Mumm, M. Dicke, and B. Vosman. 2016. Quantitative resistance against Bemisia tabaci in Solanum pennellii: Genetics and
312	Chen	2014	Chen, A. L., C. Y. Liu, C. H. Chen, J. F. Wang, Y. C. Liao, C. H. Chang, M. H. Tsai, K. K. Hwu, and K. Y. Chen. 2014. Reassessment of QTLs for Late Blight Resistance in the Tomato Accession L3708 Using a Restriction Site Associated DNA (RAD) Linkage Map and Highly Aggressive Isolates of
313	Endelman	2016	Endelman, J. B., and S. H. Jansky. 2016. Genetic mapping with an inbred line-derived F2 population in potato. Theoretical and Applied Genetics **129**:935-
314	Ji	2017	Ji, G. S., Q. J. Zhang, R. H. Du, P. Lv, X. Ma, S. Fan, S. Y. Li, S. L. Hou, Y. C. Han, and G. Q. Liu. 2017. Construction of a high-density genetic map using specific-locus amplified fragments in sorghum. Bmc Genomics **18**.
315	Palaiokostas	2016	Palaiokostas, C., S. Ferraresso, R. Franch, R. D. Houston, and L. Bargelloni. 2016. Genomic Prediction of Resistance to Pasteurellosis in Gilthead Sea Bream (Sparus aurata) Using 2b-RAD Sequencing. G3-Genes Genomes
316	Chan-Navarrete	2016	Chan-Navarrete, R., O. Dolstra, M. van Kaauwen, E. T. L. van Bueren, and C. G. van der Linden. 2016. Genetic map construction and QTL analysis of nitrogen use efficiency in spinach (Spinacia oleracea L.). Euphytica **208**:621-
317	Nemetschke	2010	Nemetschke, L., A. G. Eberhardt, M. E. Viney, and A. Streit. 2010. A genetic map of the animal-parasitic nematode Strongyloides ratti. Molecular and
318	Tortereau	2012	Tortereau, F., B. Servin, L. Frantz, H. J. Megens, D. Milan, G. Rohrer, R. Wiedmann, J. Beever, A. L. Archibald, L. B. Schook, and M. A. M. Groenen. 2012. A high density recombination map of the pig reveals a correlation between sex-specific recombination and GC content. Bmc Genomics **13**.
319	Backstrom	2010	Backstrom, N., W. Forstmeier, H. Schielzeth, H. Mellenius, K. Nam, E. Bolund, M. T. Webster, T. Ost, M. Schneider, B. Kempenaers, and H. Ellegren. 2010. The recombination landscape of the zebra finch Taeniopygia
320	Kai	2005	Kai, W., K. Kikuchi, S. Tohari, A. K. Chew, A. Tay, A. Fujiwara, S. Hosoya, H. Suetake, K. Naruse, S. Brenner, Y. Suzuki, and B. Venkatesh. 2011. Integration of the Genetic Map and Genome Assembly of Fugu Facilitates Insights into Distinct Features of Genome Evolution in Teleosts and
321	Arias	2016	Arias, M., M. Hernandez, N. Remondegui, K. Huvenaars, P. van Dijk, and E. Ritter. 2016. First genetic linkage map of Taraxacum koksaghyz Rodin based on AFLP, SSR, COS and EST-SSR markers. Scientific Reports **6**.
322	Royaert	2016	Royaert, S., J. Jansen, D. V. da Silva, S. M. D. Branco, D. S. Livingstone, G. Mustiga, J. P. Marelli, I. S. Araujo, R. X. Correa, and J. C. Motamayor. 2016. Identification of candidate genes involved in Witches' broom disease resistance in a segregating mapping population of Theobroma cacao L. in

323	Uchino	2016	Uchino, T., Y. Nakamura, M. Sekino, W. Kai, A. Fujiwara, M. Yasuike, T. Sugaya, H. Fukuda, M. Sano, and T. Sakamoto. 2016. Constructing Genetic Linkage Maps Using the Whole Genome Sequence of Pacific Bluefin Tuna (<i>Thunnus orientalis</i>) and a Comparison of Chromosome Structure among Teleost Species. Advances in Bioscience and Biotechnology
324	Foley	2011	Foley, B. R., C. G. Rose, D. E. Rundle, W. Leong, G. W. Moy, R. S. Burton, and S. Edmands. 2011. A gene-based SNP resource and linkage map for the copepod Tigriopus californicus. Bmc Genomics **12**.
325	Lorenzen	2005	Lorenzen, M. D., Z. Doyungan, J. Savard, K. Snow, L. R. Crumly, T. D. Shippy, J. J. Stuart, S. J. Brown, and R. W. Beeman. 2005. Genetic linkage maps of the red hour beetle, Tribolium castaneum, based on bacterial artificial chromosomes and expressed sequence tags. Genetics **170**:741-747.
326	Yezerski	2003	Yezerski, A., L. Stevens, and J. Ametrano. 2003. A genetic linkage map for Tribolium confusum based on random amplified polymorphic DNAs and recombinant inbred lines. Insect Molecular Biology **12**:517-526.
327	Laurent	1998	Laurent, V., E. Wajnberg, B. Mangin, T. Schiex, C. Gaspin, and F. Vanlerberghe-Masutti. 1998. A composite genetic map of the parasitoid wasp Trichogramma brassicae based on RAPD markers. Genetics **150**:275-282.
328	Hirakawa	2016	Hirakawa, H., P. Kaur, K. Shirasawa, P. Nichols, S. Nagano, R. Appels, W. Erskine, and S. N. Isobe. 2016. Draft genome sequence of subterranean clover, a reference for genus Trifolium. Scientific Reports **6**.
329	Griffiths	2013	Griffiths, A. G., B. A. Barrett, D. Simon, A. K. Khan, P. Bickerstaff, C. B. Anderson, B. K. Franzmayr, K. R. Hancock, and C. S. Jones. 2013. An integrated genetic linkage map for white clover (Trifolium repens L.) with
330	Ghamkhar	2012	Ghamkhar, K., S. Isobe, P. G. H. Nichols, T. Faithfull, M. H. Ryan, R. Snowball, S. Sato, and R. Appels. 2012. The first genetic maps for subterranean clover (Trifolium subterraneum L.) and comparative genomics with T. pratense L. and Medicago truncatula Gaertn. to identify new molecular
331	Zhai	2016	Zhai, H. J., Z. Y. Feng, J. Li, X. Y. Liu, S. H. Xiao, Z. F. Ni, and Q. X. Sun. 2016. QTL Analysis of Spike Morphological Traits and Plant Height in Winter Wheat (Triticum aestivum L.) Using a High-Density SNP and SSR-Based
332	MacLeod	2005	MacLeod, A., A. Tweedie, S. McLellan, S. Taylor, N. Hall, M. Berriman, N. M. El-Sayed, M. Hope, C. M. R. Turner, and A. Tait. 2005. The genetic map and comparative analysis with the physical map of Trypanosoma brucei. Nucleic
333	Shan	2015	Shan, T. F., S. J. Pang, J. Li, X. Li, and L. Su. 2015. Construction of a high-density genetic map and mapping of a sex-linked locus for the brown alga Undaria pinnatifida (Phaeophyceae) based on large scale marker development by specific length amplified fragment (SLAF) sequencing. Bmc
334	McCallum	2016	McCallum, S., J. Graham, L. Jorgensen, L. J. Rowland, N. V. Bassil, J. F. Hancock, E. J. Wheeler, K. Vining, J. A. Poland, J. W. Olmstead, E. Buck, C. Wiedow, E. Jackson, A. Brown, and C. A. Hackett. 2016. Construction of a SNP and SSR linkage map in autotetraploid blueberry using genotyping by
335	Covarrubias-Pazaran	2016	Covarrubias-Pazaran, G., L. Diaz-Garcia, B. Schlautman, J. Deutsch, W. Salazar, M. Hernandez-Ochoa, E. Grygleski, S. Steffan, M. Iorizzo, J. Polashock, N. Vorsa, and J. Zalapa. 2016. Exploiting genotyping by sequencing to characterize the genomic structure of the American cranberry
336	Lepers-Andrzejewski	2012	Lepers-Andrzejewski, S., S. Causse, B. Caromel, M. Wong, and M. Dron. 2012. Genetic Linkage Map and Diversity Analysis of Tahitian Vanilla (Vanilla xtahitensis, Orchidaceae). Crop Science **52**:795-806.
337	Sirvio	2011	Sirvio, A., J. S. Johnston, T. Wenseleers, and P. Pamilo. 2011. A high recombination rate in eusocial Hymenoptera: evidence from the common
338	Kaur	2014	Kaur, S., R. B. E. Kimber, N. O. I. Cogan, M. Materne, J. W. Forster, and J. G. Paull. 2014. SNP discovery and high-density genetic mapping in faba bean (Vicia faba L.) permits identification of QTLs for ascochyta blight resistance.
339	Liu	2016	Liu, C. Y., B. J. Fan, Z. M. Cao, Q. Z. Su, Y. Wang, Z. X. Zhang, and J. Tian. 2016. Development of a high-density genetic linkage map and identification of flowering time QTLs in adzuki bean (Vigna angularis). Scientific Reports **6**.
340	Gupta	2008	Gupta, S. K., J. Souframanien, and T. Gopalakrishna. 2008. Construction of a genetic linkage map of black gram, Vigna mungo (L.) Hepper, based on molecular markers and comparative studies. Genome **51**:628-637.

341	Wang	2016	Wang, L. X., C. S. Wu, M. Zhong, D. Zhao, L. Mei, H. L. Chen, S. H. Wang, C. J. Liu, and X. Z. Cheng. 2016. Construction of an integrated map and location of a bruchid resistance gene in mung bean. Crop Journal **4**:360-366.
342	Kongjaimun	2012	Kongjaimun, A., A. Kaga, N. Tomooka, P. Somta, T. Shimizu, Y. J. Shu, T. Isemura, D. A. Vaughan, and P. Srinives. 2012. An SSR-based linkage map of yardlong bean (Vigna unguiculata (L.) Walp. subsp unguiculata Sesquipedalis Group) and QTL analysis of pod length. Genome **55**:81-92.
343	Marubodee	2015	Marubodee, R., E. Ogiso-Tanaka, T. Isemura, S. Chankaew, A. Kaga, K. Naito, H. Ehara, and N. Tomooka. 2015. Construction of an SSR and RAD-Marker Based Molecular Linkage Map of Vigna vexillata (L.) A. Rich. Plos
344	Liu	2013	Liu, Z. D., X. W. Guo, Y. S. Guo, H. Lin, P. X. Zhang, Y. H. Zhao, K. Li, and C. X. Li. 2013. SSR AND SRAP MARKER BASED LINKAGE MAP OF VITIS AMURENSIS RUPR. Pakistan Journal of Botany **45**:191-195.
345	Zhang	2009	Zhang, J. K., L. Hausmann, R. Eibach, L. J. Welter, R. Topfer, and E. M. Zyprian. 2009. A framework map from grapevine V3125 (Vitis vinifera 'Schiava grossa' x 'Riesling') x rootstock cultivar 'Borner' (Vitis riparia x Vitis cinerea) to localize genetic determinants of phylloxera root resistance.
346	Guo	2015	Guo, Y. S., G. L. Shi, Z. D. Liu, Y. H. Zhao, X. X. Yang, J. C. Zhu, K. Li, and X. W. Guo. 2015. Using specific length amplified fragment sequencing to construct the high-density genetic map for Vitis (Vitis vinifera L. x Vitis
347	Wells	2011	Wells, D. E., L. Gutierrez, Z. Xu, V. Krylov, J. Macha, K. P. Blankenburg, M. Hitchens, L. J. Bellot, M. Spivey, D. L. Stemple, A. Kowis, Y. Ye, S. Pasternak, J. Owen, T. Tran, R. Slavikova, L. Tumova, T. Tlapakova, E. Seifertova, S. E. Scherer, and A. K. Sater. 2011. A genetic map of Xenopus
348	Amores	2014	Amores, A., J. Catchen, I. Nanda, W. Warren, R. Walter, M. Schartl, and J. H. Postlethwait. 2014. A RAD-Tag Genetic Map for the Platyfish (Xiphophorus maculatus) Reveals Mechanisms of Karyotype Evolution Among Teleost Fish.
349	Zhou	2016	Zhou, Z. Q., C. S. Zhang, Y. Zhou, Z. F. Hao, Z. H. Wang, X. Zeng, H. Di, M. S. Li, D. G. Zhang, H. J. Yong, S. H. Zhang, J. F. Weng, and X. H. Li. 2016. Genetic dissection of maize plant architecture with an ultra-high density bin map based on recombinant inbred lines. Bmc Genomics **17**.
350	Zhang	2016	Zhang, Z., T. Wei, Y. Zhong, X. Li, and J. Huang. 2016. Construction of a high-density genetic map of Ziziphus jujuba Mill. using genotyping by sequencing technology. Tree Genetics & Genomes **12**.
351	Wang	2015	Wang, F. F., R. Singh, A. D. Genovesi, C. M. Wai, X. E. Huang, A. Chandra, and Q. Y. Yu. 2015. Sequence-tagged high-density genetic maps of Zoysia japonica provide insights into genome evolution in Chloridoideae. Plant
352	Huang	2016	Huang, X. E., F. F. Wang, R. Singh, J. A. Reinert, M. C. Engelke, A. D. Genovesi, A. Chandra, and Q. Y. Yu. 2016. Construction of high-resolution genetic maps of Zoysia matrella (L.) Merrill and applications to comparative genomic analysis and QTL mapping of resistance to fall armyworm. Bmc
353	Lendenmann	2014	Lendenmann, M. H., D. Croll, E. L. Stewart, and B. A. McDonald. 2014. Quantitative Trait Locus Mapping of Melanization in the Plant Pathogenic Fungus Zymoseptoria tritici. G3-Genes Genomes Genetics **4**:2519-2533.
